# High-concentration MEHP triggers mtDNA depletion in undifferentiated HepaRG and C2C12 cultures and disrupts mitochondrial homeostasis in both HepaRG culture states

**DOI:** 10.1093/toxsci/kfag049

**Published:** 2026-04-29

**Authors:** Md Mostafijur Rahman, Pabitra Khadka, Carolyn K J Young, Jing Wang, Elizabeth M McCormick, Marni J Falk, Matthew J Young

**Affiliations:** Division of Biochemistry & Molecular Biology, Department of Biomedical Sciences, Southern Illinois University School of Medicine, Carbondale, IL 62901, United States; Division of Biochemistry & Molecular Biology, Department of Biomedical Sciences, Southern Illinois University School of Medicine, Carbondale, IL 62901, United States; Division of Biochemistry & Molecular Biology, Department of Biomedical Sciences, Southern Illinois University School of Medicine, Carbondale, IL 62901, United States; Division of Genomic Diagnostics, Department of Pathology and Laboratory Medicine, The Children’s Hospital of Philadelphia, Philadelphia, PA 19104, United States; Mitochondrial Medicine Frontier Program, Division of Genetic and Genomic Medicine, Department of Pediatrics, The Children’s Hospital of Philadelphia, Philadelphia, PA 19104, United States; Mitochondrial Medicine Frontier Program, Division of Genetic and Genomic Medicine, Department of Pediatrics, The Children’s Hospital of Philadelphia, Philadelphia, PA 19104, United States; Department of Pediatrics, University of Pennsylvania Perelman School of Medicine, Philadelphia, PA 19104, United States; Division of Biochemistry & Molecular Biology, Department of Biomedical Sciences, Southern Illinois University School of Medicine, Carbondale, IL 62901, United States; Simmons Cancer Institute, Springfield, IL 62702, United States

**Keywords:** phthalates, mono-(2-ethylhexyl) phthalate, apoptosis, mitochondrial toxicity, mitochondrial DNA (mtDNA), mitochondrial homeostasis, autophagy

## Abstract

Phthalates are often called “everywhere chemicals” because they are widely used in consumer products and are detectable in the environment and humans. One of the most studied phthalates, di-2-ethylhexyl phthalate (DEHP), is metabolized to mono-(2-ethylhexyl) phthalate (MEHP), which is known to disrupt metabolic processes through peroxisome proliferator-activated receptor (PPAR) signaling. However, accumulating evidence suggests that lipophilic phthalates also affect mitochondria, key regulators of oxidative metabolism, autophagy, and apoptosis. Based on previous observations that undifferentiated cells are more sensitive to a mitotoxic agent, we hypothesized that MEHP differentially affects mitochondrial function and mitochondrial DNA (mtDNA) maintenance across hepatic cell states. To test this, we used the human HepaRG hepatoma-derived cell line, which can be cultured in undifferentiated and differentiated states, and assessed viability and mitochondrial function following prolonged 6- and 12-d high-concentration MEHP treatments. Prolonged treatments reduced viability and altered bioenergetics in both states. Short treatments (1 to 3 d) reduced viability only in differentiated cultures and were associated with mtDNA depletion in undifferentiated cultures. In both states, MEHP increased the expression of the low-molecular-weight mitochondrial genome maintenance exonuclease (MGME1) isoform, altered the levels of autophagy-related factors, and induced apoptosis. In another mitochondrial-competent myoblast model (C2C12 cells), a high concentration of MEHP was associated with mtDNA depletion, whereas lower concentrations were associated with modest reductions in cell density without detectable mtDNA loss. These results demonstrate state-dependent mitochondrial responses to MEHP and indicate that a reduced endpoint cell density is a sensitive outcome occurring independently of, and at lower concentrations than, mtDNA depletion in undifferentiated cells.

## Graphical Abstract

**Figure:**
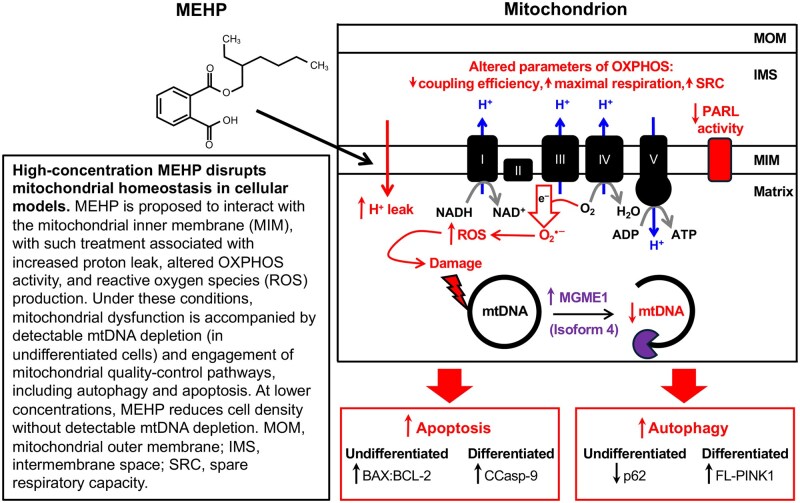

Widespread exposure to phthalates has raised public concern (Li et al. 2014). Phthalates are added to plastics in food packaging, toys, medical devices, and cosmetics, and they leach into food, water, and air (World Health Organization 1996; Liu et al. 2010, 2017a; Lind et al. 2012). The widely studied phthalate, di-2-ethylhexyl phthalate (DEHP), is abundantly used. During initial metabolism within the gut, DEHP is rapidly hydrolyzed to the monoester metabolite mono-(2-ethylhexyl) phthalate (MEHP), which has been widely used to investigate phthalate-associated toxicity in vitro. MEHP has been shown to modulate metabolic processes through peroxisome proliferator-activated receptors (PPARs), which may contribute to the adverse health effects associated with phthalates (Hurst and Waxman 2003; Fan et al. 2010). Furthermore, phthalates can act as endocrine-disrupting chemicals (EDCs) and disrupt hormones like testosterone and estrogen, which are crucial for development and reproduction (Benjamin et al. 2017).

Awareness of the toxic effects of drugs and pollutants on mitochondria is growing. Cationic and lipophilic compounds can disrupt mitochondria owing to the organelle’s negatively charged matrix and lipid-rich composition (Meyer et al. 2013, 2018). Several studies indicate that lipophilic phthalates alter mitochondrial function (Bell 1982; Magdouli et al. 2013; Ban et al. 2014; Benjamin et al. 2017; Chen et al. 2020; Shi et al. 2020; Traore et al. 2021). This is particularly relevant to their endocrine-disrupting effects, because steroidogenic tissues rely on mitochondrial cholesterol metabolism to produce pregnenolone, the precursor of steroid hormones (Schiffer et al. 2019). In mouse Leydig cells, MEHP exposure increased mitochondrial reactive oxygen species (ROS), and decreased LH-induced mitochondrial STAR protein expression, LH-stimulated progesterone production, membrane potential (ΔΨ), and oxygen consumption (Traore et al. 2021). Additionally, DEHP has been demonstrated to cause morphological changes in macaque hepatocyte mitochondria, inhibit nuclear DNA (nDNA) replication, and increase apoptosis in rodent testicular tissues (Satake et al. 2010; Li et al. 2014).

Experimental and epidemiological studies support the association between phthalate exposure and hepatocellular injury, oxidative stress, and disruption of lipid and glucose metabolism, as evidenced by increased liver enzyme levels, histopathological changes, and links to metabolic dysfunction-associated steatotic liver disease (MASLD) (Li et al. 2021; Yu et al. 2021; Aydemir et al. 2023; Sun et al. 2024; Gogola et al. 2025). Because mitochondrial dysfunction is a key mechanism underlying toxicant-induced liver effects, cell-based models remain valuable for investigating mitochondrial toxicity (Attene-Ramos et al. 2013).

To investigate the molecular mechanisms by which MEHP induces mitochondrial toxicity in distinct states, we used the human HepaRG hepatoma-derived cell line, which can be maintained in an undifferentiated proliferative state or differentiated into hepatocyte-like cells (Aninat et al. 2006; Anthérieu et al. 2010). Differentiated HepaRG cultures express cytochrome P450 enzymes and respond to compounds requiring metabolic activation (Aninat et al. 2006; Anthérieu et al. 2010). They recapitulate key aspects of mitochondrial bioenergetics observed in primary human hepatocytes (Peyta et al. 2016). Our previous work established HepaRG as a model for investigating mitochondrial toxicants in both culture states (Young and Young 2019; Young et al. 2021). Undifferentiated HepaRG cells, which model a proliferative progenitor-like state (Guillouzo et al. 2007), are more susceptible to mitotoxic mitochondrial DNA (mtDNA) depletion and loss of viability than their differentiated counterparts (Young et al. 2021). This differential sensitivity suggests that chronic mitotoxic exposure may preferentially impair hepatic regenerative capacity, even if differentiated cells appear more resilient. Because HepaRG cells were derived from a female patient, sex-specific differences reported for MEHP toxicity (e.g., oxidative stress [Davalos et al. 2022]) cannot be evaluated in our model system. Accordingly, this study focuses on cell-state-dependent mechanisms of MEHP-induced mitochondrial toxicity.

We reported that mtDNA is depleted 15 to 21-fold more rapidly in undifferentiated than in differentiated HepaRG after exposure to the mtDNA toxicant ddC (Young et al. 2021), suggesting that undifferentiated cells may preferentially engage mtDNA degradation pathways, including autophagy/mitophagy or nuclease-mediated mechanisms. Despite evidence that MEHP disrupts mitochondrial function, it remains unclear whether MEHP affects mtDNA maintenance pathways in a cell-state-dependent manner in human liver models. We therefore hypothesize that MEHP treatment leads to greater mtDNA depletion in undifferentiated HepaRG; however, because mtDNA replication is independent of the cell cycle, MEHP could also impair mtDNA maintenance in both culture states. To address this gap, we used an in vitro hazard-identification model to determine whether MEHP induces mtDNA depletion and alters the expression of factors involved in autophagy, mtDNA maintenance, and apoptosis in isogenic undifferentiated and differentiated HepaRG cultures. Additionally, to further evaluate MEHP effects on mtDNA maintenance, we used an independent mitochondrial-competent model, mouse C2C12 myoblasts.

## Materials and methods

### HepaRG cell culture and differentiation

The proliferating undifferentiated HepaRG hepatoma-derived cell line was purchased from Biopredic International (Saint-Grégoire, France). Cells were cultured and differentiated as described (Gripon et al. 2002; Aninat et al. 2006; Young and Young 2019; Young et al. 2021). Briefly, the undifferentiated HepaRG cells were cultured in Working Growth Medium (WGM) consisting of William′s Medium E (Thermo Fisher Scientific), 2 mM GlutaMAX (Thermo Fisher Scientific), 50 µM hydrocortisone hemisuccinate (Sigma-Aldrich), 5 µg/ml human recombinant insulin (MilliporeSigma), and 10% fetal bovine serum (FBS) (Corning). Cells were grown at 37 °C and 5% CO_2_ in a humidified incubator, and the medium was refreshed every 3 d. A Leica DMi1 inverted microscope with 10× and 20× phase-contrast objectives was used for day-to-day cell culture examination. Undifferentiated HepaRG cells were passaged between days 12 and 15 post-seeding by washing with prewarmed Dulbecco′s phosphate-buffered saline (DPBS) followed by gentle trypsinization and neutralization with prewarmed WGM. Tissue culture dishes were seeded with 2 × 10^4^ undifferentiated cells/cm^2^, and cells were not passaged more than 18 times (P18).

Working Differentiation Medium (WDM) consisted of the same WGM culture medium supplemented with 2% dimethyl sulfoxide (DMSO; Thermo Fisher Scientific). The differentiation process was started 2 wk after passaging undifferentiated HepaRG cells. The WGM was replaced with Combination Medium (CM), consisting of a 1:1 mixture of WGM and WDM. Three days later, the CM was replaced with WDM. The medium was renewed every 3 d for 2 wk, after which the cells attained a differentiated hepatocyte-like morphology. Following washing with prewarmed DPBS, treatment with prewarmed trypsin, and neutralization with prewarmed medium, HepaRG viable cell counts were determined using the trypan blue exclusion method.

### Determining HepaRG MEHP IC_50_ values

The cytotoxic effect of MEHP on HepaRG cells was initially estimated by measuring the percent growth inhibition relative to untreated control cells. This effect is expressed as the half-maximal inhibitory concentration (IC_50_), or the concentration of MEHP that reduces the number of viable treated cells by 50% compared with untreated control cells. The day before MEHP treatment, undifferentiated HepaRG cells were seeded at 2 × 10^4^ cells/cm^2^ in tissue culture dishes and grown at 37 °C, 5% CO_2_ in a humidified incubator. The next day, cells in replicate dishes were separately exposed to WGM treatment media containing 800, 400, 200, 100, 50, 25, 12.5, or 0 μM MEHP for 7 and 13 d, and the treatment media were refreshed every 3 d. On the day of counting, the treatment media were removed, and the viable cells remaining in each dish were gently washed with DPBS. The cells were then trypsinized, neutralized with their respective treatment medium, and counted using the trypan blue exclusion method. Mock-treated cell counts (0 μM MEHP) were set to 100%, and IC_50_ values were calculated in Prism 7 using nonlinear regression analysis: least-squares fit of inhibitor concentration versus normalized response. For the differentiated HepaRG experiment, proliferating HepaRG cells were seeded at 2 × 10^4^ cells/cm^2^ in 60-mm tissue culture dishes and subjected to the differentiation protocol as described above. Following the differentiation process, cells in each dish were exposed to WDM treatment media containing 800, 400, 200, 100, 50, 25, 12.5, or 0 μM MEHP for 13 d, with the treatment media refreshed every 3 d. Viable differentiated cells were counted, and IC_50_ values were calculated as described above for proliferating HepaRG.

### MEHP treatment of undifferentiated and differentiated-derived HepaRG cells

Undifferentiated HepaRG in WGM were seeded at 2 × 10^4^ cells/cm^2^ for the prolonged treatments (days 6 and 12 of treatment) or at 4 × 10^4^ cells/cm^2^ for the short treatments (days 0, 1, 2, and 3 of treatment) and allowed to adhere to the bottom of culture dishes overnight at 37 °C, 5% CO_2_ in a humidified incubator. WGM treatment media were prepared by adding 200 mM MEHP in DMSO to a final concentration of 300 μM or vehicle (DMSO) only for mock-treated, negative control-treated samples. The DMSO concentration in the WGM + MEHP treatment and negative control media was maintained at 0.4%. The next day, WGM in the culture dishes was replaced with either WGM + vehicle or WGM + 300 μM MEHP treatment media.

For days 6 and 12, prolonged MEHP treatments, undifferentiated cells were seeded in culture dishes, treated as described above, and the treatment media were refreshed every 3 d. A total of 8 dishes were seeded for the experiment, with 2 dishes for the MEHP treatment and 2 dishes for the untreated control on each day.

For short MEHP treatments, days 0 (baseline untreated control), 1, 2, and 3, a total of 12 dishes were seeded for the experiment. On days 0, 1, 2, and 3 post-treatment, 3 dishes were separately harvested.

On the days the undifferentiated cells were harvested, the medium was removed from each tissue culture dish, and the cells were gently washed with prewarmed DPBS. This was followed by trypsinization and neutralization with their respective treatment media. The undifferentiated cells were suspended entirely in treatment media (control or MEHP) + trypsin by gently pipetting up and down (∼10 times) until the mixture appeared homogenous. The cells were transferred to conical tubes, and a small aliquot was removed for counting. The cells were centrifuged at 250 × *g* for 5 min, washed in DPBS (6 ml per 1 × 10^7^ cells), and then suspended in 2 ml DPBS per 1 × 10^7^ cells. One milliliter of the suspension was added to each of two 1.5-ml microcentrifuge tubes. The tubes were centrifuged at 250 g for 5 min at 4 °C, the supernatant was aspirated, and the cell pellets were stored at −80 °C. The experiments were performed 3 times.

Undifferentiated HepaRG cells were differentiated as described above under “HepaRG cell culture and differentiation.” The WDM treatment media were prepared by adding MEHP to a final concentration of 300 μM (the final concentration of DMSO was kept at 2%). Cell culture dishes containing differentiated-derived HepaRG cells were used to separately treat cells with 0 and 300 μM MEHP by replacing the WDM with either WDM + vehicle or WDM + 300 μM MEHP. The treatment media were refreshed every 3 d. For days 6, 12, and 26 of treatment, a total of 12 dishes were seeded for the experiment, with 2 dishes for the MEHP treatment and 2 dishes for the untreated control on each day. On days 6, 12, and 26, 2 control and 2 MEHP-treated dishes were harvested. During the study, we observed that the cells were dying in MEHP treatment dishes; therefore, the number of MEHP-treated dishes was scaled up in the third replicate for a total of 20 dishes (2 control and 4 MEHP-treated 100 mm dishes for days 6 and 12, and 2 control and 6 MEHP-treated 100 mm dishes for day 26).

On days 0, 1, 2, and 3 post-MEHP treatment, 3 of each of the control and MEHP-treated cell culture dishes were harvested, 12 in all.

On the days the differentiated-derived cells were harvested, the treatment media were removed from each dish, and the cells were washed with prewarmed DPBS. This was followed by trypsinization and neutralization with their respective treatment media. The differentiated-derived cells were suspended entirely in the treatment media plus trypsin by gently pipetting up and down (∼10 times) until the mixture appeared homogenous. Cells were counted, washed with DPBS, and frozen at −80 °C as described above for undifferentiated HepaRG. The experiments were performed 3 times.

Undifferentiated and differentiated-derived HepaRG whole-cell DNA was extracted from the frozen cell pellets as described below.

### C2C12 cell culture and MEHP treatment

The C2C12 cell line (ATCC) is a proliferating myoblast (MB) cell line that was derived from C3H mice (Yaffe and Saxel 1977; Blau et al. 1985). During passaging, proliferating C2C12 MB cells were seeded at 5,000 cells/cm^2^ and cultured in a growth medium (GM) composed of Dulbecco’s Modified Eagle’s Medium (DMEM) with 10% FBS at 37 °C in a humidified incubator with 5% CO_2_. The GM was refreshed every 3 d. Daily observations were made using a Leica DMi1 inverted microscope with 10× and 20× phase contrast objectives. The cells were trypsinized between days 3 and 4 post-seeding by first washing with pre-warmed DPBS, then trypsinizing with pre-warmed trypsin and neutralizing with pre-warmed GM. After trypsinization and neutralization, cell counts and viability were determined using the trypan blue exclusion method. Cells were passaged up to 17 times, P17.

For 1- and 2-d treatments with 400 µM MEHP, C2C12 MBs in GM (DMEM/10% FBS) were seeded at 20,000 cells/cm^2^ and allowed to adhere to the bottom of tissue culture dishes overnight at 37 °C and 5% CO_2_ in a humidified incubator. MB treatment media were prepared by adding vehicle or MEHP to the GM to a final concentration of 0 or 400 µM. The day after seeding, the GM was removed from the dishes, and 0 and 400 µM MEHP treatment media were added to separate dishes. Replicate dishes were prepared for harvesting and counting cells on both days 1 and 2. After 1 d of exposure, the treatment media were removed from each tissue culture dish for harvesting and then gently washed separately with pre-warmed DPBS. This was followed by trypsinization and neutralization with their respective treatment media. For each treatment group, the cells were transferred separately to a conical tube, and an aliquot was removed to determine the total cell count using the trypan blue exclusion method. If the cell counts for a sample were less than 100 cells per 1 × 1 mm grid on a hemocytometer, then the cell suspension was pelleted at 250 g for 5 min, the supernatant was aspirated, and the pellet was suspended in a smaller volume of treatment medium to obtain counts of 100 to 300 cells per 1 × 1 mm grid on the hemocytometer. Twenty microliters of the cell suspension was transferred to a microcentrifuge tube, and 20 μl of trypan blue was added. The tube was flicked a few times by fingertip, and 18 μl was loaded into a hemocytometer counting chamber to determine the number of viable (clear) and dead (blue) cells. At least four 1 × 1 mm grids of a hemocytometer counting chamber were counted. The average number of viable cells per grid was used to calculate the concentration, cells/ml.

Next, the cell suspensions were centrifuged at 250 g for 5 min, washed with DPBS (6 ml per 5 × 10^6^ cells), suspended in 1 ml DPBS per 5 × 10^6^ cells, and 1 ml fractions were aliquoted into 1.5-ml microcentrifuge tubes. After centrifugation at 250 g for 5 min at 4 °C, the supernatant was aspirated, and the cell pellets were stored at −80 °C for whole-cell DNA extraction.

After 2 d of MEHP treatment, cells were harvested and counted as described above for the 1-d exposures.

For 10-d MEHP treatments, C2C12 MBs were initially seeded at 160 cells/cm^2^ in GM (DMEM/10% FBS). The next day, the GM was removed, and DMEM/10% FBS MEHP treatment media containing 0, 0.4, 4, and 40 μM MEHP were added to individual dishes and refreshed every 3 d. Following 10 d of treatment, viable cell counts and cell pellets were harvested as described above for the 1- and 2-d treatments.

Note, that all C2C12 MEHP treatment media (and mock-treated controls) contained a final DMSO concentration of 0.4%, and all the experiments were performed 3 times.

### Preparation of HepaRG whole-cell extracted DNA and Southern blot analysis

Proliferating or differentiated-derived HepaRG cell pellets (5 × 10^6^ cells per pellet) were thawed and digested overnight at 55 °C in 500 μl proteinase K digestion buffer (100 mM Tris-Cl, pH 8.5, 5 mM EDTA, 0.2% SDS, 200 mM NaCl, 0.3 mg/ml proteinase K, 1.1 mM 2-mercaptoethanol). The next morning, 10 μl of proteinase K digestion buffer was added to each sample, gently mixed, and incubated for 1 h at 55 °C. Cellular protein was removed from the samples by adding 0.17 ml of 5 M NaCl to each (a final concentration of 1.4 M NaCl), mixing by inversion for 5 min, pelleting at 15,000 g for 15 min, and collecting the supernatant into a fresh microcentrifuge tube. The total nucleic acid was precipitated from the supernatant via ethanol precipitation. The pellets were dried in the dark for 1 h, then resuspended in 1× TE buffer (10 mM Tris-Cl, pH 8.0, 1 mM EDTA) supplemented with 1 mM dimethyl urea (DMU). Samples were stored in the absence of light at room temperature overnight, then frozen at −20 °C. The whole-cell extracted (WCE) DNA concentrations were measured in ≥triplicate using a Qubit fluorometer (Thermo Fisher Scientific) according to the manufacturer’s specifications and as previously described (Wheeler et al. 2019; Young et al. 2021).

HepaRG WCE DNA samples were digested with BamHI (Thermo Scientific), run on agarose gels, subjected to Southern blotting, and analyzed with dual 18S nDNA and mtDNA digoxigenin-labeled probes to determine the mtDNA copy number, as previously described (Wheeler et al. 2019; Young et al. 2021; Rahman et al. 2022).

### Preparation of C2C12 whole-cell extracted DNA

Cell pellets (5 × 10^6^ cells) were thawed and digested overnight at 55 °C with 2 ml proteinase K digestion buffer. The following morning, 40 µl of proteinase K digestion buffer was added to each sample, which was then gently mixed and incubated at 55 °C for 1 h. The lysate was distributed equally among four 1.5 ml Eppendorf tubes, 510 μl per tube. Next, 0.17 ml 5M NaCl was added to each sample tube, mixed by inversion for 5 min, and centrifuged at 15,000 × *g* for 15 min. The 4 supernatants were transferred to new Eppendorf tubes, and total nucleic acid was precipitated from the supernatant via ethanol precipitation. The 4 pellets were dried for about 1 h, and each was suspended in 25 µl of 1× TE buffer + 1 mM DMU + 0.2 mg/ml RNase A. Matched samples were pooled, mixed, and briefly centrifuged. Next, the samples were stored at room temperature in the dark overnight, then transferred to a −20 °C freezer. The WCE DNA concentrations were determined using a Qubit fluorometer as described above.

### Synthesis of digoxigenin-labeled mouse-specific nDNA and mtDNA probes

Plasmid template sequences, each harboring cloned nDNA and mtDNA fragments, were confirmed by Sanger sequencing. The mouse 489 bp 18S rDNA/nDNA fragment was PCR amplified from a C2C12 WCE DNA sample using the Mm18SFor TGG TGC ATG GCC GTT CTT AG and Mm18SRev CTA GAT AGT CAA GTT CGA CC primers and cloned into the pCR2.1-TOPO (Invitrogen) vector according to the manufacturer’s specifications. Similarly, the 490 bp C2C12 16S mtDNA fragment was PCR amplified using the Mm16SFmtDNA GTA AGA ACA AGC AAA GAT TAA ACC T and Mm16SRmtDNA CAG TGT TGC ATC TAT AAA GTT ATA G primers and separately cloned into the pCR2.1-TOPO vector. The pCR2.1-TOPO-Mm18S.nDNA plasmid harbors a fragment of the 18S ribosomal DNA, identical in sequence to the C3H/HeJ *Mus musculus* C3H_HeJ_v3 Assembly, Ensembl Genomic Location CAKLHI020000025.1 66576 to 67064. The pCR2.1-TOPO-Mm16S.mtDNA plasmid harbors a cloned fragment of the mitochondrial 16S ribosomal RNA gene, which corresponds to the C3H/He *M. musculus* mtDNA NCBI Accession No. AB049357.1 nucleotide positions 1244 to 1733. Mouse nDNA and mtDNA digoxigenin (DIG)-labeled probes were synthesized using the PCR DIG Probe Synthesis Kit (Roche) and their respective plasmid templates as described below.

A 250 µl 18S nDNA PCR DIG Probe Synthesis reaction was assembled to include 500 pg pCR2.1-TOPO-Mm18S.nDNA plasmid, 1× PCR buffer with 1.5 mM MgCl_2_, 0.5 µM of each Mm18SFor and Mm18SRev primers, 1× PCR DIG Probe Synthesis mix (200 µM of each of dATP, dCTP, and dGTP, with 130 µM dTTP and 70 µM alkali-labile DIG-11-dUTP), and 52.5 mU/µl Expand High Fidelity enzyme mix. The reaction was aliquoted into ten 0.5 ml PCR tubes and subject to thermocycling. The 18S nDNA cycling conditions were: initial denaturation at 94 °C for 3 min; 32 cycles of denaturation at 94 °C for 45 s, annealing at 58 °C for 30 s, and extension at 72 °C for 1 min; and a final extension at 72 °C for 15 min.

A 250 µl 16S mtDNA PCR DIG Probe Synthesis reaction was assembled to contain 500 pg pCR2.1-TOPO-Mm16S.mtDNA plasmid, 1× PCR buffer with 1.5 mM MgCl_2_, 0.5 µM of each Mm16SFmtDNA and Mm16SRmtDNA primers, 1× PCR DIG Probe Synthesis mix with 1:8 of the alkali-labile DIG-11-dUTP (200 µM of each of dATP, dCTP, and dGTP with 191.25 µM dTTP and 8.75 µM alkali-labile DIG-11-dUTP) and 75 mU/µl of the Expand Long Template PCR System, Enzyme Mix. The reaction was aliquoted into ten 0.5 ml PCR tubes and subject to thermocycling. The 16S mtDNA cycling conditions were: initial denaturation at 94 °C for 3 min; 32 cycles of denaturation at 94 °C for 45 s, annealing at 59 °C for 30 s, and extension at 68 °C for 1 min; and a final extension at 68 °C for 15 min.

The 10 18S nDNA and 10 16S mtDNA DIG-labeled probe PCR reactions were pooled separately into Eppendorf tubes, then boiled in a water bath for 5 min, followed by rapid cooling on ice. Two microliters of the heat-denatured mtDNA probe and 4 µl of the 18S nDNA probe were added per milliliter of 50 °C-prewarmed prehybridization solution (Wheeler et al. 2019) for the simultaneous detection of both loci. This dual probe mixture was stored at −20 °C.

### Southern blot determination of C2C12 mtDNA copy number

To estimate mtDNA copy number, C2C12 WCE DNA was digested with SacI-HF restriction endonuclease (New England Biolabs). SacI cuts at position 9048 of the C3H/He *M. musculus* mtDNA, linearizing it and generating an expected 16.3 kb linear fragment. The fragment is detectable via agarose gel electrophoresis followed by Southern blotting using the Mm16S mtDNA DIG-labeled probe. Also, SacI cuts at the Ensembl Genomic Location C3H_HeJ_v3: CAKLHI020000025.1 SacI sites located at positions 61783 and 68147 to generate an expected ∼6.4 kb nDNA fragment detectable using the Mm18S DIG-labeled probe. One microgram of each WCE DNA sample was digested with 5 units of SacI-HF at 37 °C for 3 h. The digested WCE DNA was then directly loaded into a well of a 1% agarose gel in 1× TAE buffer (40 mM Tris, 20 mM acetic acid, 1 mM EDTA) without ethidium bromide (EtBr). The gel was electrophoresed at 1.05 V/cm for 16 h. The next day, the gel was submerged in 0.5 μg/ml EtBr in TAE buffer and mixed on an orbital shaker at 50 rpm for 30 min. A photograph of the EtBr-stained gel was taken using the Syngene G: Box Chemi XX9 transilluminator. Next, the DNA in the gel was subjected to in-gel depurination, denaturation, Southern blotting, hybridization with dual DIG-labeled probes (16S mtDNA and 18S nDNA), membrane imaging, and band quantification, as described (Wheeler et al. 2019; Young et al. 2021). The mtDNA-specific 16S bands in each lane of the blot were normalized to their respective 18S nDNA signal.

### HepaRG mitochondrial genome sequencing

HepaRG WCE DNA samples were prepared as described above. The entire mtDNA genome was amplified as a single linear amplicon using a high-fidelity long-range PCR (LR-PCR) system (Platinum SuperFi Master Mix, Thermo Fisher Scientific), followed by next-generation sequencing (NGS) with an average sequencing depth of 20,000×. Each sample was tested by 2 independent sets of LR-PCR primers. Comprehensive mtDNA analyses were performed to detect mtDNA single-nucleotide variations (SNVs) and large-scale single and multiple mtDNA deletions by using an in-house custom-built bioinformatics pipeline at the Children’s Hospital of Philadelphia Division of Genomic Diagnostic (DGD) CLIA laboratory within the Department of Pathology. The NGS results from 2 sets of LR-PCR primers were compared. Any low-level heteroplasmic variants that only appear in 1 primer set would be considered sequencing artifacts and removed. The ultra-deep sequencing coverage can accurately detect SNVs with heteroplasmy levels as low as or close to 1% (Wang et al. 2022).

Due to the significant decrease in percent cellular viability of undifferentiated and differentiated-derived HepaRG associated with MEHP as early as 6 d of treatment, which suggests rapid onset phthalate toxicity, we sequenced mtDNA samples from days 0, 3, and 12 of treatment for both cell types, as well as day 26 for the differentiated-derived sample to gain an understanding of whether mtDNA mutations or changes in heteroplasmy are associated with MEHP. The mtDNA genome sequencing FASTQ files were submitted to the NCBI Sequence Read Archive (SRA) under BioProject Accession PRJNA1365961.

### SDS-PAGE and western blotting

Whole-cell protein extracts (WCPEs), SJCRH30 mitochondrial extract, and western blots were prepared according to protocols previously published by our group (Rahman et al. 2022). Briefly,10%, 12%, and 15% SDS-PAGE resolving/5% stacking gels were made, according to Green and Sambrook (Green et al. 2012). For 10- and 15-well gels, 50 and 25 µg of cell extract were loaded per lane, respectively. The bands detected by western blotting (immunoblotting) were normalized to total cellular protein by adding 0.5% 2,2,2-trichloroethanol (TCE) to the resolving gel as described (Ladner et al. 2004). SDS-PAGE gels were run in 1× electrode running buffer (25 mM Tris, 192 mM glycine, 0.1% SDS, pH 8.3) at 100 V for 20 min. Subsequently, the voltage was increased to 200 V for 60, 45, and 60 min, respectively, for 10%, 12%, and 15% gels. The gels were removed from the glass plates and exposed to UV using the Syngene G: Box Chemi XX9 transilluminator for 5 min to visualize and quantify TCE-stained total cellular protein on the nitrocellulose membrane. Next, the gels were equilibrated in transfer buffer (25 mM Tris, 195 mM glycine, 10% 200-proof ethanol) for 15 min. The proteins were transferred to nitrocellulose membranes at 110 V for 2 h in a precooled transfer buffer at 4 °C. After electroblotting, nitrocellulose membranes were imaged using the Syngene G: Box Chemi XX9 transilluminator, and a digital UV image was captured for subsequent normalization of chemiluminescent western blot band intensities to the corresponding TCE-stained total protein signal in each lane.

The nitrocellulose membranes were washed at 60 rpm on a shaker for 5 min in TBST (136.9 mM NaCl, 2.68 mM KCl, 24.77 mM Tris, pH 7.4, 0.1% Tween 20) and blocked for 1 h at room temperature in TBST with 5% nonfat instant dry milk. The blots were washed for 2 min in TBST, then incubated at 4 °C for 16 h with the corresponding primary antibody. All primary antibodies were diluted in TBST + 5% bovine serum albumin (BSA). Primary antibodies used in this study were obtained from Santa Cruz Biotechnology (1/100 dilution of PINK1 mouse monoclonal, Sc-518052; 1/200 of BCL-2 mouse monoclonal, Sc-7382), Cell Signaling Technology (1/500 of p62 rabbit monoclonal, 8025S; 1/500 of DNA polymerase γ/p140 rabbit monoclonal, 13609S; 1/600 of caspase-9 rabbit polyclonal, 9502S; 1/600 of cleaved caspase-9 rabbit polyclonal, 9505S; 1/600 of BAX rabbit polyclonal, 2772S), CUSABIO (1/1000 of TWNK rabbit polyclonal, CSB-PA842754LA01HU), and Protein Tech (1/500 of MGME1 rabbit polyclonal, 23178-1-AP). Next, the blots were washed 3 times with TBST buffer for 10 min, followed by incubation in the appropriate secondary antibody for 1 h, 1/3,000 dilutions of either goat anti-rabbit IgG (H + L) alkaline phosphatase(AP)-conjugated antibody (Bio-Rad) or 1/3,000 dilution of goat anti-mouse IgG (H + L) AP–conjugate (Bio-Rad) in TBST + 5% nonfat instant dry milk. After three 10-min washes in TBST, chemiluminescent bands on the blots were visualized using 1 ml (per full blot) of 0.25 mM CDP-*Star* (Roche) AP substrate in detection buffer (0.1 M Tris-HCl, pH 9.5, 0.1 M NaCl) and a Syngene G: Box Chemi XX9 gel documentation system. Finally, the chemiluminescent band areas in each lane were normalized to their respective TCE-stained total protein signal on the blot. The images used for data analysis, including the TCE-stained blot images and the immunodetected chemiluminescent blot images (Figs S1–S55), were deposited into Dryad, Dataset DOI: 10.5061/dryad.c59zw3rpr.

### Click-iT TUNEL apoptosis assay

The Click-iT TUNEL Alexa Fluor 594 Imaging Assay kit (Thermo Fisher) was used to assess MEHP-induced apoptotic cell death. Undifferentiated and differentiated HepRG cells were treated with 300 µM MEHP for 6 consecutive days. Each day, apoptosis was assessed by harvesting a plate for the Click-iT reaction. For each experiment, undifferentiated HepaRG cells suspended in WGM were separately seeded at 2 × 10^4^ cells/cm^2^ in 8 MatTek dishes and incubated overnight at 37 °C, 5% CO_2_ in a humidified incubator. The next day (day 0 of treatment), the WGM in 6 dishes was replaced with prewarmed MEHP treatment media and returned to the incubator. The remaining 2 dishes were processed and used as a negative control for fluorescence and a day 0 control for TUNEL. The negative control (unstained) dish was subjected to fixation, permeabilization, and the TdT reaction as described below, but the Click-iT reaction and DAPI staining were omitted, and the samples were directly mounted. The second day 0 plate was fixed, permeabilized, and subjected to the TdT reaction, the TUNEL Alexa Fluor 594 Click-iT reaction, stained with DAPI, and mounted (see below). The day 0 control sample was used to determine the background Alexa Fluor/fragmented DNA signal compared with the MEHP-treated samples on days 1 to 6, which were processed identically to the day 0 sample on their respective days. For MEHP-treated plates harvested after day 3, the treatment medium was refreshed on that day.

For the Click-iT TUNEL experiments using differentiated HepaRG cells, the cells were grown and differentiated on gelatin-treated MatTek dishes. The day before seeding, 2 ml of 0.1% gelatin (Millipore Sigma) was added to MatTek dishes and incubated overnight in a humidified 37 °C, 5% CO_2_ incubator. The next day, the gelatin was removed from the dishes, and undifferentiated cells in WGM were seeded at a density of 2 × 10^4^ cells/cm^2^. The WGM was refreshed every 3 d, and after 2 wk of growth, the cells were differentiated as described above. As in the undifferentiated TUNEL experiments described above, 8 dishes were used per experiment, and 2 were processed and used as controls on day 0. The remaining 6 plates were treated with 300 µM MEHP. Every day, 1 plate was removed from the incubator, fixed, permeabilized, and subjected to the TdT reaction, the TUNEL Alexa Fluor 594 Click-iT reaction, stained with DAPI, and then mounted (see below). For dishes harvested on days 4 to 6, the treatment medium was refreshed on day 3.

The plates for an experiment were imaged on the same day after completing the Click-iT reactions, DAPI staining, and mounting. The undifferentiated and differentiated experiments were repeated 3 times using different cell passages. The day 0 background Alexa Fluor signals were negligible in the replicated experiments. Also, the blue and red autofluorescence signals in the unstained negative control dishes were insignificant.

#### Fixation and permeabilization

The medium was removed from a dish, and the cells were washed twice with 2 ml of room-temperature DPBS. Two milliliters of room-temperature 4% paraformaldehyde (PFA) in PBS (Santa Cruz) were added to the plate, and the plate was incubated for 10 min at room temperature. PFA was removed, and cells were washed twice with room temperature DPBS (30 s of mixing at 50 rpm on a mechanical shaker, BT Lab Systems, BT909). Next, the cells were permeabilized using 2 ml of room-temperature 0.5% Triton X-100 in DPBS for 12 min without mixing. The solution was removed, and cells were treated with 2 ml of room-temperature DPBS plus 0.1% Tween 20 for 5 min at 50 rpm. Next, the samples were washed twice for 1 min with sterile Milli-Q water, with mixing, and then incubated with 2 ml of ice-cold methanol for 10 min at −20 °C. Finally, the cells were washed twice with room-temperature Milli-Q water for 1 min with mixing.

#### TdT reaction

The TdT reaction cocktail containing buffer, EdUTP, and TdT was prepared according to the manufacturer’s instructions. Briefly, after fixation and permeabilization, 100 μl of TdT reaction buffer was added to a MatTek dish microwell, and the dish was incubated at room temperature for 10 min. Next, the reaction buffer was removed, and 100 µl of TdT reaction cocktail was added to the microwell. A wet paper towel was placed below and above the plate to reduce evaporation, and the mixture was then incubated in a humidified non-CO_2_ incubator at 37 °C for 1 h. The reaction cocktail was then removed, and the plate was washed twice with 2 ml of 5% BSA in DPBS for 15 min with mixing at 50 rpm.

#### Click-iT reaction

The Click-iT reaction cocktail containing Alexa Fluor 594 azide, reaction buffer, and the reaction buffer additive was prepared according to the manufacturer’s instructions. One hundred microliters of the reaction cocktail was added to a MatTek dish microwell and incubated at room temperature in the dark for 30 min. Next, the reaction cocktail was removed, and the sample was washed 3 times with 5% BSA in DPBS + 0.1% Tween 20 for 10 min at 50 rpm. The dish was covered to protect it from light during washing.

#### DAPI staining

Following the Click-iT reaction, plates were equilibrated with 2 ml DPBS. One hundred microliters of 0.105 µg/ml DAPI staining solution in DPBS was added to a MatTek microwell and incubated for 15 min in the dark. The DAPI staining solution was removed, and the samples were washed twice with 2 ml DPBS before mounting.

#### Mounting MatTek dishes

ProLong Glass Antifade Mountant (ThermoFisher) was allowed to warm to room temperature for 1 h before mounting specimens. One drop of the mountant was added to a MatTek dish microwell, and a 10 mm round coverslip was quickly and carefully put on top using tweezers to avoid bubble formation. Next, to allow the mountant to cure, the MatTek dish was placed in a dark cabinet overnight at room temperature.

#### Fluorescence microscopy

Fluorescent images were collected using a Leica DMi8 epifluorescence microscope with a Lumencor SOLA light engine, a 63×, 1.4 numerical aperture oil objective, and a Hamamatsu ORCA-Flash4.0 V2 digital scientific complementary metal-oxide-semiconductor (sCMOS) camera. The exposure times for the DAPI (blue fluorescence) and Alexa Fluor 594 (red fluorescence) channels were empirically determined in a separate experiment using DNase I-treated positive control samples as recommended by the manufacturer. For visualization of nuclei, a DAPI filter cube (Excitation: 350/50; Dichroic: 400; Emission: 460/50; 100 ms exposure time) was used. For visualization of TUNEL/Alexa Fluor 594, a TXR filter cube (Excitation: 560/40; Dichroic: 585; Emission: 630/75; 40 ms exposure time) was used. For dual imaging of DAPI and Alexa Fluor 594, images were collected in sequence: differential interference contrast (DIC; channel 1), then DAPI (channel 2), and finally TXR (channel 3). A negative control plate was imaged to determine and subtract background fluorescence. Nuclei were counted in each image and divided into 2 categories: (i) Viable non-apoptotic counts that are DAPI-positive only, and (ii) Apoptotic counts that are TUNEL/Alexa Fluor 594 and DAPI positive. Three images were counted for each treatment type and from each experiment. Linear adjustments were made in LAS X, and the same software was used to generate scale bars.

### Seahorse XFp bioenergetic analysis

The Seahorse XFp extracellular flux analyzer was used to measure HepaRG bioenergetics as previously described (Young and Young 2019). Briefly, 1 × 10^4^ undifferentiated or differentiated-derived HepaRG cells per well were subjected to Cell Mito Stress Tests by treating sequentially with 2 µM oligomycin, 1 µM carbonyl cyanide *p*-trifluoromethoxy-phenylhydrazone (FCCP), and 0.5 µM antimycin A + 0.5 µM rotenone (all purchased from Fisher Scientific). Following the experiments, cells were gently washed with 200 µl pre-warmed DPBS, incubated overnight at −80 °C, then lysed in ice-cold RIPA lysis buffer (50 mM Tris-Cl pH 8.0, 150 mM NaCl, 1% Igepal CA-630, 0.5% deoxycholate, 0.1% sodium dodecyl sulfate) supplemented with a 1 in 101 dilution of HALT protease inhibitor cocktail (Thermo Fisher Scientific). The total cellular protein content in each miniplate well was measured using the Pierce Bicinchoninic Acid (BCA) Protein Assay Kit (Thermo Scientific). OCR and ECAR values were then normalized to total cellular protein using Seahorse Wave Desktop Software. Data were obtained from at least 3 independent experiments performed on different days and are presented as mean values ± standard deviation (SD).

#### Equations used to calculate mitochondrial respiration parameters

Mitochondrial respiration parameters were calculated as follows: *I. Basal respiration* = (Last rate before the first injection) − (Non-mitochondrial respiration rate), *II. Proton leak* = (Minimum rate after oligomycin injection) − (Non-mitochondrial respiration rate), *III. Maximal respiratory capacity* = (Maximum rate after FCCP injection) − (Non-mitochondrial respiration rate), *IV. Spare respiratory capacity (SRC)* = (Maximal respiratory capacity) − (Basal respiration), *V. Non-mitochondrial respiration* = Minimum rate after rotenone + antimycin A injection, *VI. ATP-linked respiration* = (Last rate before oligomycin injection) − (Minimum rate after oligomycin injection), *VII. Coupling efficiency = (ATP-linked respiration)/(Basal respiration)*, (Divakaruni et al. 2014; Young and Young 2019).

### Simplified calculation to estimate the effect of serum protein binding on unbound MEHP in vitro

To aid in the interpretation of nominal *in vitro* MEHP concentrations, we performed a simplified calculation to estimate the fraction of MEHP that is unbound under typical serum-containing culture conditions. This calculation is intended to illustrate the potential influence of protein binding on freely available MEHP and does not constitute a formal in vitro-to-in vivo extrapolation or risk assessment.

The unbound fraction of MEHP in culture medium (ƒ_u,medium_) was estimated using a previously described binding-dilution equation (Liu et al. 2014; Munic Kos et al. 2025):


$$f_{u,\text{medium}}=\frac{1}{(\text{DF})(\frac{1}{f_{u,p}}-1)+1}$$

Where the plasma unbound fraction (ƒ_*u,p*_) was estimated as 0.007 (Adachi et al. 2015), and the dilution factor (DF) reflects the 10% fetal bovine serum present in WGM and WDM. Using these values, the ƒ_*u*__,medium_ was estimated to be approximately 0.066. Because the ƒ_*u,p*_ value was derived from chimeric mice transplanted with human hepatocytes, this estimate should be interpreted cautiously, as species-specific differences in protein composition, as well as differences between plasma and serum, may contribute to uncertainty.

The estimated free concentration in culture medium was calculated as *C*_free_ = *C*_nominal_ × ƒ_*u*__,medium_ such that a nominal concentration of 300 µM corresponds to an estimated free concentration of 19.8 µM (5.5 mg/l). This estimate accounts only for protein binding and does not consider additional losses due to adsorption onto plasticware or cellular uptake.

### Statistical analyses

All data presented are mean values ± standard deviations (SDs). Statistical significance between 2 parametric groups was determined using a Student’s or a Welch’s *t*-test, and significance between 2 nonparametric groups was determined using a Mann–Whitney *U* test. Comparisons of more than 2 groups of parametric data were assessed by one-way analysis of variance (ANOVA) followed by Tukey’s test or Welch’s ANOVA with Dunnett T3 post hoc test. Comparisons of greater than 2 groups of nonparametric data were assessed by Kruskal–Wallis tests followed by Dunn’s post hoc test. *P*-values less than 0.05 were considered significant.

## Results

### Undifferentiated- and differentiated-derived HepaRG have similar MEHP IC_50_ values

The estimated daily intake of DEHP among occupational workers ranges from 0.6 to 850 µg·kg^−1^·d^−1^ (Hines et al. 2011), and representative civilian sampling shows urinary MEHP levels in the nM range (Barr et al. 2003). Additionally, rare contamination events can lead to elevated phthalate exposure. For example, during the 2011 Taiwan food scandal, several beverages contained DEHP with reported concentrations as high as ∼90 µM, demonstrating that exceptionally high oral exposures can occur under non-regulatory conditions (Yang et al. 2013). Clinically exposed groups, including blood transfusion patients and infants in intensive care, have been reported to reach plasma MEHP concentrations of 50 to 54 µM (Wang et al. 2012), and food contamination events have revealed DEHP levels of 80 to 90 µM in high-fat items such as milk and cheese (World Health Organization 1996). These findings demonstrate that humans can intermittently encounter phthalate levels above those measured in general population surveys. Based on these observations, MEHP concentrations of ∼70–1000 µM have been used across different model systems for hazard identification (Yokoyama et al. 2003; Hannon et al. 2015; Chen et al. 2020; Arcanjo et al. 2023).

**Fig. 1.**
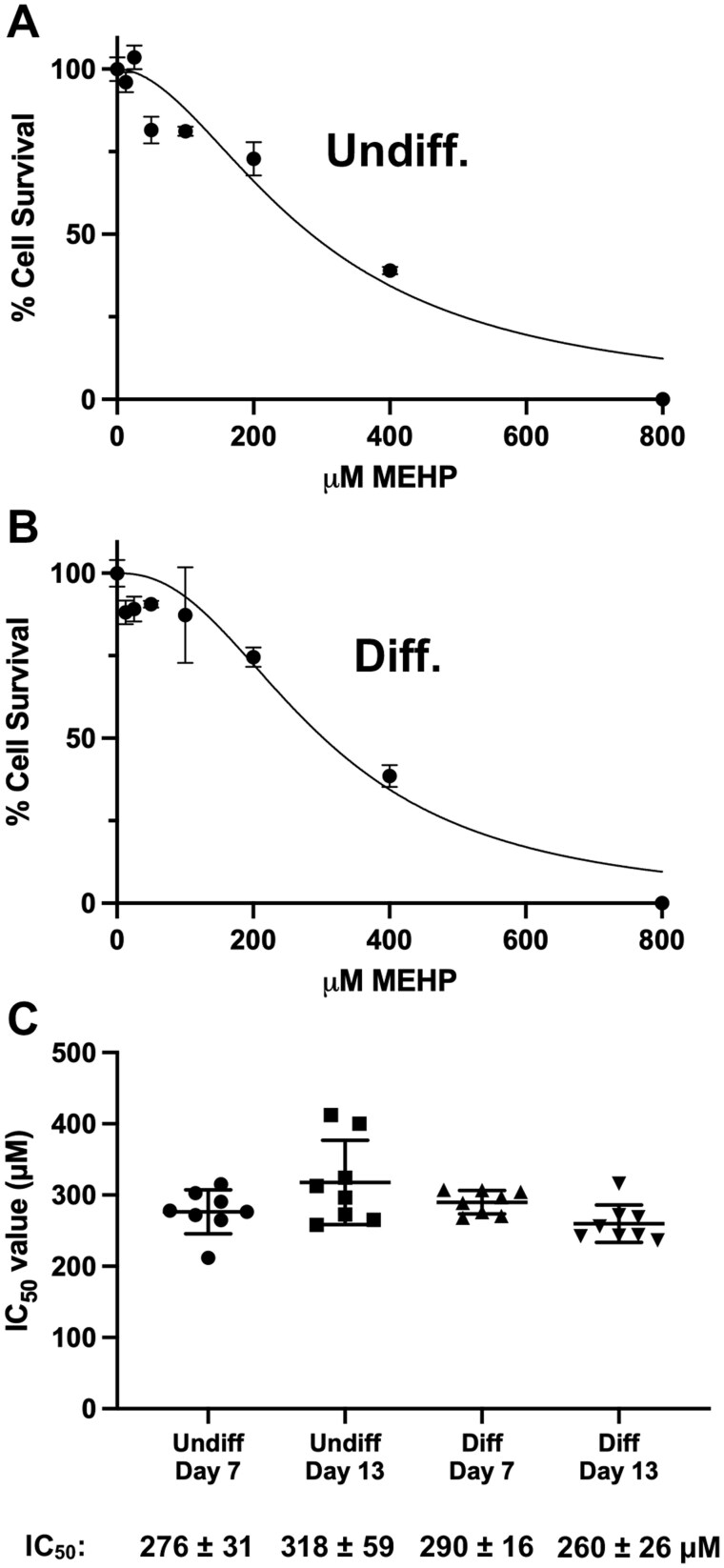
Undifferentiated and differentiated-derived HepaRG cells show similar sensitivity to MEHP. Representative, (A) undifferentiated- and (B) differentiated-derived HepaRG culture survival curves following MEHP treatments. Cells were treated with 800, 400, 200, 100, 50, 25, 12.5, and 0 μM MEHP for 7 d, and mean % survival values (*n* ≥ 3) and standard deviations (SDs) are reported for each concentration. (C) MEHP IC_50_ values for undifferentiated- and differentiated-derived HepaRG cultures were calculated by least-squares fitting of inhibitor concentration versus normalized response in GraphPad Prism at days 7 and 13 post-treatment. The mean IC_50_ values from 2 independent experiments using undifferentiated- and differentiated-derived HepaRG at 2 different passages are reported in micromolar (μM) with error as SD.

We used a hazard identification approach to assess the adverse effects of MEHP on various mitochondrial functions. A vehicle control and MEHP at concentrations ranging from 12.5 to 800 µM were used to determine half-maximal inhibitory concentration (IC_50_) values. Following 7-d MEHP treatments, we observed a recognizable sigmoidal relationship between [MEHP] and % cell survival in both undifferentiated and differentiated cells (Fig. 1A and B). The undifferentiated and differentiated cell IC_50_ values determined following 7- and 13-d treatments were similar, ranging from 260 to 318 µM (Fig. 1C). These values align with previous studies reporting similar values for TK6 cells (250 µM; Rosado-Berrios et al. 2011), HEK-293 (257 µM; Ashari et al. 2023), and C2C12 (∼300 µM; Chen et al. 2020).

### Six and 12 d of MEHP treatment alter the growth and survival of HepaRG cells

**Fig. 2.**
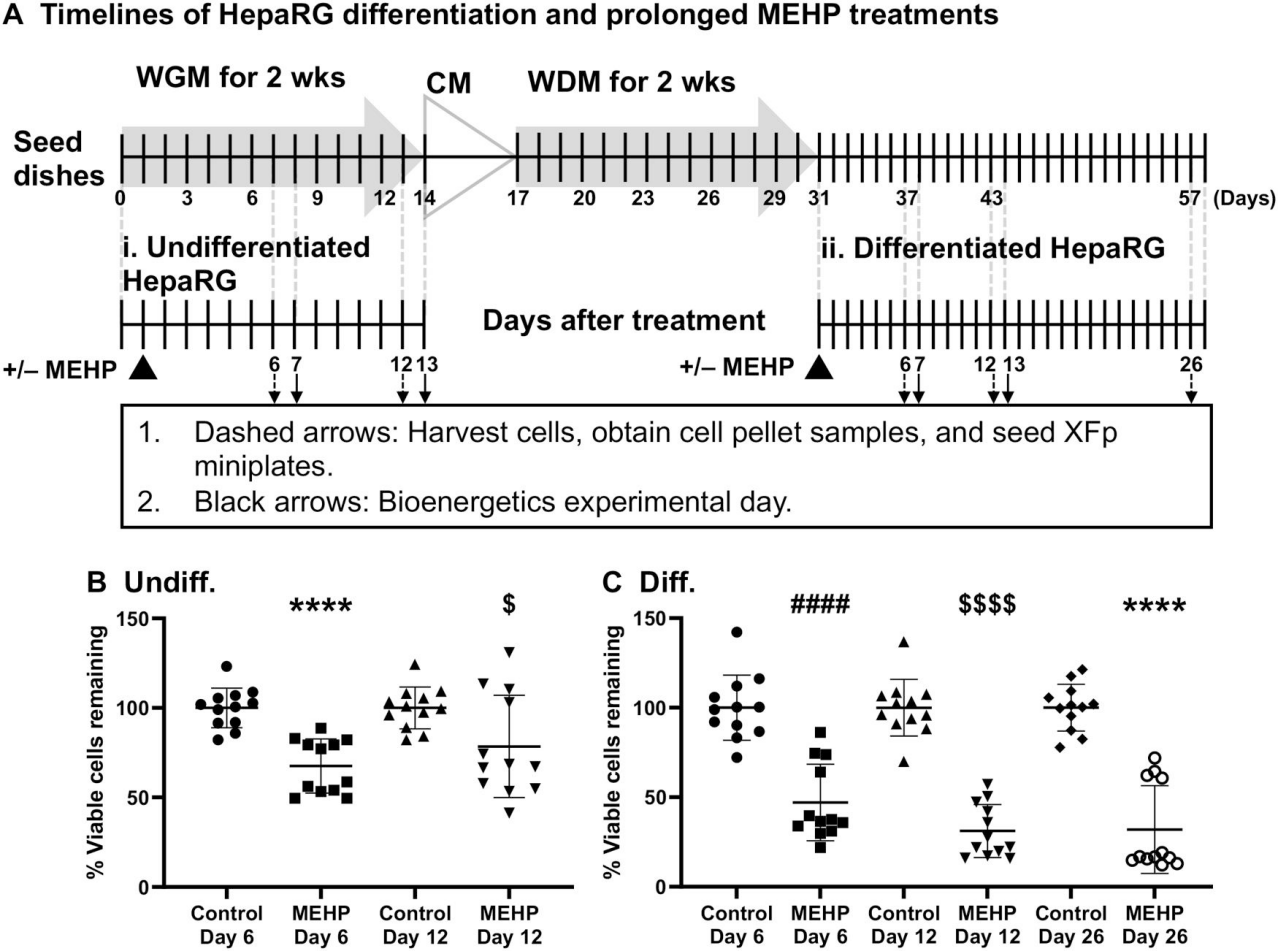
HepaRG viable cell counts are altered following prolonged MEHP treatments. (A) Timelines of HepaRG differentiation and prolonged MEHP treatment experiments for undifferentiated and differentiated-derived HepaRG. Undifferentiated cells were initially cultured in Working Growth Medium (WGM), which was replaced with Combination Medium (CM), followed by Working Differentiation Medium (WDM) as outlined in Materials and Methods. Relative viable cell counts from (B) undifferentiated- (Undiff.) and (C) differentiated-derived (Diff.) HepaRG cultures at various time points following MEHP treatments. For MEHP treatments following 6, 12, and 26 d, matched 0 µM vehicles were used as controls for each time point. The number of viable cells remaining on the cell culture dishes was determined utilizing the trypan blue exclusion method. In each set of experiments, vehicle control-treated cell counts were set to 100%, and relative MEHP-treated counts were determined. Data are presented as mean viable cell counts ± SD, *n* = 12 (quadruplicate from 3 independent experiments using different cell passages). Unpaired t-test with corrections for parametric data or Mann-Whitney U tests for nonparametric data were used. ****P ≤ 0.0001, $*P* < 0.05, ####*P* ≤ 0.0001, $$$$*P* ≤ 0.0001.

Undifferentiated and differentiated HepaRG cells were treated with 300 μM MEHP or vehicle control medium for 6 and 12 d, and then harvested to determine cellular viability, seed miniplates for bioenergetics studies, and obtain cell pellets for molecular biology analyses (Fig. 2A). Following the treatments, cell culture counts were obtained, as shown in Fig. 2B and C. The percentage of undifferentiated viable cells remaining on tissue culture dishes following 6-d MEHP treatments was approximately one-third less than that of the control (68 ± 15% vs. 100 ± 11% viable cells remaining) (Fig. 2B). On day 12 of treatment, the percentage of cells remaining in the undifferentiated HepaRG culture was 79 ± 29%, approximately one-fifth lower than in the control (100 ± 12%).

Differentiated HepaRG cultures contain non-dividing cells that can be maintained in tissue culture for 4 wk in a working differentiation medium (WDM) (Anthérieu et al. 2010). At 6, 12, and 26 d of MEHP treatment, the percent viability decreased to 47 ± 21%, 31 ± 15%, and 32 ± 25%, respectively, relative to their matched vehicle controls (Fig. 2C).

Taken together, these data suggest that during prolonged MEHP treatment, both cell types die and detach from the bottom of the dishes by day 6; however, the greater loss of differentiated-derived cell viability indicates that differentiated cells are most sensitive to MEHP.

### Seven- and 13-d MEHP treatments alter HepaRG mitochondrial bioenergetics

We suspect that MEHP and other phthalates partition into the lipid-rich mitochondrial membranes (Nazir et al. 1971, 1973) because their structures combine a hydrophobic aromatic ring with 1 or 2 lipophilic alkyl side chains linked via an ester bond. Driven by oxidative phosphorylation (OXPHOS)-mediated proton pumping, the mitochondrial intermembrane space is more acidic (pH ∼6.8) than the cytosol (pH 7.4) (Santo-Domingo and Demaurex 2012). Under these conditions, weakly acidic compounds such as MEHP may exhibit modest shifts toward a more neutral (non-ionized) state, thereby increasing lipophilicity and membrane partitioning. For example, using the equation log *D* = XLog*P3 *+ log[1/(1 + 10^pH − pKa^)] (Kujawski et al. 2012), the estimated distribution coefficient (log *D*) for MEHP increases from −0.32 at pH 7.4 to 0.28 at pH 6.8 (a 0.6 unit increase), calculated based on a pKa of 3.08 (Jornet-Martinez et al. 2015) and XLog*P3* of 4.0 (PubChem 2025). These considerations suggest that MEHP may preferentially partition into mitochondrial lipid bilayers, providing a physicochemical rationale for a potential interaction with mitochondrial membranes. Such interactions may influence the function of mitochondrial inner membrane (MIM)-anchored mtDNA nucleoids (Ishihara et al. 2022) and the densely packed MIM-localized OXPHOS machinery (Hirst 2018). However, while these physicochemical properties may facilitate membrane partitioning, the extent of mitochondrial dysfunction is likely concentration-dependent and may require sufficient accumulation to disrupt mitochondrial processes. To determine whether MEHP alters HepaRG OXPHOS and bioenergetics, as well as other mitochondrial metabolic processes, such as mtDNA maintenance, we quantified mitochondrial bioenergetic parameters, mtDNA copy number, mtDNA variants, and replisome factors.

**Fig. 3.**
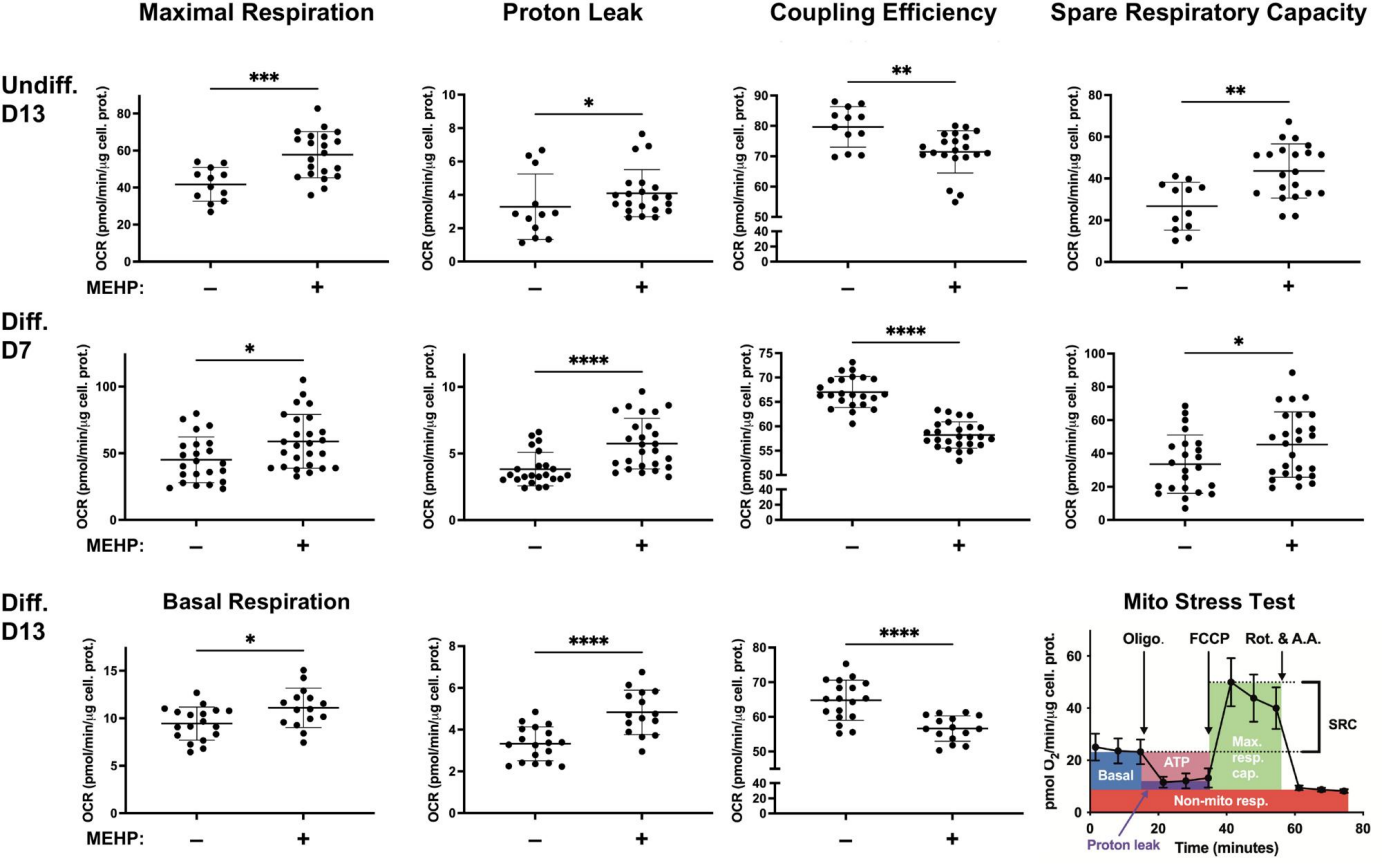
MEHP alters HepaRG mitochondrial bioenergetics. Undifferentiated- (Undiff.) and differentiated-derived (Diff.) HepaRG bioenergetic parameters were significantly impacted following 7 and 13 d (D7 and D13, respectively) of MEHP treatment. Bottom right, an illustration of the Seahorse Cell Mito Stress Test. Oligomycin (Oligo.); rotenone (Rot.); antimycin A (A.A.); basal respiration (Basal); ATP-linked respiration (ATP); non-mitochondrial respiration (Non-mito resp.); maximal respiratory capacity (Max. resp. cap.); spare respiratory capacity (SRC). The experiments were repeated at least 3 times on different days using different cell passages (≥12 total replicates). Statistical significance was determined using Student’s *t*-tests (parametric data) or Mann-Whitney U (nonparametric) tests. ****P ≤ 0.0001; ***P ≤ 0.001; **P ≤ 0.01; *P ≤ 0.05.

After 13 d, undifferentiated HepaRG had 10% less coupling efficiency, 1.6-fold more spare respiratory capacity, 1.4-fold increased maximal respiratory capacity, and 1.2-fold increased proton leak (Fig. 3 and Supplementary Fig. S56).

Reduced coupling efficiency and increased proton leak were similarly observed in differentiated HepaRG cultures. Following 7 d of MEHP treatment, differentiated-derived cultures exhibited a 13% decrease in coupling efficiency and 1.5-, 1.4-, and 1.3-fold increases in proton leak, spare respiratory capacity, and maximal respiratory capacity, respectively (Fig. 3). After 13 d of treatment, the differentiated-derived cultures had a 13% reduction in coupling efficiency, a 1.5-fold increase in proton leak, and a 1.2-fold increase in basal respiration. These results indicate that prolonged MEHP treatment is associated with altered mitochondrial bioenergetic function, including changes in parameters linked to OXPHOS activity, in both undifferentiated and differentiated HepaRG cell cultures. While interaction of MEHP with the MIM represents one plausible mechanism, the observed changes in bioenergetic parameters (including increases in maximal and spare respiratory capacities) may also reflect adaptive or compensatory responses, or alterations in electron transport chain complex expression or assembly.

### MEHP does not substantially alter mtDNA copy number at later time points

Because oxidative stress can promote mtDNA damage and degradation (Shokolenko et al. 2009), we examined whether MEHP altered mtDNA copy number or heteroplasmy. Although mtDNA repair of oxidative lesions is robust (Van Houten et al. 2018), damage can persist under sustained stress (Furda et al. 2012), potentially compromising mtDNA integrity. We therefore hypothesize that treatment with MEHP, which induces oxidative stress (Wang et al. 2012), may alter mtDNA copy number and affect mtDNA integrity, potentially manifesting as mtDNA variants or shifts in heteroplasmy. We treated cells with MEHP and measured mtDNA copy number (as a proxy for possible mtDNA degradation), as well as variants that arose and heteroplasmy, using a sensitive sequencing-based assay to determine whether MEHP can drive heritable changes in the mitochondrial genome.

MEHP treatment did not substantially alter mtDNA copy number in surviving HepaRG cultures, whether undifferentiated or differentiated, at 6 d or later (Fig. S57). A modest 9% decrease was observed in undifferentiated cells at day 6, whereas differentiated-derived cells showed a comparable 9% increase at the same time point.

### Minor shifts in low-level mtDNA heteroplasmy occur following MEHP treatment

To determine whether MEHP treatment leads to mutations or changes in mtDNA heteroplasmy levels, we performed mtDNA-specific next-generation sequencing (NGS) using whole-cell extracted (WCE) DNA samples from MEHP-treated undifferentiated and differentiated-derived HepaRG cells. mtDNA sequencing was performed at days 0, 3, and 12 of MEHP treatment in both cell types, with an additional time point at day 26 in differentiated cultures.

Although no significant mutagenic effects were observed, subtle changes in low-level heteroplasmy were detected in a small number of rare variants (Table 1). Two mtDNA variants (*MT-ND4* m.11940T>C, p. L394P, and *MT-ND5* m.12425dup, p. N30Kfs*29) showed modest, consistent decreases in heteroplasmy following MEHP treatment, most prominently in an undifferentiated passage 8 culture at day 12. These variants are rare in population databases (Lott et al. 2013; Bolze et al. 2019; Chen et al. 2024a) and were present at low baseline heteroplasmy (<6%). The observed downward trends in the percent heteroplasmy of these variants could reflect negative selection, that is, the removal of a deleterious heteroplasmic variant under MEHP-induced stress.

**Table 1.** Undifferentiated and differentiated-derived HepaRG percent mtDNA heteroplasmy levels following 0, 3, 12, or 26 d of MEHP treatment.

	Undiff. P8		Diff. P8		Undiff./Diff. P9^a^			Undiff. P10		Diff. P10		
Variant	D0	D12	D0	D3	Undiff. D0	Undiff. D3	Diff. D3	D0	D3	D0	D12	D26
m.3091G>A^b^	0.8	1.4	1.6	1.8	1.0	1.4	1.9	0.9	1.6	1.5	1.9	2.2
m.11940T>C^c^	5.4	2.4	2.0	1.9	2.6	2.1	1.6	2.7	1.6	2.0	1.8	1.4
m.12425dup^c^	1.5	0.9	1.2	0.9	1.5	1.0	1.2	1.7	1.4	2.1	1.7	1.4
m.14443C>A^b^	1.0	1.5	1.6	1.6	1.1	1.4	1.6	1.1	1.4	1.1	1.3	1.5

Levels of mtDNA heteroplasmy were determined by next-generation sequencing targeting mtDNA. D0, 3, 12, and 26 are samples that were sequenced following 0, 3, 12, and 26 d of MEHP treatment, respectively. Undiff. and Diff. are samples derived from undifferentiated and differentiated-derived HepaRG cultures.

a Undiff. P9 D0 serves as the baseline for both Undiff. P9 D3 and Diff. P9 D3 MEHP-treated samples. Similar trends in variant heteroplasmy, either increasing or decreasing, are observed for P8 and P10 when comparing Undiff D0 baselines to Diff. later time points.

b Samples with heteroplasmy increasing (or in the case of Diff. P8 m.14443C>A, unchanging) relative to D0.

c Samples with heteroplasmy decreasing relative to D0.

In contrast, a rare 16S rDNA m.3091G>A variant and a somewhat rare *MT-ND6* m.14443C>A missense variant (p.E77D) showed slight increases in heteroplasmy over time, reaching 2.2% and 1.6%, respectively.

Because all observed changes occurred at very low heteroplasmy levels, assay variability cannot be excluded as a contributing factor. Taken together, the mtDNA NGS results indicate that MEHP treatment under the conditions tested does not induce substantial mtDNA mutagenesis or pronounced heteroplasmy shifts but is associated with only minor variation in low-level mtDNA variants in HepaRG cultures. This lack of substantial mtDNA mutagenesis over the present experimental timeframe is consistent with prior studies showing that oxidative stress is not a major driver of mtDNA mutation in *Drosophila melanogaster* and that mtDNA mutation frequency does not increase in mice exposed to mutagens such as *N*-ethyl-*N*-nitrosourea and benzo[a]pyrene (Itsara et al. 2014; Valente et al. 2016).

### MEHP alters the growth and survival of HepaRG at early timepoints

**Fig. 4.**
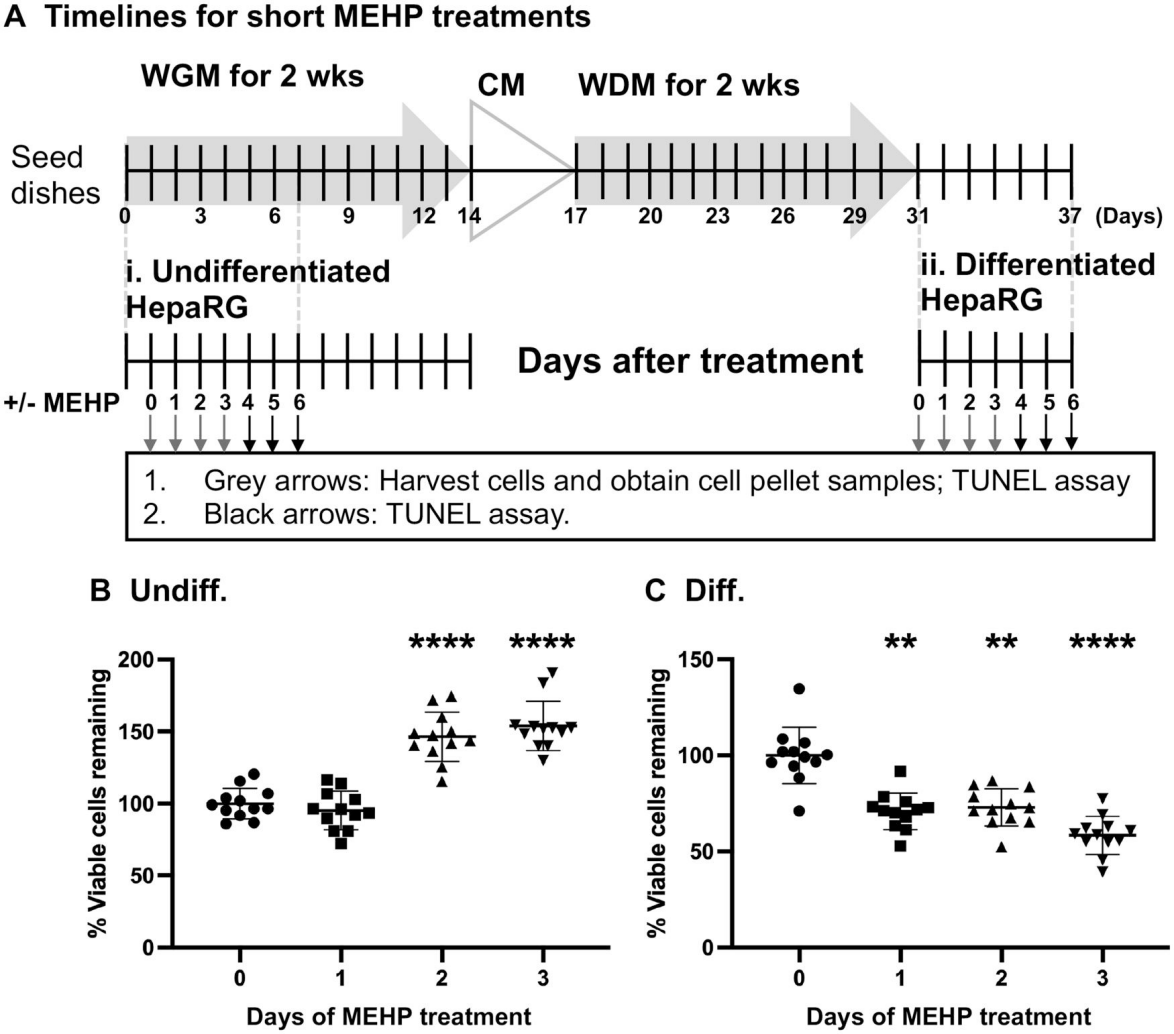
Differentiated-derived HepaRG cellular viability is significantly reduced after 3 d of MEHP treatment. (A) Timelines for the undifferentiated and differentiated-derived HepaRG short MEHP treatment experiments. Relative viable cell counts of (B) undifferentiated- (Undiff.) and C. differentiated-derived (Diff.) HepaRG cells following 0, 1, 2, and 3 d of MEHP treatment. The number of viable cells remaining on the cell culture dishes was determined using the trypan blue exclusion method. In each set of experiments, day 0 counts were set to 100 (%), and the relative percent viable cells remaining in the tissue culture dishes were calculated. Data are presented as mean values ± SD, *n* = 12 (quadruplicate from 3 independent experiments using different cell passages). Statistical significance was determined by one-way ANOVA. ****P ≤ 0.0001; **P ≤ 0.01.

A human volunteer study using contamination-controlled deuterated (D4)-DEHP (0.64 mg/kg) detected serum D4-MEHP concentrations of 17.5 µM at 2 h and 0.5 µM at 8.3 h post-dose with elimination half-lives of 2 and 5 h across 2 excretion phases (Koch et al. 2004). Given the rapid in vivo metabolism and clearance of MEHP, we evaluated early cellular responses to MEHP treatment in vitro. HepaRG cells were harvested following 0, 1, 2, and 3 d of treatment (Fig. 4A).

Viability was assessed in both undifferentiated and differentiated-derived HepaRG cultures at each time point. Previously, we demonstrated that the doubling time of undifferentiated HepaRG cells is 44 ± 4 h (Young et al. 2021); therefore, after about 2 d, the undifferentiated culture typically doubles in size. Consistent with continued proliferation, the undifferentiated cells treated with MEHP for a shorter period continued to grow, and following a 2-d treatment were ∼1.5-fold higher than the day 0 controls (146 ± 17% vs. 100 ± 11%) (Fig. 4B). However, this increase did not reach the expected 2-fold expansion observed under control conditions, and cell numbers remained at ∼1.5-fold after 3 d of exposure (154 ± 17%).

Compared with day 0 samples set at 100%, the number of differentiated-derived HepaRG cells was significantly reduced by 29% (71 ± 10%), 27% (73 ± 10%), and 42% (58 ± 10%) following 1, 2, and 3 d of MEHP treatment, respectively (Fig. 4C).

### Undifferentiated HepaRG short MEHP treatment causes mtDNA depletion

**Fig. 5.**
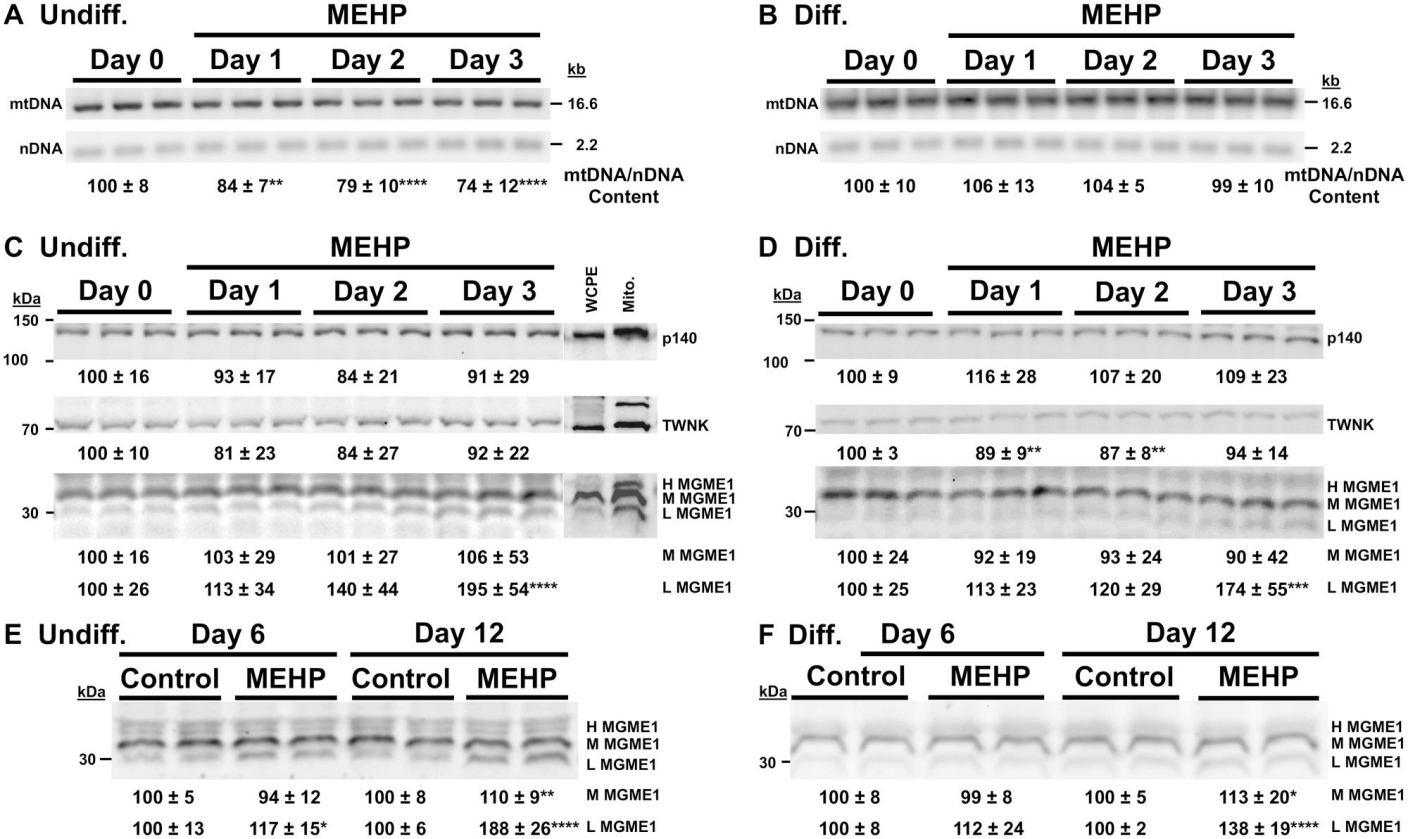
Short MEHP treatments decrease mtDNA content in undifferentiated HepaRG cells and induce the expression of a low-molecular-weight MGME1 isoform in both culture states. (A) Undifferentiated- (Undiff.) and (B) Differentiated-derived (Diff.) HepaRG cultures were treated with MEHP for 0, 1, 2, and 3 d, after which whole-cell extract (WCE) DNA was isolated. mtDNA content was analyzed by Southern blot and nonradioactive probe hybridization. The blots were simultaneously hybridized with DIG-labeled probes for mtDNA (upper panels) and 18S nuclear DNA (nDNA, lower panels). Representative blots for each culture state are shown. mtDNA signals were normalized to nDNA, with day 0 controls set to 100%. Quantified values represent mean ± SD from 3 independent biological replicates (different passages), each analyzed in technical triplicate (*n* = 9 total lanes per time point). (C) Undiff. and (D) Diff. show representative western blots for p140, TWNK, and MGME1 expression following 3 d of MEHP exposure. SJCRH30 mitochondrial extract (Mito) and whole-cell protein extract (WCPE) are included to demonstrate mitochondrial enrichment. Chemiluminescent band areas were normalized to total protein (TCE stain), with day 0 controls set to 100%. Under a representative blot, quantified data are presented as mean ± SD from 3 independent biological replicates (different passages), comprising ≥8 total lanes across 3 blots. (E) Undiff. and (F) Diff. show representative western blots for MGME1 expression following 6 and 12 d of MEHP exposures. Band intensities were normalized to total protein (TCE stain), with mock-treated negative controls set to 100%. For each culture state, the experiment was repeated 3 times on different days, using different cell passages (*n* = 12 lanes across 6 blots; 2 blots per experiment). *P ≤ 0.05, **P ≤ 0.01, ***P ≤ 0.001, ****P ≤ 0.0001.

As many of the undifferentiated and differentiated-derived cells died following 6 or more days of MEHP treatment (Fig. 2), and because slight fluctuations in low levels of heteroplasmy as early as 3 d following MEHP treatment were observed (Table 1), we investigated whether analyzing samples from earlier MEHP treatments would reveal more significant issues with mtDNA maintenance that could have been missed in the experiments where cells died on or before day 6. The mtDNA copy number in both culture types treated with MEHP for 1, 2, and 3 d was examined using our Southern blot method (Wheeler et al. 2019). mtDNA levels in each lane of the blot were determined using a mtDNA-specific probe signal relative to its matched nDNA-specific probe signal. The day 0 baseline mtDNA signal was set to 100%. Indeed, undifferentiated HepaRG cells treated with MEHP for 1, 2, and 3 d showed significant reductions of 16,% 21%, and 26% in mtDNA relative to the baseline, indicating MEHP-induced mtDNA depletion at early time points (Fig. 5A).

A similar approach was taken for differentiated-derived HepaRG cells, and the day 0 normalized mtDNA signal was set to 100%. In contrast to undifferentiated cells, the differentiated-derived relative mtDNA contents on days 1, 2, and 3 post-MEHP treatment were not significantly changed compared with day 0 controls (Fig. 5B).

### MEHP increases the expression of L MGME1 in both cell types at early time points

Because undifferentiated HepaRG cells experienced mtDNA depletion as early as 1 d following MEHP treatment, but differentiated-derived HepaRG did not (Fig. 5A and B), we wondered if changes in the expression of key mtDNA replication factors in the presence of MEHP could contribute to these culture-type differences. mtDNA replication requires the coordinated action of the DNA polymerase γ catalytic subunit (Pol γ p140), the TWNK helicase, and the mitochondrial genome maintenance exonuclease 1 (MGME1), which processes 5′ DNA flaps generated during mtDNA replication (Uhler et al. 2016; Falkenberg and Gustafsson 2020).

Levels of p140, TWNK, and MGME1 were analyzed and quantified by western blot. While slightly reduced levels of differentiated-derived HepaRG TWNK were observed on days 1 and 2 of MEHP treatment (11% and 13% reduced, respectively), in general, both culture types maintained ≥84% of the p140 (at most 116%) and ≥81% TWNK (at most 94%) over the 3 d of treatment when compared with the 100% day 0 baseline (Fig. 5C and D).

Human MGME1 is expressed as multiple protein isoforms derived from distinct transcript variants. Four MGME1 precursor isoforms are annotated in the NCBI protein database, ranging from 252 to 359 amino acids in length, all of which are predicted to contain an N-terminal mitochondrial targeting sequence (MTS) that is cleaved upon import (Claros and Vincens 1996). Following mitochondrial processing, isoforms 1 (39 kDa), 2 (37 kDa), and 4 (28 kDa) are predicted to generate catalytically active nucleases containing conserved PD-(D/E)XK and RecB-type nuclease motifs. In contrast, isoform 3 (26 kDa) lacks these motifs and is not predicted to possess exonuclease activity. Isoform 2 has been well characterized structurally and biochemically as a RecB-type exonuclease that cleaves ssDNA, processes DNA flap substrates, and interacts with p140 (Kornblum et al. 2013; Nicholls et al. 2014; Yang et al. 2018). While isoform 4 maintains the MTS, the PD-(D/E)XK motifs, and the RecB-type motifs, it lacks the residues 91 to 170 of isoform 2, which are also present in isoforms 1 and 3. In the isoform 2 crystal structure, α2 (143 to 160) and α3 (162 to 187) form an arch that ssDNA resides under, suggesting this region could be critical for binding ssDNA/5′-flaps (Yang et al. 2018).

Using western blotting and immunodetection, we observed 3 MGME1 immunoreactive bands ranging from <40 to ∼30 kDa, which were more abundant in a mitochondrial extract than in the matched whole-cell protein extract (WCPE). These bands are designated High (H), Mid (M), and Low (L) molecular weight MGME1, as shown in Fig. 5C, and we suspect they correspond to isoforms 1, 2, and 4, respectively. The M MGME1/isoform 2 bands identified in the HepaRG and SJCRH30 WCPE samples, and the SJCRH30 mitochondrial extract, were of strong intensities, which agrees with another study that found that the M MGME1 band was the most significant band on a blot containing control fibroblast protein lysate (Kornblum et al. 2013). The H MGME1 detected in the WCPEs was not quantified due to an inability to resolve it from other fuzzy band(s), which likely represent unprocessed precursor MGME1 of higher molecular weight (Fig. 5C and D).

Notably, MEHP treatment selectively increased the abundance of the L MGME1 isoform in both culture types. In undifferentiated HepaRG cells, L MGME1 levels increased approximately 2-fold after 3 d of MEHP treatment relative to day-0 controls (Fig. 5C). Differentiated-derived cells exhibited a similar, though slightly smaller, increase (∼1.7-fold) at the same time point (Fig. 5D). In contrast, M MGME1 levels remained essentially unchanged at the early time-point MEHP treatments.

At later time points (6 to 12 d), Pol γ p140 and TWNK levels remained comparable with controls in both culture types, Fig. S58. M MGME1 levels also remained near baseline, as shown in Fig. 5E and F. In contrast, L MGME1 expression remained significantly elevated following 12 d of MEHP treatment, with 1.9-fold and 1.4-fold increases observed in undifferentiated and differentiated-derived cultures, respectively (Fig. 5E and F). A modest but significant increase in L MGME1 was also observed in undifferentiated cells at day 6.

Collectively, these results demonstrate that MEHP induces upregulation of a low-molecular-weight MGME1 isoform in both proliferating and differentiated HepaRG cells, while leaving other core components of the mtDNA replisome largely unaffected.

### MEHP alters early PINK1 processing in a culture-dependent manner

The reduction in undifferentiated HepaRG mtDNA content at the early time point MEHP treatments (Fig. 5A) could indicate degradation of MEHP-induced mtDNA damage. Previously, we demonstrated that differentiated HepaRG cells harbor 7,400 mtDNA per cell, whereas undifferentiated HepaRG cells harbor 2,600 mtDNA per cell (Young et al. 2021). Therefore, if a similar absolute number of mtDNA molecules were degraded in both culture types after 1 d of MEHP treatment (16% of undifferentiated mtDNA or 416 mtDNA genomes), then 6,984 molecules or 94% of the starting population of differentiated mtDNA genomes would remain undigested. Our relative mtDNA copy number method may not be sufficiently sensitive to detect a 6% relative decrease.

If early time point MEHP treatments are linked to mtDNA damage followed by mtDNA degradation, that raises a second question: why does the mtDNA copy number appear restored in undifferentiated cells at later time points (Fig. S57A)? One possibility is that mitophagy could remove damaged mitochondria harboring mtDNA damage, thereby allowing healthy mitochondria to be repopulated with undamaged genomes. Mitophagy has been proposed to play a selective role in the turnover of dysfunctional mitochondria and the maintenance of mtDNA (Kim et al. 2007; Kim and Lemasters 2011; Kurihara et al. 2012; Bess et al. 2013; Babbar et al. 2020). Therefore, western blotting was used to examine the expression of the key autophagy factors PINK1 and p62 to determine whether they play a role in responding to MEHP-induced mitochondrial damage.

Under basal conditions, full-length PINK1 (FL-PINK1, 63 kDa) is imported into mitochondria and sequentially processed by the mitochondrial processing peptidase (MPP) and the presenilin-associated rhomboid-like protease (PARL) to generate intermediate-length PINK1 (IL-PINK1, 56 kDa) and cleaved PINK1 (c-PINK1, 52 kDa), respectively (Deas et al. 2011; Greene et al. 2012; Sekine and Youle 2018). The mature c-PINK1 is preferentially polyubiquitinated and degraded by the proteasome (Liu et al. 2017b).

**Fig. 6.**
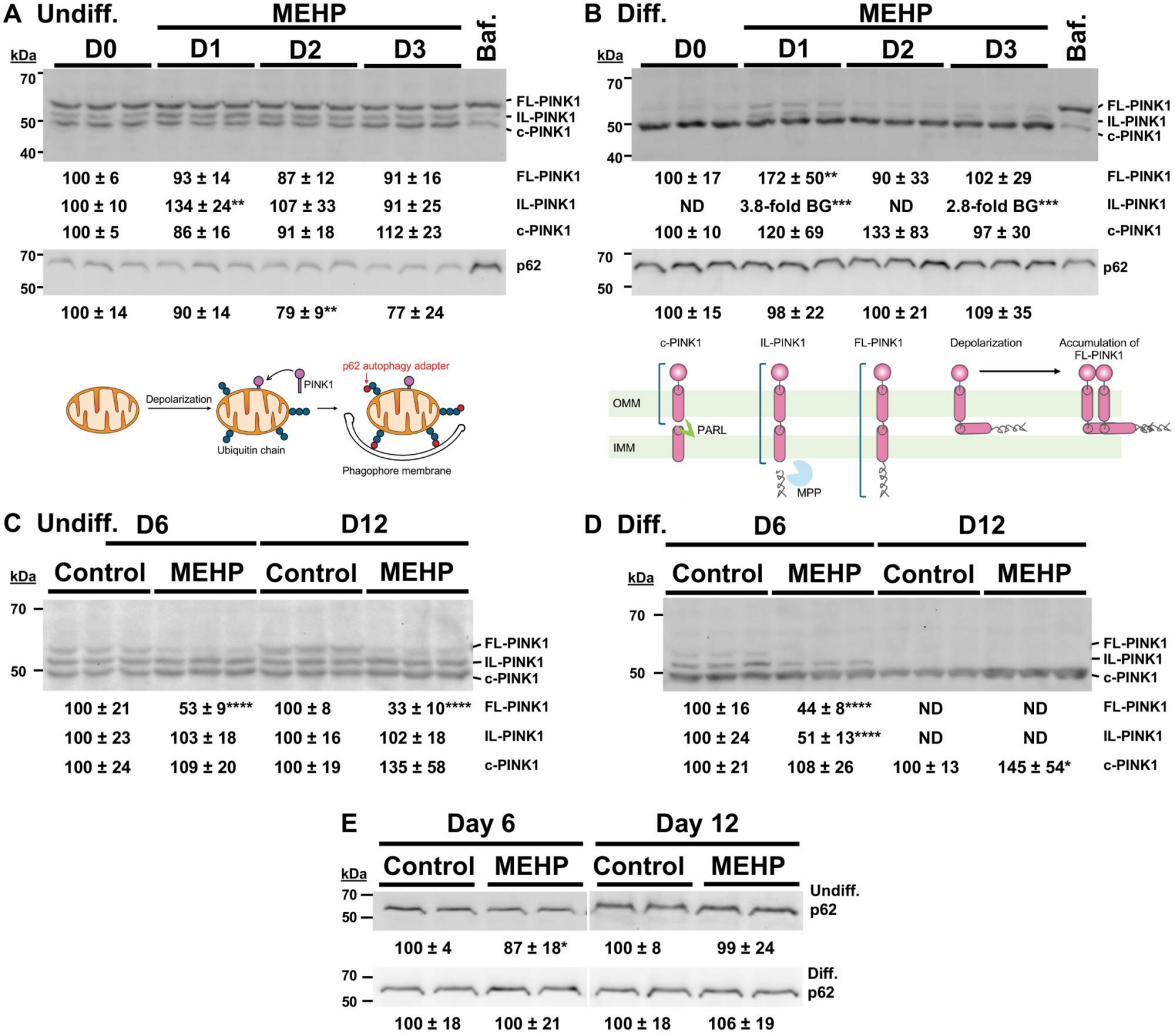
MEHP alters the expression of early-time-point p62 in undifferentiated cells and FL-PINK1 in differentiated-derived cells. (A) and (B) are representative western blots showing the expression of full-length (FL; uncleaved), intermediate length (IL; cleaved by MPP in the mitochondrial matrix), cleaved (c; cleaved by PARL in the IMM) PINK1 and p62 in (A) Undiff. and (B) Diff. HepaRG following 0, 1, 2, and 3 d of MEHP treatment. Chemiluminescent band areas were normalized to total protein (TCE stain), with day 0 controls set to 100%. Quantified data are presented as mean ± SD from 3 independent biological replicates (different passages), comprising n ≥ 8 total lanes across 3 blots. As a control, Undiff. HepaRG cells were treated with 100 µM bafilomycin A1 for 24 h to increase lysosomal pH and inhibit autophagosome-lysosome fusion, leading to increased p62 and FL-PINK1 as previously reported (Allen et al. 2013; Gomez-Sanchez et al. 2014). (C) Undiff. and (D) Diff. HepaRG PINK1 isoforms and (E) Undiff. and Diff. p62 expression following 6 and 12 d of MEHP treatment. Chemiluminescent band areas were normalized to total protein (TCE stain), with mock-treated negative controls set to 100%. Quantified data are presented as mean ± SD from 3 independent biological replicates (different passages), comprising *n* = 9 total lanes across 3 blots for PINK1; and *n* = 12 lanes from 6 blots for p62, 2 blots per experiment. Primary antibodies specific for PINK1 and p62 were used. *P ≤ 0.05, **P ≤ 0.01, ***P ≤ 0.001, ****P ≤ 0.0001.

In undifferentiated HepaRG, FL-PINK1 and c-PINK1 levels did not significantly change following 1, 2, or 3 d of MEHP treatment relative to day 0 baselines (Fig. 6A). IL-PINK1 increased modestly (1.3-fold) following 1 d of treatment, but returned to baseline levels by day 2, suggesting a transient alteration in PINK1 processing without sustained disruption of proteasomal turnover.

In contrast, differentiated-derived HepaRG cells showed distinct PINK1 expression patterns. Baseline day 0 FL-PINK1 and IL-PINK1 levels were low or undetectable, while c-PINK1 levels were abundant and remained unchanged during the 3-d treatment period (Fig. 6B). Following 1 d of MEHP treatment, FL-PINK1 increased 1.7-fold, and IL-PINK1 increased 3.8-fold relative to baseline, with IL-PINK1 also being elevated (2.8-fold) at day 3. These changes are consistent with impaired PINK1 processing and early accumulation of FL-PINK1 at the mitochondrial surface, indicative of mitophagy initiation.

### MEHP reduces p62 levels at early time points in undifferentiated HepaRG

To determine whether MEHP treatment alters autophagy-related signaling, we examined p62, an autophagy adapter protein. Decreased p62 levels are commonly associated with autophagy activation (Bjorkoy et al. 2009; Schlafli et al. 2016). In bafilomycin A1-treated undifferentiated HepaRG control samples, abundant FL-PINK1 and substantial p62 accumulation were detected (Fig. 6A), consistent with inhibition of autophagic turnover.

In undifferentiated cells exposed to MEHP for 2 d, a significant 21% reduction in p62 expression was detected relative to baseline (Fig. 6A). This decrease is consistent with enhanced autophagic or mitophagic flux at early time points. In contrast, p62 levels in differentiated-derived HepaRG cells remained unchanged during early MEHP treatment (Fig. 6B). Notably, baseline p62 expression in differentiated-derived cells was higher than that observed in bafilomycin A1-treated undifferentiated controls, suggesting that differentiated-derived cultures maintain elevated p62 reserves (Fig. 6B).

### Prolonged MEHP treatment favors proteasomal PINK1 turnover over autophagy

At later time points, undifferentiated HepaRG cells exhibited decreased FL-PINK1 levels (47% at day 6 and 67% at day 12), while IL-PINK1 and c-PINK1 levels remained unchanged relative to matched vehicle controls (Fig. 6C). Except for a modest 13% decrease in p62 at day 6, p62 levels remained stable at later time points (Fig. 6E), suggesting that prolonged MEHP treatment does not substantially increase p62-dependent autophagy.

In differentiated-derived HepaRG cells, FL-PINK1 and IL-PINK1 levels decreased by 56% and 49%, respectively, after 6 d of MEHP treatment (Fig. 6D). Although FL-PINK1 and IL-PINK1 were not detected at day 12, c-PINK1 levels increased 1.5-fold following MEHP treatment (Fig. 6D). The buildup of c-PINK1 at later time points may indicate altered proteasomal turnover or compromised proteasome activity in differentiated HepaRG under prolonged MEHP treatment.

### MEHP rapidly increases apoptotic nuclei in both culture types

Early MEHP-induced mitochondrial damage could activate mitophagy, as suggested by p62 depletion in undifferentiated day 2-treated cultures and by the accumulation of FL-PINK1 in differentiated cells on day 1 of treatment (Fig. 6). Similarly, enhanced L MGME1 expression was observed in both culture types by day 3 (Fig. 5), suggesting that early mitochondrial stress induces expression of an mtDNA maintenance factor. However, the considerable loss of cellular viability in both culture types following 6 d of MEHP treatment (Fig. 2B and C) suggests that mitochondrial quality-control mechanisms are insufficient to resolve phthalate-induced mitochondrial damage at the later time points.

Because environmental toxicants and persistent mtDNA damage can trigger apoptosis (Santos et al. 2003; Johnson and Sarosiek 2024), we hypothesized that early MEHP-induced damage leads to apoptotic elimination of cells, contributing to the reduced undifferentiated cell growth and decreased differentiated cell numbers observed at early time points (Fig. 4), as well as the pronounced loss of viability at later time points (Fig. 2). Previous studies have similarly demonstrated that exposure to MEHP and DEHP induces apoptosis (Ban et al. 2014; Li et al. 2014; Johnson and Sarosiek 2024).

**Fig. 7.**
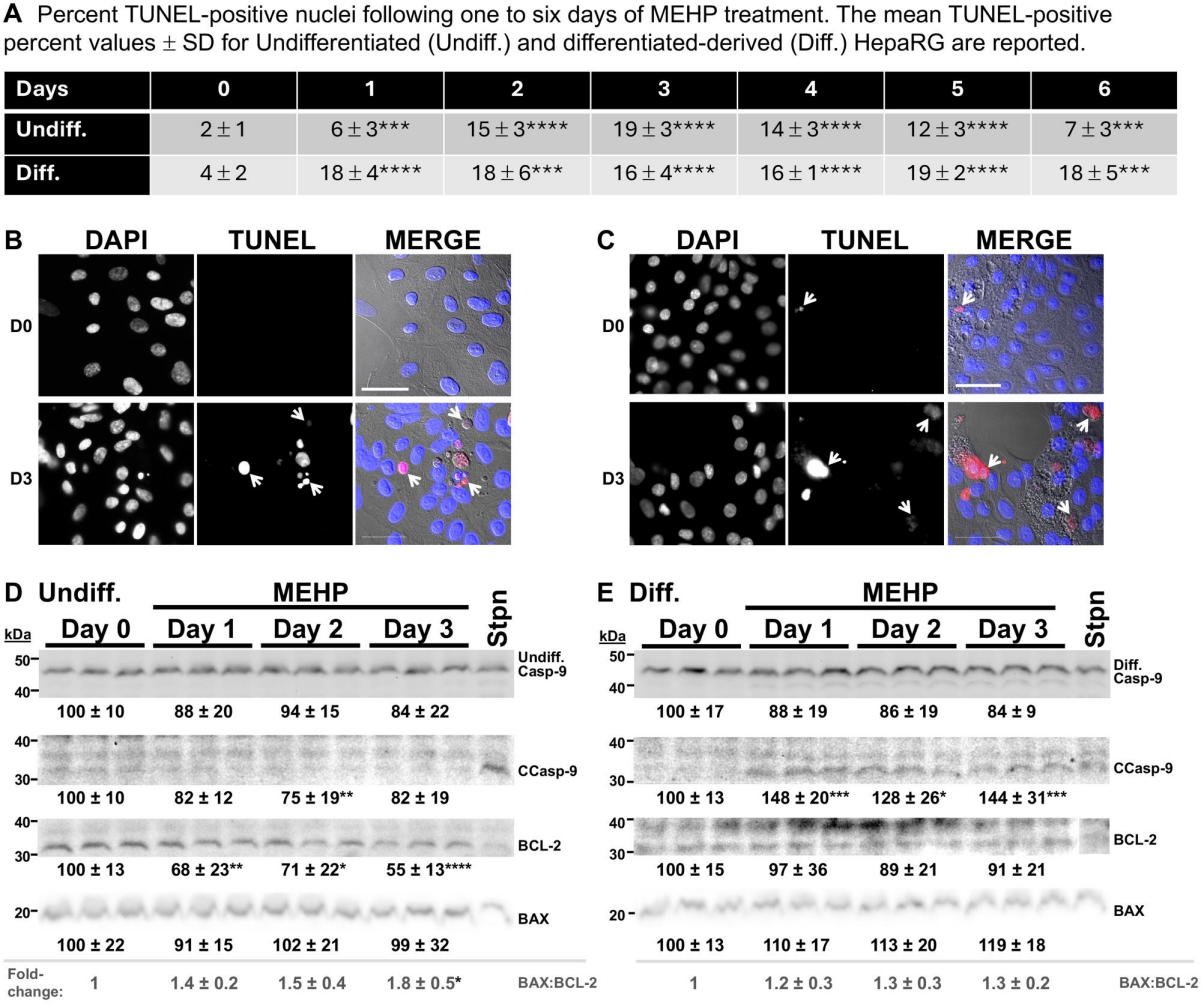
MEHP induces apoptosis in undifferentiated- and differentiated-derived HepaRG. A) Mean percent TUNEL-positive values ± SD following 6 d of MEHP treatment. Except for day 4 exposed differentiated-derived HepaRG samples, the number of nuclei was counted from 3 images for days 0, 1, 2, 3, 4, 5, and 6, and from 3 different experiments (*n* = 9 images; 3 images per replicate) across different passages. For day-4-treated differentiated-derived HepaRG samples, the experiment was performed twice (*n* = 6 images, 3 images per replicate). B) Undifferentiated- and C) Differentiated-derived HepaRG representative fluorescent images from days 0 (D0) and 3 (D3) of MEHP treatment followed by the TUNEL Apoptosis Assay. HepaRG cultures were dual-labeled with DAPI (blue nuclei) and AlexaFluor 594 (fragmented apoptotic DNA, red TUNEL). TUNEL-positive nuclei are emphasized with white arrows. For clarity, the DAPI and AlexaFluor (TUNEL) channels are shown separately in grayscale and together in color in the merged images. The scale bar length is 30 μm. D) and E) Representative western blots showing the expression of full-length caspase-9 (Casp-9), cleaved caspase-9 (CCasp-9), BCL-2, and BAX in (D) undifferentiated- (Undiff.) and (E) differentiated-derived (Diff.) HepaRG cultures following 0, 1, 2, and 3 d of MEHP treatment. Primary antibodies specific for Casp-9, CCasp-9, BAX, and BCL-2 were used. Band intensities were normalized to total protein (TCE stain), with day 0 controls set to 100%. Quantified data for each culture state are presented as mean ± SD from 3 independent biological replicates (different passages), comprising *n* ≥ 8 total lanes across 3 blots, 1 blot per experiment. As a control, Undiff. HepaRG cells were separately treated with 8 µM staurosporine (Stpn) for 9 h to induce apoptosis. Mean BAX-to-BCL-2 ratio values ± SD derived from the quantified data are reported below the representative BAX blots in gray text. *P ≤ 0.05, **P ≤ 0.01, ***P ≤ 0.001, ****P ≤ 0.0001.

To assess apoptosis, we used the Click-iT TUNEL assay following daily MEHP treatment for up to 6 d. In both undifferentiated and differentiated-derived HepaRG cultures, significant increases in TUNEL-positive nuclei were observed as early as day 1 compared with baseline day 0 controls (Fig. 7A).

In undifferentiated cultures, the percentage of apoptotic nuclei increased gradually through day 3, reaching a peak of 19%, and then declined but remained significantly elevated above the 2% baseline (Fig. 7A and B). Differentiated-derived cultures also exhibited significant apoptosis by day 1, with TUNEL-positive nuclei remaining relatively constant (16% to 19%) through days 1 to 6 compared with a 4% baseline (Fig. 7A and C). These findings indicate a rapid and sustained induction of apoptosis in both culture types following MEHP treatment.

### MEHP activates distinct apoptotic signaling pathways in undifferentiated and differentiated HepaRG

To examine molecular events initiating apoptosis, we analyzed caspase-9 (Casp-9), cleaved Casp-9 (CCasp-9), BAX, and BCL-2 expression following 0 to 3 d of MEHP treatment. Casp-9 is a key initiator of the intrinsic mitochondrial apoptosis pathway and undergoes cleavage during activation, generating 35- and 37-kDa subunits (Twiddy and Cain 2007).

Total Casp-9 levels did not significantly change in either culture type following MEHP treatment for 3 consecutive days compared with the day 0 control set at 100%, as shown in Fig. 7D and E. As expected, staurosporine-treated undifferentiated cells exhibited robust accumulation of the 35 kDa cleaved CCasp-9 subunit, confirming antibody specificity (Fig. 7D and E). In undifferentiated HepaRG cells, CCasp-9 levels remained essentially unchanged following MEHP treatment, except for a modest 25% decrease at day 2 (Fig. 7D). In contrast, differentiated-derived HepaRG showed consistent increases in CCasp-9 levels (1.3 to 1.5-fold) across days 1 to 3 of treatment (Fig. 7E), consistent with intrinsic apoptosis activation.

We next evaluated the expression of the pro-apoptotic protein BAX and the anti-apoptotic protein BCL-2, which together regulate mitochondrial outer membrane permeabilization (MOMP) (Ichim and Tait 2016). An elevated BAX-to-BCL-2 ratio can indicate induction of apoptosis (Fortuno et al. 1998; Perlman et al. 1999; Sp et al. 2021). BAX expression remained unchanged in both culture types following MEHP treatment (Fig. 7D and E). In undifferentiated HepaRG, however, BCL-2 levels were significantly reduced by 32%, 29%, and 45% on days 1, 2, and 3 of treatment, respectively (Fig. 7D). Differentiated-derived cultures did not show significant changes in BCL-2 expression (Fig. 7E).

As a result, undifferentiated HepaRG exhibited a significant 1.8-fold increase in the BAX-to-BCL-2 ratio by day 3 of MEHP treatment, whereas differentiated-derived cultures showed no substantial change in this ratio. Taken together, these results indicate that MEHP induces apoptosis in both culture types but engages distinct molecular mechanisms: differentiated HepaRG cells show caspase-9 activation, whereas undifferentiated HepaRG cells exhibit altered BCL-2-dependent regulation of MOMP.

### Evaluation of MEHP effects on mtDNA maintenance in an independent mitochondrial model

To determine whether the observed mitochondrial effects of MEHP were specific to the HepaRG system or reflected a broader mitochondrial response, we evaluated mtDNA copy number following MEHP treatment in an independent mitochondrial-competent model, mouse C2C12 myoblasts. C2C12 cultures provide a well-characterized system for studying mitochondrial metabolism and mtDNA regulation (Nicholls et al. 2010; Frangini et al. 2013; Kolesar et al. 2013). A previous study reported that 200 to 800 µM MEHP exposures in C2C12 myoblasts slowed proliferation, whereas 100 µM MEHP increased superoxide dismutase activity and ROS levels, and reduced the mitochondrial network (Chen et al. 2020).

**Fig. 8.**
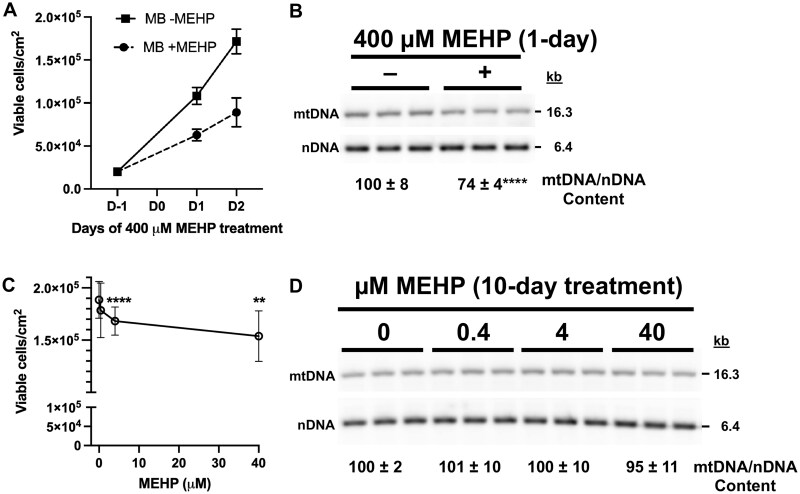
MEHP effects on C2C12 cell density and mtDNA copy number. A) C2C12 myoblast viability following 2 d of mock (–) and 400 μM MEHP (+) treatment. D-1 is the day of seeding in growth medium; cells were allowed to adhere overnight; D0 is the day of treatment initiation. B) C2C12 myoblast mtDNA is depleted following 1 d of 400 μM MEHP treatment. WCE DNA samples were subjected to agarose gel electrophoresis, and mtDNA content was analyzed using Southern blot with nonradioactive probe hybridization. The blots were simultaneously hybridized with mouse-specific DIG-labeled probes for mtDNA (upper panel) and 18S nDNA (lower panel). mtDNA signals were normalized to nDNA, with the mock-treated control set to 100%. C) C2C12 myoblast viability following 10-d treatment with 0, 0.4, 4, and 40 μM MEHP. D) C2C12 myoblast mtDNA copy number following 10 d of MEHP treatment. Following 10-d treatments with 0, 0.4, 4, and 40 μM MEHP, myoblast WCE DNA was isolated, and mtDNA content was analyzed as described in (B). mtDNA signals were normalized to nDNA, with the mock-treated control (0 μM MEHP) set to 100%. Viability and mtDNA quantification data are presented as mean ± SD from 3 independent biological replicates (different passages), each analyzed in ≥3 technical replicates (*n* ≥ 9 total measurements per treatment). Representative blots are shown in (B) and (D) **P ≤ 0.01, ****P ≤ 0.0001.

To determine whether MEHP treatment in C2C12 cells recapitulates the mtDNA-depletion observed in undifferentiated HepaRG cultures, we exposed C2C12 myoblasts to 400 µM MEHP. Consistent with reduced proliferation, after 1 and 2 d of treatment, myoblast density was 42% and 48% lower than that of mock-treated controls, respectively (Fig. 8A). Following 1 d of MEHP treatment, a 26% decrease in mtDNA copy number was observed, consistent with mtDNA depletion under high-concentration, short-duration treatment conditions (Fig. 8B).

Because in vitro assays compress exposure timelines, we next assessed whether lower concentrations applied over a longer duration would similarly affect mtDNA copy number. We therefore tested lower MEHP concentrations (40, 4, and 0.4 µM) over a prolonged treatment period. In contrast to the pronounced effects observed at 400 µM MEHP, 10-d treatments with 4 and 40 µM MEHP resulted in modest but statistically significant decreases in cell density of 11% and 18%, respectively, compared with mock-treated cells (Fig. 8C). Under these conditions, mtDNA copy number remained largely unchanged across treatments, with no substantial depletion observed, despite measurable effects on cell density, indicating that lower MEHP concentrations impair cell growth without detectably affecting mtDNA maintenance (Fig. 8D). Together, these findings indicate that MEHP reduces C2C12 cell density across concentrations, while mtDNA depletion is observed only under high-concentration treatment conditions.

## Discussion

Our study provides mechanistic insight into how high-concentration MEHP compromises mitochondrial integrity and viability in HepaRG cells, in a differentiation-dependent manner. MEHP is expected to partition into lipid-rich mitochondrial membranes due to its lipophilic nature. Physiologically based pharmacokinetic (PBPK) models incorporate adipose tissue compartments to account for the lipophilic distribution of phthalates (Sharma et al. 2018; Chen et al. 2024b), but likely reflect reversible tissue-plasma partitioning rather than true bioaccumulation, as DEHP and its metabolites undergo rapid turnover in vivo (Koch et al. 2003).

We hypothesized that bioenergetics and mtDNA maintenance could be altered by days-long MEHP treatment. We showed that prolonged MEHP treatments (6 to 12 d) decreased viability and perturbed bioenergetic parameters in both undifferentiated and differentiated cells. By day 12, differentiated HepaRG cells exhibited a 3-fold decrease in viability, and by day 13, greater changes in proton leak and coupling efficiency were observed (Figs 2 and 3). The MEHP-induced increase in proton leak and decrease in coupling efficiencies in both culture types are consistent with altered mitochondrial function (Hill et al. 2012).

Based on our previous estimate that mtDNA is depleted more rapidly in undifferentiated HepaRG exposed to ddC (Young et al. 2021), we investigated whether MEHP could upregulate an mtDNA-degradation pathway in undifferentiated cells, resulting in greater mtDNA depletion than in differentiated cells. In short treatment experiments (1 to 3 d), differentiated-derived cultures exhibited reduced cellular viability, whereas undifferentiated cells displayed early mtDNA depletion (Figs 4 and 5A). The 26% reduction in undifferentiated HepaRG mtDNA by day 3 is comparable with the 30% loss observed in our p140/*POLG* Y955C model of mitochondrial disease (Rahman et al. 2022). MGME1 is a key exonuclease involved in mtDNA maintenance and degradation and, to our knowledge, has not been linked to phthalates. Across both cell culture states, MEHP induced upregulation of the L MGME1 isoform by day 3 of treatment, suggesting enhanced nuclease activity and altered mtDNA maintenance (Fig. 5C–F).

Due to the multicopy nature of mtDNA, damaged molecules can be discarded and replaced by new genomes. In a proposed degradosome model, mtDNA harboring DSBs is rapidly cleared by a machinery comprising Pol γ p140, MGME1, and TWNK (Peeva et al. 2018). These data suggest MEHP perturbs mtDNA homeostasis via MGME1.

MGME1 is essential for mtDNA maintenance, and MEHP increases L MGME1. Given that MGME1 isoform 2 processes ssDNA 5′ to 3′ and removes DNA flaps, overexpression or misregulation of the L MGME1 isoform may exacerbate mtDNA loss. During short-term treatment with high-concentration MEHP, mtDNA depletion in undifferentiated cells occurred before overt cell death, indicating early involvement of genomic instability.

Modified autophagy factors were observed in both culture types exposed to MEHP, suggesting that autophagy is engaged. Undifferentiated p62 levels decreased on day 2 of treatment, while differentiated FL-PINK1 levels increased on day 1 of treatment (Fig. 6), suggesting culture-specific autophagy activation. A previous study using proliferating cells, separately treated with 200 μM MEHP, 5 μM CCCP, or MEHP followed by CCCP, showed that MEHP alone does not induce FL-PINK1 accumulation/stabilization at the MOM, consistent with observations in proliferating, undifferentiated HepaRG (Xu et al. 2021).

Consistent with a potential interaction of MEHP with the MIM and altered mitochondrial function, increased IL-PINK1 levels by day 1 of treatment in both HepaRG culture types suggest impaired activity of the MIM-localized PARL protease, which cleaves IL-PINK1 to generate c-PINK1 (Fig. 6A and B). Our findings indicate that MEHP engages the pathway in a differentiation-dependent manner and may couple it to mtDNA maintenance via MGME1 induction, thereby revealing an additional layer of mitochondrial vulnerability.

Consistent with the rapid induction of apoptosis by MEHP, both culture types exhibited TUNEL-positive nuclei as early as day 1 of treatment. Additionally, the day 3 undifferentiated BAX-to-BCL-2 ratio and the differentiated CCasp-9 levels from days 1 to 3 increased (Fig. 7). Taken together, these results support a model in which MEHP disrupts mitochondrial homeostasis and engages L MGME1, autophagy, and apoptotic pathways.

The present study was designed to identify mitochondrial pathways vulnerable to MEHP treatment rather than to establish human-relevant exposure thresholds. As a first step in risk assessment, we used 300 μM MEHP, based on our in vitro IC_50_ values, to determine that it poses a potential hazard to mitochondria by inducing mtDNA depletion in undifferentiated HepaRG cells and promoting autophagy and apoptosis in both undifferentiated and differentiated cultures. However, in vitro concentrations may substantially overestimate the amount of bioactive phthalate to which cells are exposed because MEHP adsorbs onto plasticware (e.g., polyethylene) and binds to proteins and lipids in serum-containing media (Deng et al. 2021; Hughes et al. 2021).

To place our findings in a broader toxicological context, we estimated the freely available MEHP concentration corresponding to the nominal in vitro dose, accounting for protein binding under serum-containing culture conditions (see *Simplified calculation to estimate the effect of serum protein binding on unbound MEHP in vitro*). We estimate a free MEHP concentration of ∼19.8 µM; however, this value should not be interpreted as directly equivalent to circulating plasma levels in humans, which have been reported to have an unbound fraction of ∼0.007 (Adachi et al. 2015). This estimate contextualizes in vitro treatment conditions rather than defining exposure thresholds.

Importantly, defining dose–response relationships at lower, environmentally relevant exposures is a distinct scientific objective that requires integrating PBPK modeling to guide in vitro-to-in vivo extrapolation (IVIVE). Such approaches will be essential in future studies to translate the mitochondrial mechanisms identified here to realistic exposure scenarios and to evaluate their contribution to phthalate-associated mitochondrial injury in vivo. Our HepaRG results should be interpreted to indicate mitochondrial pathways vulnerable to MEHP. Future studies incorporating primary human hepatocytes and non-parenchymal liver cells will be necessary to validate the physiological relevance of the pathways identified here.

To further evaluate MEHP effects on mtDNA maintenance, we used an independent, mitochondrial-competent C2C12 myoblast system. Although not liver-derived, C2C12 cells provide a model for mitochondrial biogenesis, oxidative metabolism, and mtDNA regulation (Frangini et al. 2013; Kolesar et al. 2013; Chen et al. 2020). A reduction in mtDNA copy number was observed following high-concentration MEHP treatment, but not at lower concentrations (Fig. 8B and D). Notably, 4 and 40 µM MEHP reduced cell density without detectable mtDNA depletion (Fig. 8C), indicating that early growth effects occur independently of mtDNA loss. These results are consistent with a report showing that a mixture of phthalate metabolites decreases antral follicle growth in mice (Meling et al. 2020). Importantly, the absence of mtDNA depletion at lower concentrations, despite reduced cell density, suggests that mtDNA instability is not the primary driver of early cellular responses to MEHP. Rather, these findings indicate that perturbations in cell growth can occur independently of mtDNA loss, and that mtDNA depletion may represent a later or higher-threshold event associated with more severe mitochondrial stress.

Mitochondrial dysfunction provides a mechanistically plausible link between phthalate exposure and many reported adverse health outcomes, including metabolic dysregulation, hepatic injury, and altered cell survival (Li et al. 2021; Yu et al. 2021; Aydemir et al. 2023; Kabekkodu et al. 2024; Sun et al. 2024; Gogola et al. 2025). Mitochondria regulate energy metabolism, redox balance, and apoptosis, and disruption of these processes has been implicated in phthalate-associated liver disease and insulin resistance. Notably, much of the phthalate literature emphasizes PPAR-dependent transcriptional mechanisms (Hurst and Waxman 2003; Fan et al. 2010; Mohammadi and Ashari 2021). In contrast, our data support a model in which high concentration MEHP induces mitochondrial dysfunction and apoptosis through PPAR-independent mechanisms that directly compromise mitochondrial integrity. The observed mitochondrial defects and apoptotic responses are consistent with bioenergetic failure and organelle-level stress, which can independently trigger cell death, irrespective of transcriptional activation of PPAR targets.

The present study extends previous observations on phthalate toxicity by simultaneously comparing proliferative and differentiated hepatocyte states and linking mtDNA perturbation to mitochondrial quality control. Our findings build upon a body of evidence implicating mitochondria in phthalate toxicity (Bell 1982; Melnick and Schiller 1982; Ban et al. 2014; Chen et al. 2020; Shi et al. 2020; Traore et al. 2021). Earlier studies have shown that MEHP elevates ROS levels, depolarizes mitochondrial membrane potential, impairs OXPHOS subunit expression, and induces apoptotic signaling in other models (Ban et al. 2014; Li et al. 2014; Chen et al. 2020; Johnson and Sarosiek 2024). Based on the HepaRG viability and mtDNA data at early time points, we suggest that differentiated hepatocytes are more sensitive to MEHP, whereas proliferating hepatocytes tolerate early MEHP-induced stress but exhibit mtDNA perturbations under higher-stress conditions. Undifferentiated HepaRG cells exhibit early mtDNA depletion, whereas differentiated cells are more immediately sensitive to loss of cellular viability. Both states show compromised bioenergetics, induction of a low-molecular-weight MGME1, activation of autophagy/mitophagy, and apoptosis. The combination of bioenergetic dysfunction, activation of quality-control pathways, and context-dependent mtDNA changes supports a model in which mitochondria are both targets and mediators of cellular stress. Within a risk assessment framework, the mitochondrial endpoints identified here represent early key events for potential future dose-response and IVIVE analyses.

## Supplementary Material

kfag049_Supplementary_Data

## Data Availability

All the data described herein is within this article. Reagents, plasmids, etc., are available upon request by contacting the corresponding author, M.J.Y.
