# Supplementary material for: High-concentration MEHP triggers mtDNA depletion in undifferentiated HepaRG and C2C12 cultures and disrupts mitochondrial homeostasis in both HepaRG culture states
Source: Toxicol Sci. 2026 Apr 29;209(5):kfag049. doi: 10.1093/toxsci/kfag049 (PMC13195206; doi:10.1093/toxsci/kfag049)
Supplement: kfag049_Supplementary_Data [file kfag049_supplementary_data.zip › HepaRG.MEHP.ms.04.13.2026.supp.MJY.v3.docx]

Supplementary Figure legends for **“High-concentration MEHP triggers mtDNA depletion in undifferentiated HepaRG and C2C12 cultures and disrupts mitochondrial homeostasis in both HepaRG culture states.**”

Md Mostafijur Rahman^1†^, Pabitra Khadka^1†^, Carolyn K. J. Young^1^, Jing Wang^3^, Elizabeth M. McCormick^4^, Marni J. Falk^4,5^, and Matthew J. Young^1,2^

^1^Department of Biomedical Sciences, Division of Biochemistry & Molecular Biology, Southern Illinois University School of Medicine, Carbondale, IL 62901

^2^Simmons Cancer Institute, Springfield, IL 62702

^3^Division of Genomic Diagnostics, Department of Pathology and Laboratory Medicine, University of Pennsylvania Perelman School of Medicine, The Children's Hospital of Philadelphia, PA 19104

^4^Mitochondrial Medicine Frontier Program, Division of Human Genetics, Department of Pediatrics, The Children’s Hospital of Philadelphia, Philadelphia, PA 19104

^5^Department of Pediatrics, University of Pennsylvania Perelman School of Medicine, Philadelphia, PA 19104

^†^These authors have contributed equally to this work

Correspondence to Matthew J. Young, [myoung84@siumed.edu](mailto:myoung84@siumed.edu) ([matthew.young@siu.edu](mailto:matthew.young@siu.edu))

Telephone number: (618) 453-6437

The Supplementary Figures (as .tif files) have been deposited in Dryad, Dataset DOI:

10.5061/dryad.c59zw3rpr.

**Supplementary Figures 1 to 55.** Supplementary western blot data showing TCE-stained protein on the blots before immunodetection. Supplementary Figures 1-36 and 37-52 are chemiluminescent and TCE-stained blots from short (day 0, 1, 2, and 3) and prolonged treatment (day 6 and 12) samples, respectively. Supplementary Figures 53-55 highlight chemiluminescent and TCE-stained blots containing mitochondrial enrichment of replisome factors (p140, TWNK, and MGME1). For all blots, a TCE-stained image was taken, with the signal in each lane representing the total protein level. After TCE staining, blots were cut horizontally at one or multiple positions based on the molecular weight (MW) of the target, and the sliced pieces were used for immunodetection with the corresponding antibody (Ab). For example, because the MWs of all MGME1 isoforms are below 40 kDa, the blot was cut into strip(s) at 40 kDa or slightly above to capture all the MGME1-associated signal in the slice. Similarly, incision sites for other protein blots were determined based on their MW, ensuring sufficient space above and below the desired band to quantify the signal properly. Incision sites are indicated in the image as black arrows. MW of the different bands in the MW marker are represented as numbers (in kDa) on the left side of the blots. The chemiluminescent signal of the desired band of each protein in a lane was normalized to the TCE-stained total protein signal of the same lane. For some chemiluminescent blots, an over-exposed (OE) slice containing the desired band was shown for clarity. All blots were performed as described above, unless otherwise noted in the individual file. WCPE, whole cell protein extract; Baf, Bafilomycin A1.

**Supplementary Figure 56. Undifferentiated and differentiated HepaRG MitoStress test profiles following MEHP treatment.** **A**. Undifferentiated HepaRG Mito Stress test oxygen consumption rates (OCRs, pmol O_2_/min/μg cellular protein) and extracellular acidification rates (ECARs, mpH/min/μg cellular protein) following 13 days of MEHP treatment. Differentiated HepaRG Mito Stress test profiles following **B.** 7 days and **C.** 13 days of MEHP treatment. Data are mean values ± SD; n ≥ 12, from at least three independent experiments using different preparations/passages of cells. Inverted triangles with dotted lines represent 300 µM MEHP treatment (+); open circles with solid lines represent vehicle control (−). Metabolic stressors were injected sequentially from Ports *a* (2 μM oligomycin), *b* (1 μM FCCP), and *c* (0.5 μM antimycin A + 0.5 μM rotenone). Olig., oligomycin; Rot., rotenone; A.A., antimycin A. OCRs were used to calculate the bioenergetic parameters reported in Figure 3.

**Supplement Figure 57. Surviving undifferentiated and differentiated-derived HepaRG cells maintain mtDNA upon prolonged MEHP treatment**. BamHI-digested whole-cell extracted DNA samples from **A.** Undifferentiated, and **B.** Differentiated-derived HepaRG were analyzed via Southern blot and nonradioactive probe hybridization. DNA samples from untreated (Control) and MEHP-treated undifferentiated (day 6 and 12 post-exposure) and differentiated (day 6, 12, and 26 post-exposure) HepaRG cells were extracted, followed by agarose gel electrophoresis and transfer to nitrocellulose membrane. The blots were simultaneously probed with the DIG-labeled 18S nuclear DNA probe (nDNA, lower panel) and the mtDNA-specific probe (mtDNA, upper panel). Bands were quantified using the open-source image-processing package Fiji, as described (Wheeler et al. 2019). A representative blot is shown. On each blot, and for each day, the average normalized band intensity values of control undifferentiated and differentiated-derived HepaRG mtDNA relative to nDNA were separately set to 100%, and the samples from the other days were compared with it. For undifferentiated and differentiated-derived HepaRG on days 6 and 12, quadruplicates for the first and third replicate experiments, and triplicates for the second replicate experiment were loaded on the gel (n=11 from three blots representing three different replicate experiments using different passages of cells, one blot per experiment). Data from day 26 differentiated-derived cells represent two experiments with different cell passages (n=8 from 4 blots; 2 blots per experiment).

**Supplement Figure 58. Prolonged MEHP treatment slightly increases p140 and TWNK levels in undifferentiated and differentiated-derived HepaRG cells**. Representative western blots of p140 and TWNK expression in control (day 6 and 12) and MEHP (days 6 and 12) treated **A.** Undifferentiated and **B.** Differentiated-derived HepaRG cells. Following the MEHP treatment experiments, whole-cell protein extracts (WCPEs) were prepared and analyzed by SDS-PAGE and western blot. Primary antibodies against p140 and TWNK were used to detect corresponding proteins on the blot. Chemiluminescent band areas were normalized to total protein (TCE stain), with mock-treated negative controls set to 100%. Quantified data are presented as mean ± SD from three independent biological replicates (different passages), comprising n ≥ 11 total lanes across six blots, 2 blots per experiment.
