# Supplementary material for: High-concentration MEHP triggers mtDNA depletion in undifferentiated HepaRG and C2C12 cultures and disrupts mitochondrial homeostasis in both HepaRG culture states
Source: Toxicol Sci. 2026 Apr 29;209(5):kfag049. doi: 10.1093/toxsci/kfag049 (PMC13195206; doi:10.1093/toxsci/kfag049)
Supplement: kfag049_Supplementary_Data [file kfag049_supplementary_data.zip › Supplementary.Figures.1.to.58.03.31.2026.pdf]

p140_TWNK_MGME1_HepaRG_Diff_Day_0123_n1

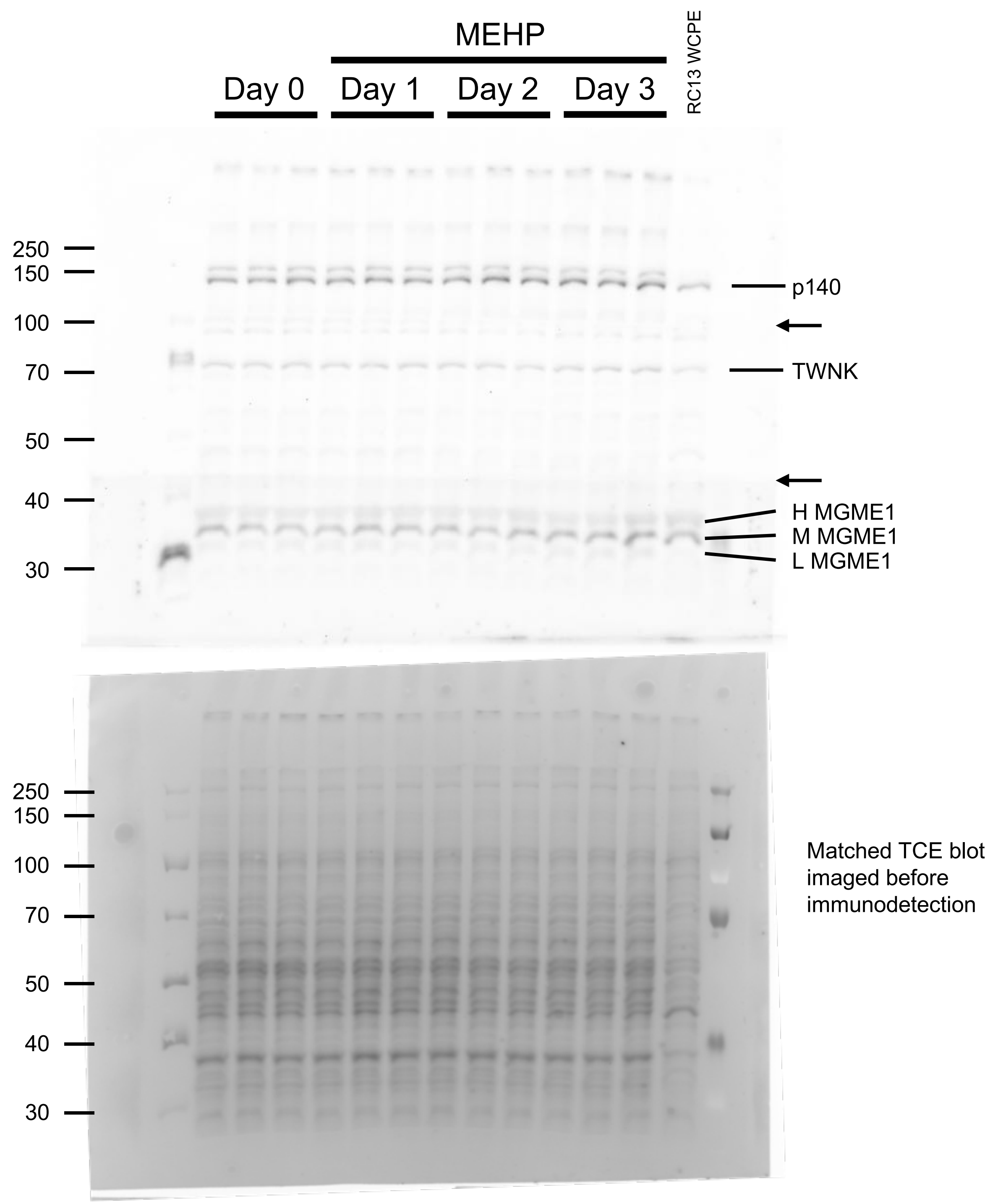

The top and bottom images represent Chemiluminescent and TCE-stained blots, respectively. Blots were run and detected as described in the Methods section. Molecular weight standards are indicated on the left-hand side of the blots in kDa. The black arrows indicate the sites of incision after TCE staining. WCPE, whole cell protein extract.

Supplementary Figure 1

p140_TWNK_MGME1_HepaRG_Diff_Day_0123_n2

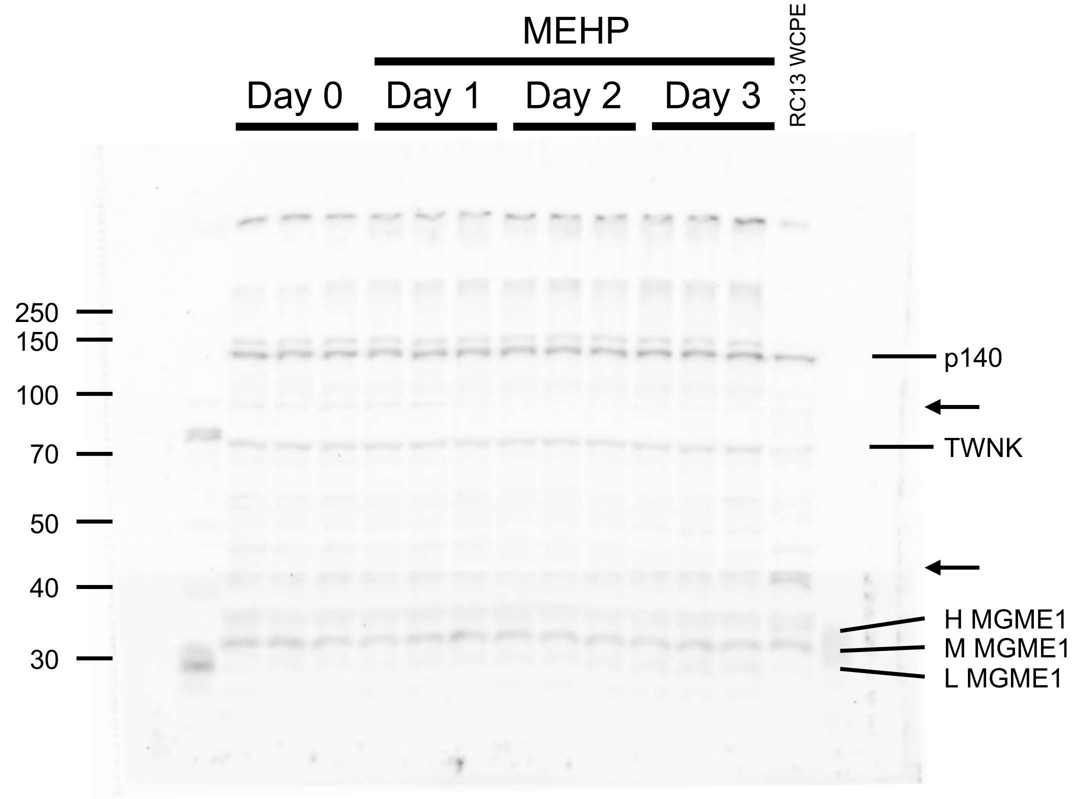

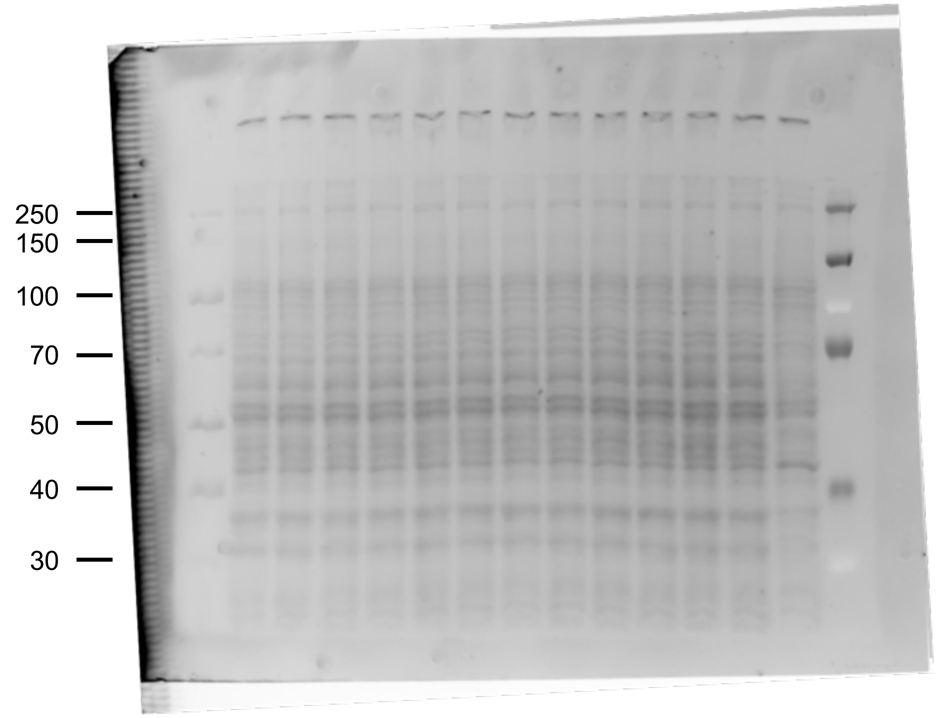

Matched TCE blot imaged before immunodetection

Supplementary Figure 2

p140_TWNK_MGME1_HepaRG_Diff_Day_0123_n3

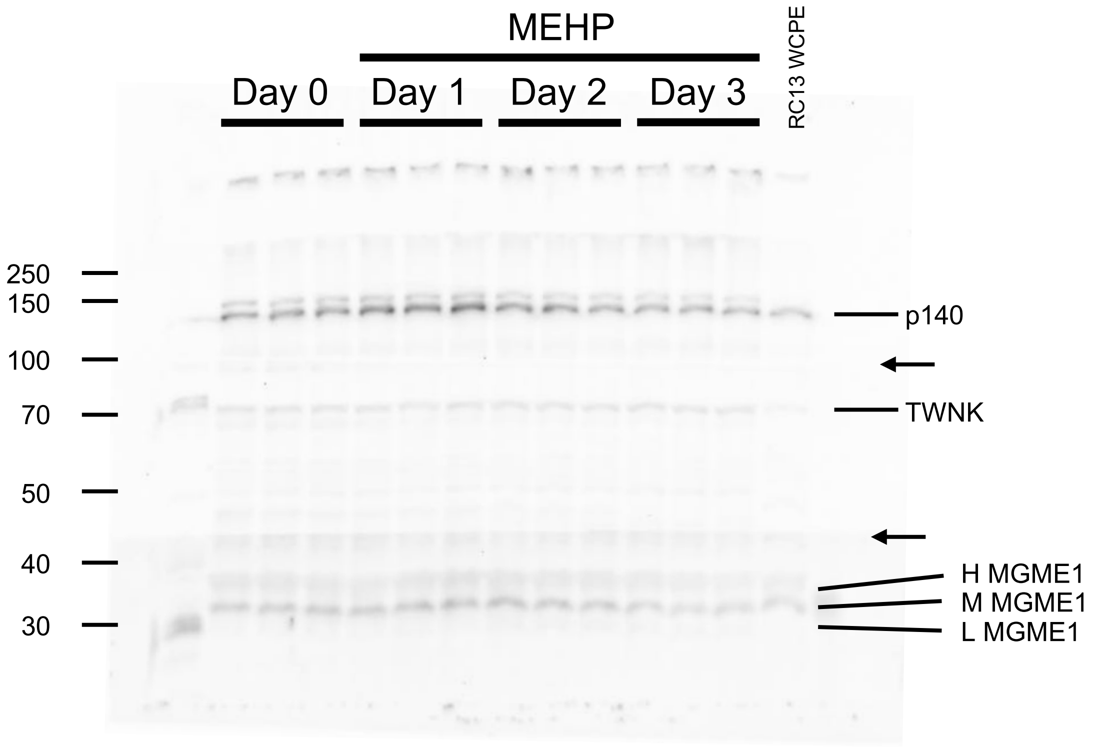

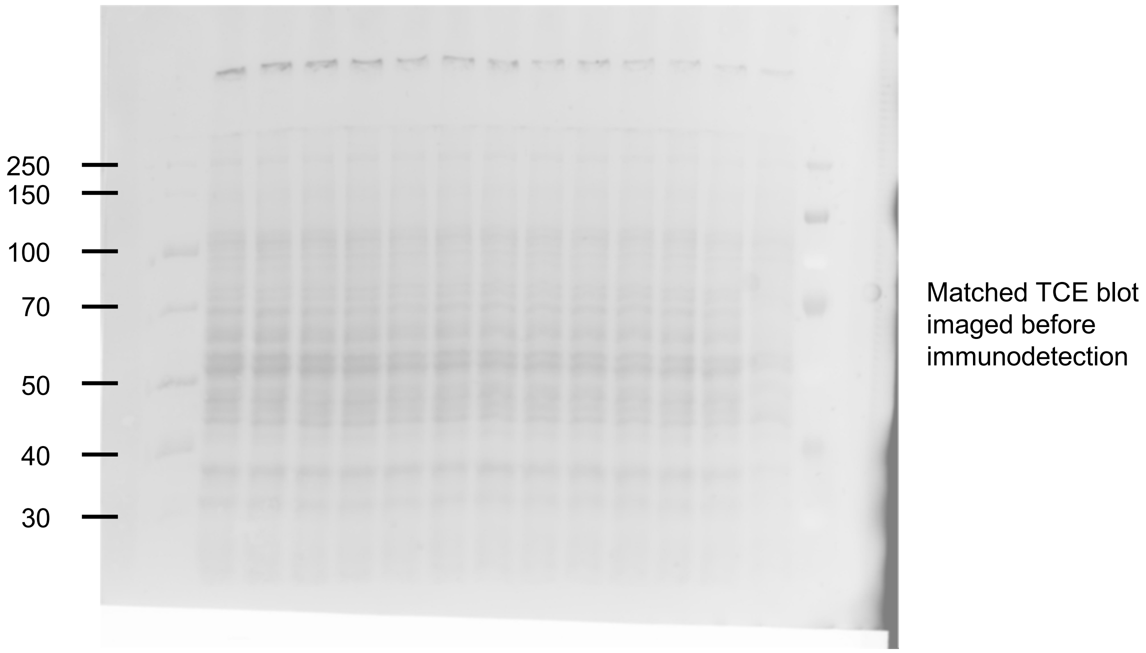

Supplementary Figure 3

p140_TWNK_MGME1_HepaRG_unDiff_Day_0123_n1

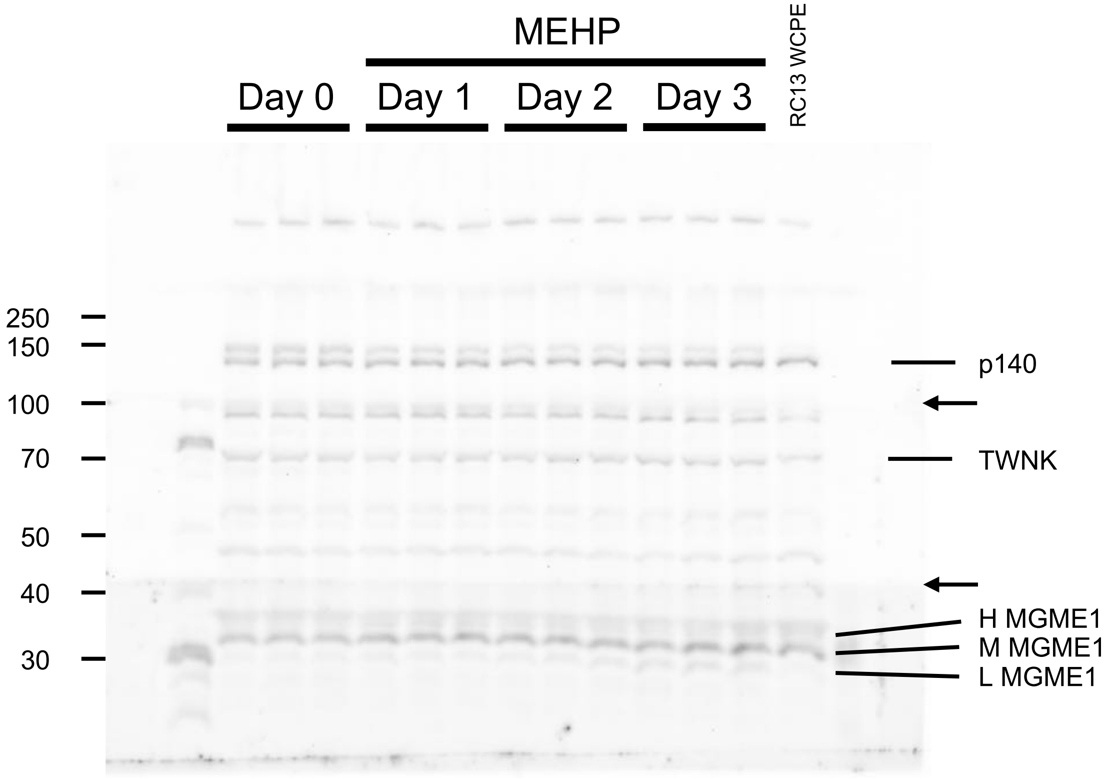

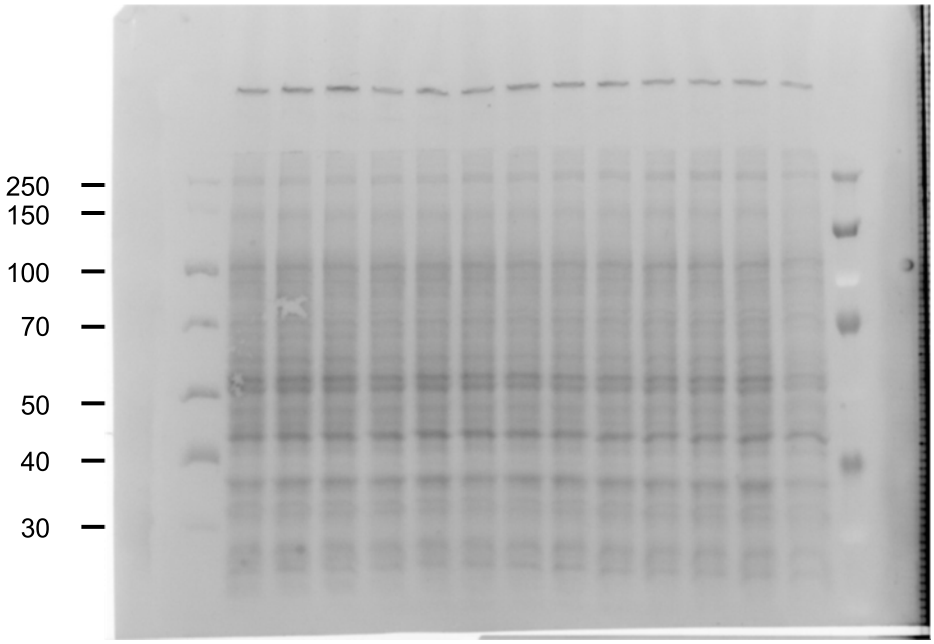

Matched TCE blot imaged before immunodetection

Supplementary Figure 4

p140_TWNK_MGME1_HepaRG_unDiff_Day_0123_n2

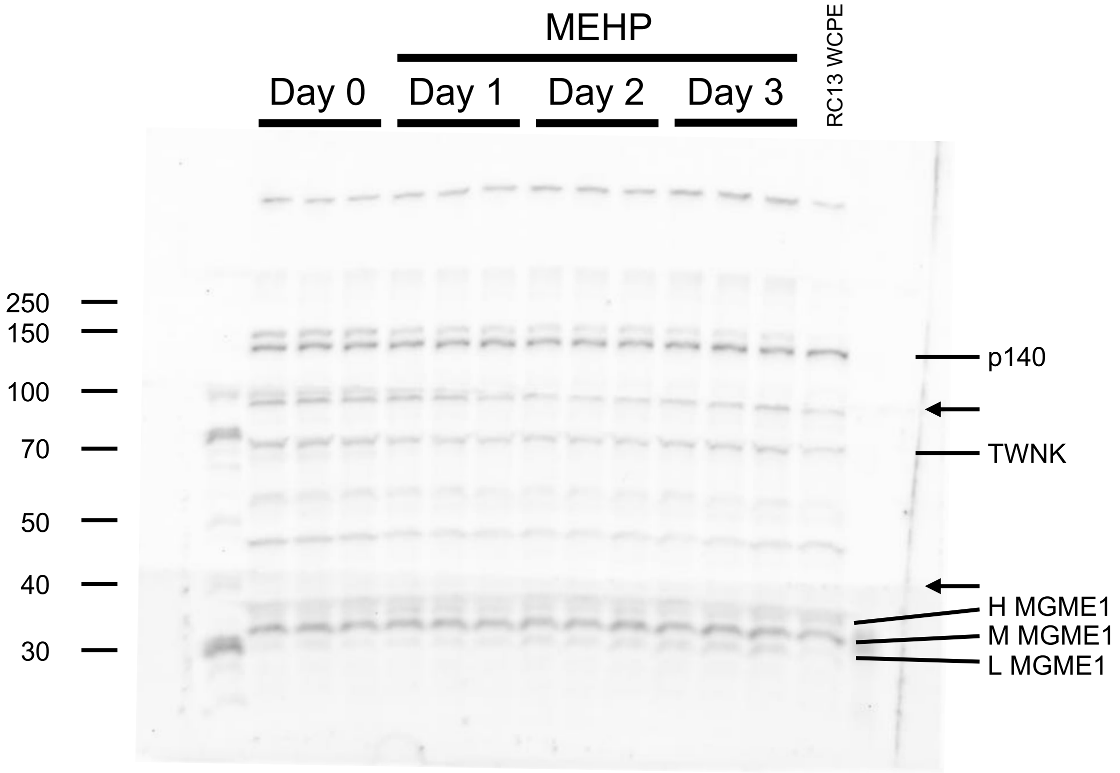

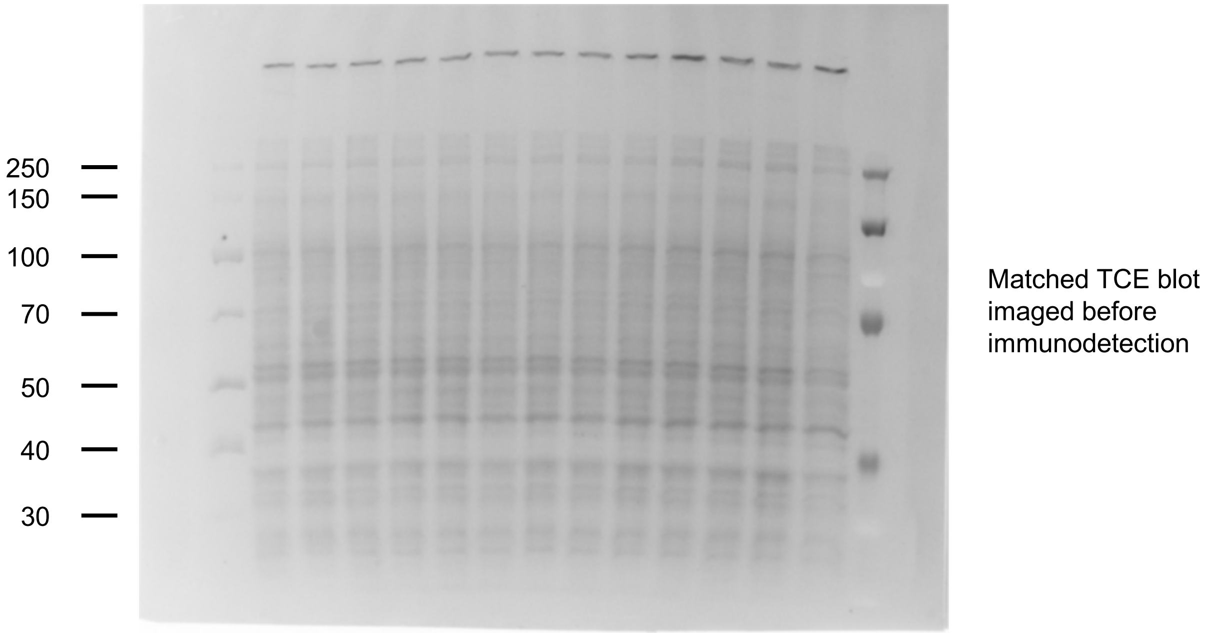

Supplementary Figure 5

p140_TWNK_MGME1_HepaRG_unDiff_Day_0123_n3

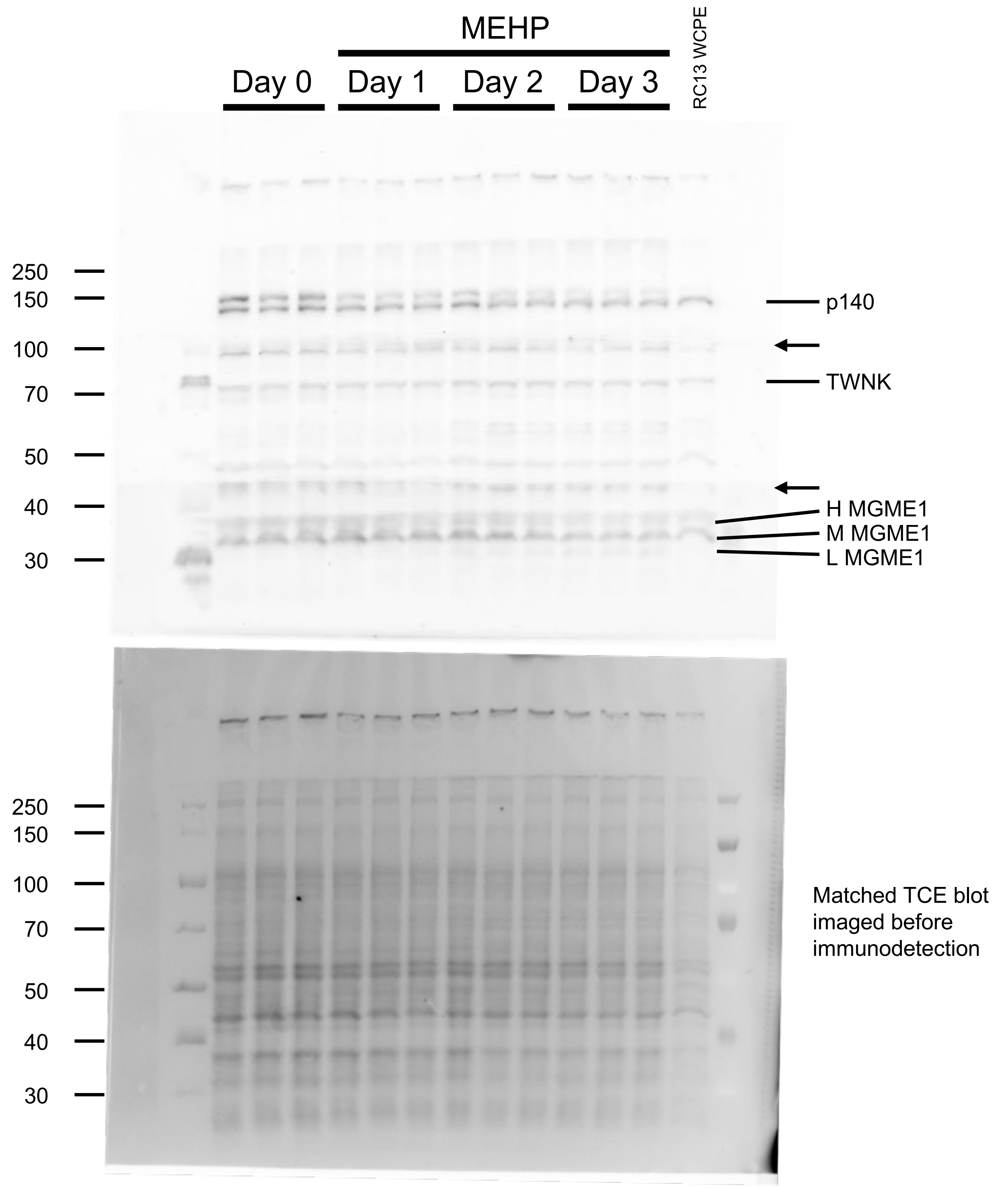

Supplementary Figure 6

PINK1_HepaRG_unDiff_Day_0123_n3

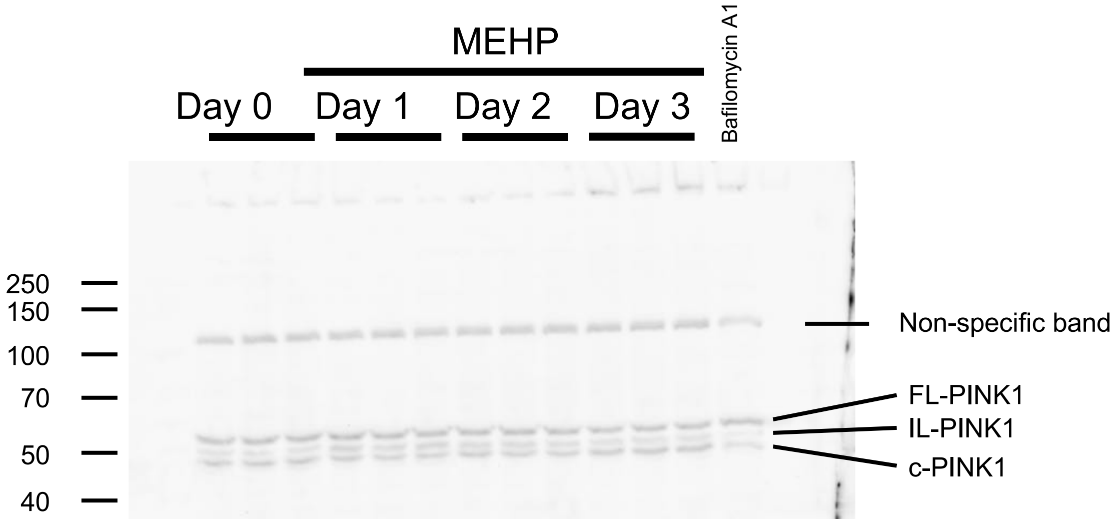

The TCE-stained blot was cut into two strip and only the top strip was used for immunodetection

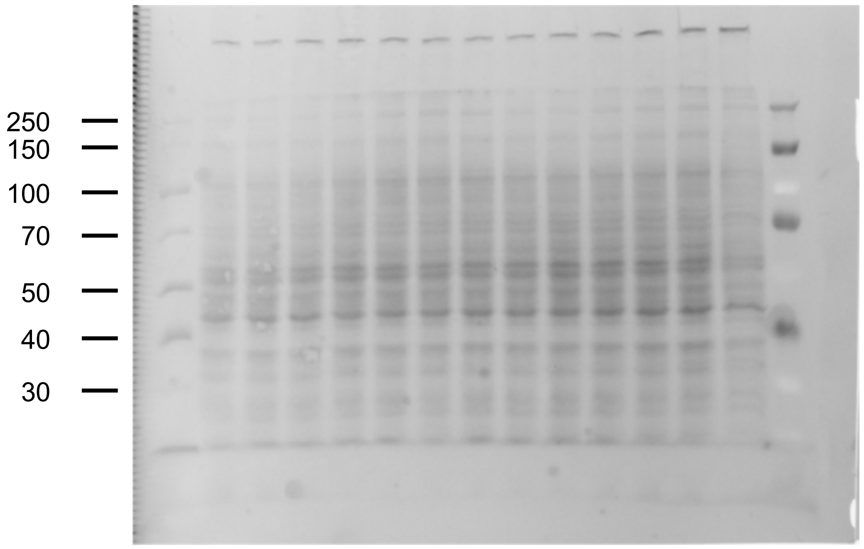

Matched TCE blot imaged before immunodetection

Bafilomycin A1: Cells were treated with 100μM Bafilomycin A1 for 24 hours and whole cell protein extract was run on the corresponding lane.

Supplementary Figure 7

PINK1_HepaRG_unDiff_Day_0123_n1

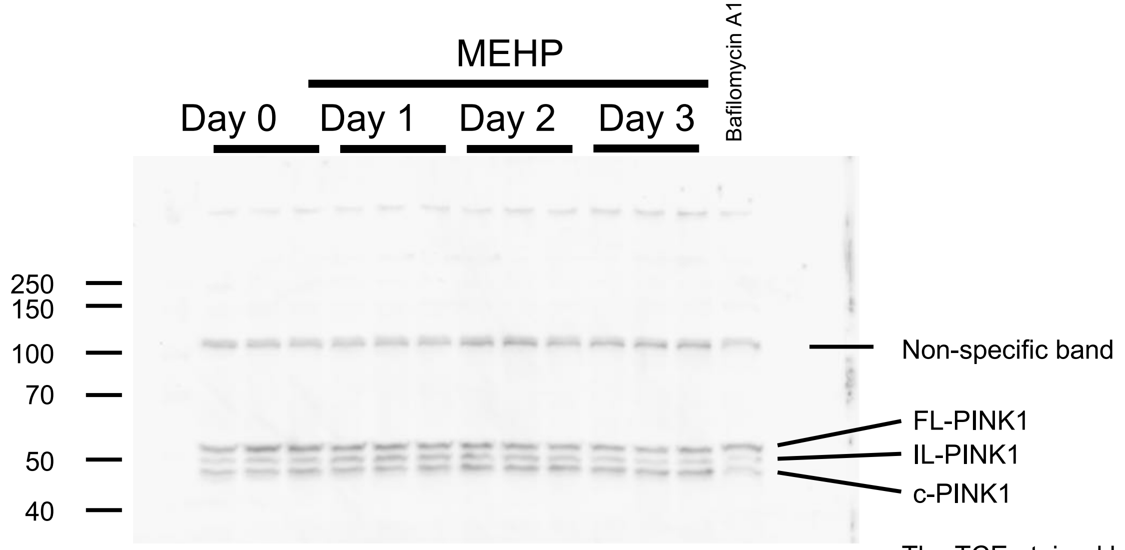

The TCE-stained blot was cut into two strip and only the top strip was used for immunodetection

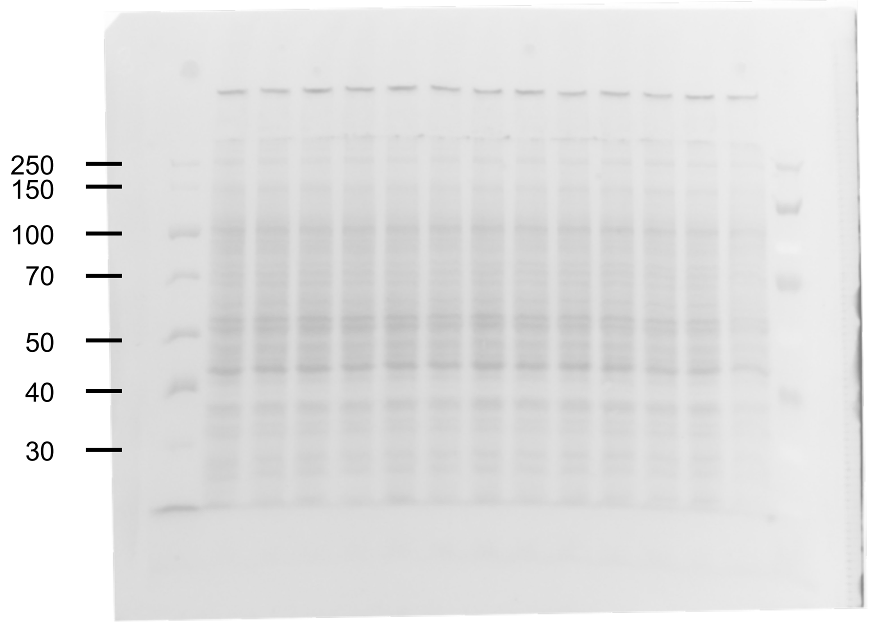

Matched TCE blot imaged before immunodetection

Supplementary Figure 8

PINK1_HepaRG_Diff_Day_0123_n3

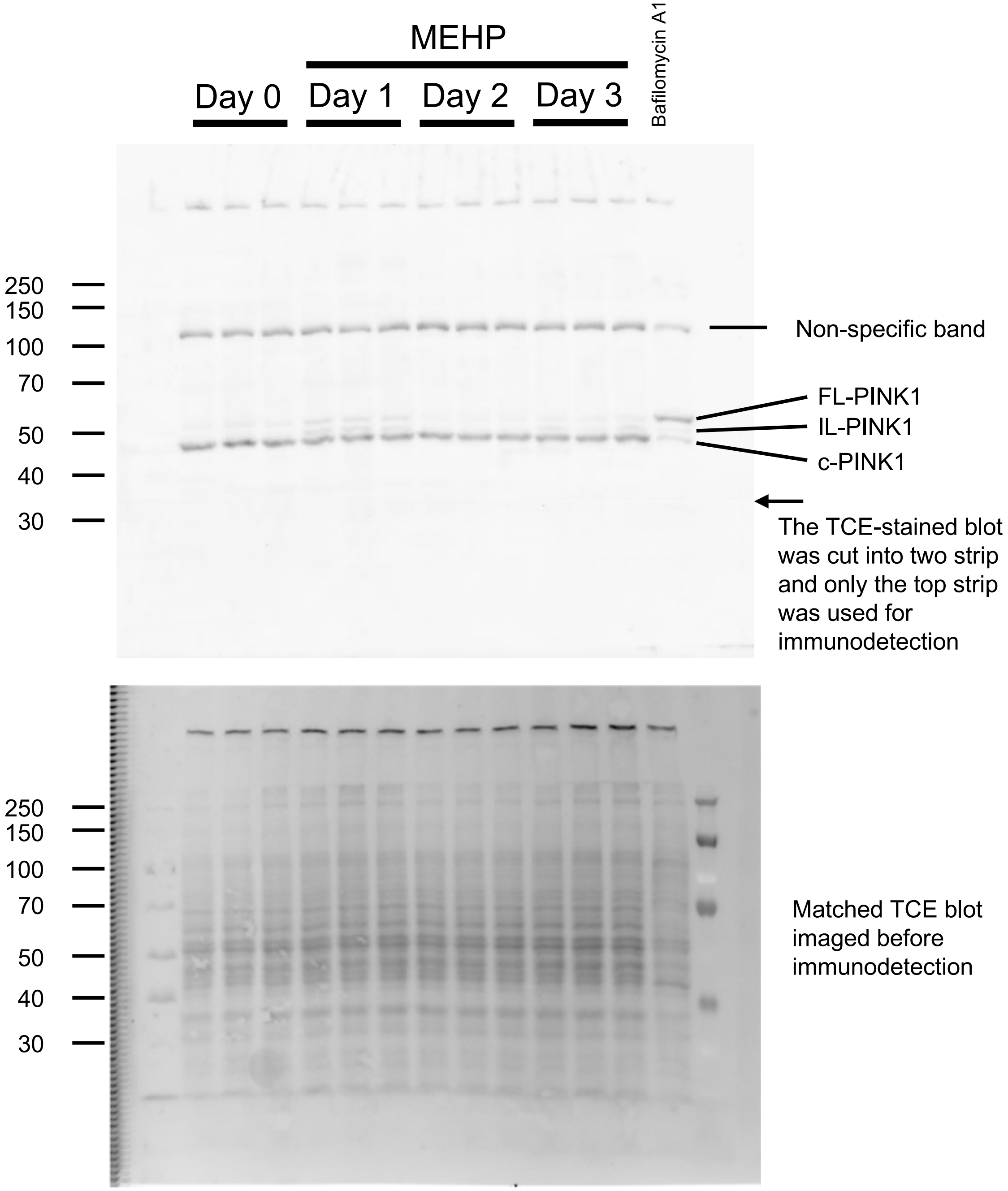

Supplementary Figure 9

PINK1_HepaRG_Diff_Day_0123_n1

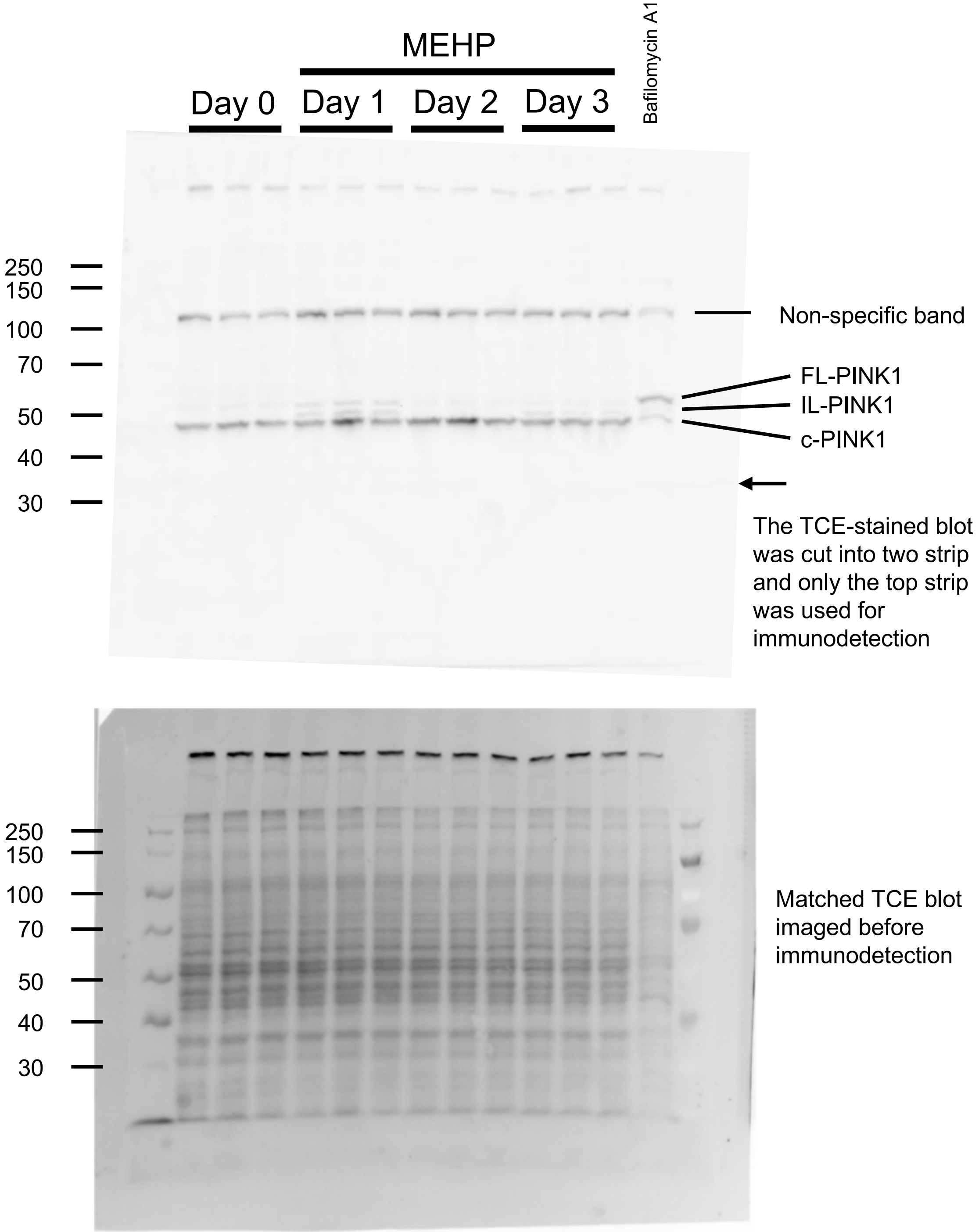

Supplementary Figure 10

PINK1_HepaRG_Diff_Day_0123_n2

BCL-2_HepaRG_Diff_Day_0123_n2

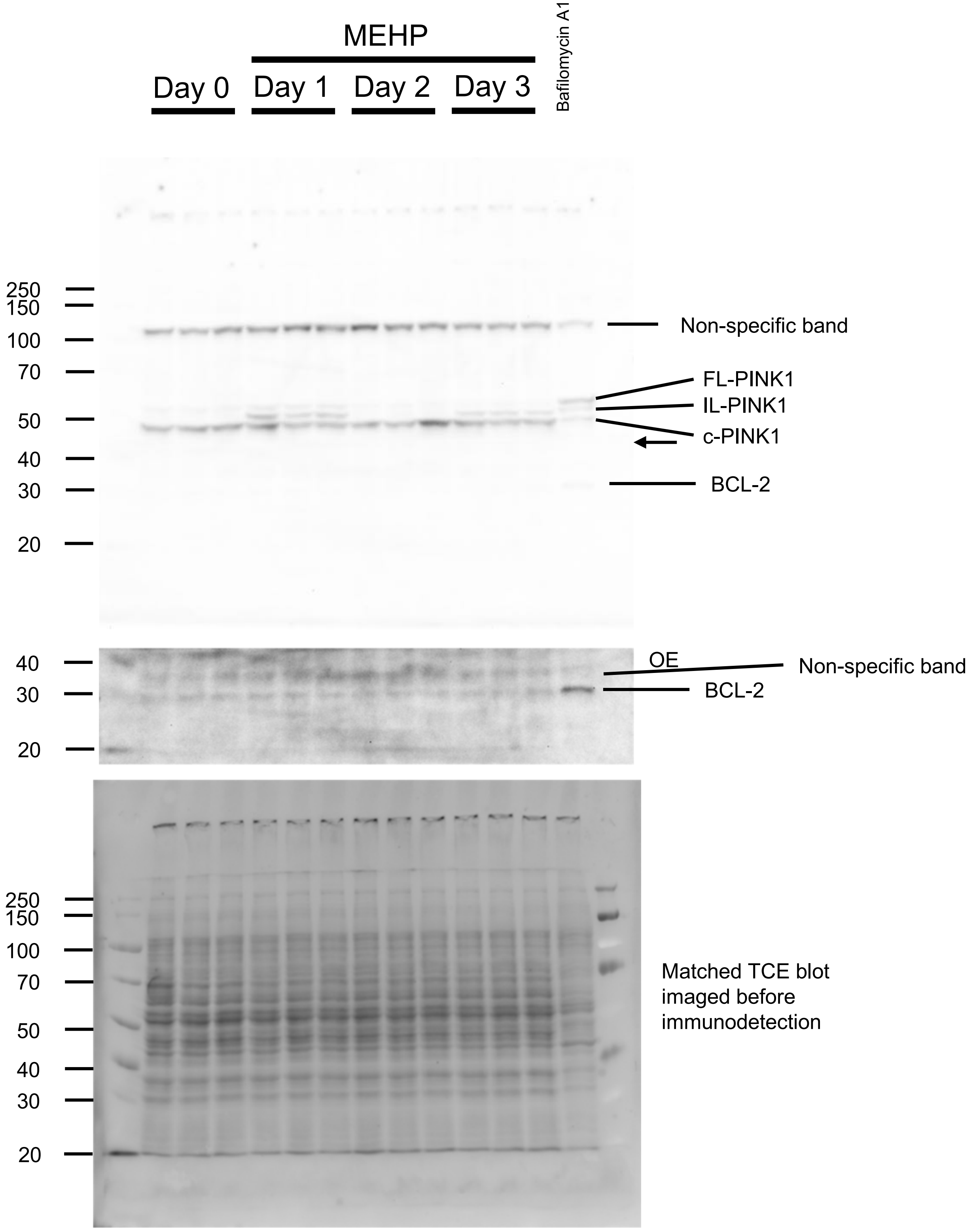

OE: Over Exposed

Supplementary Figure 11

PINK1_HepaRG_unDiff_Day_0123_n2

BCL-2_HepaRG_unDiff_Day_0123_n2

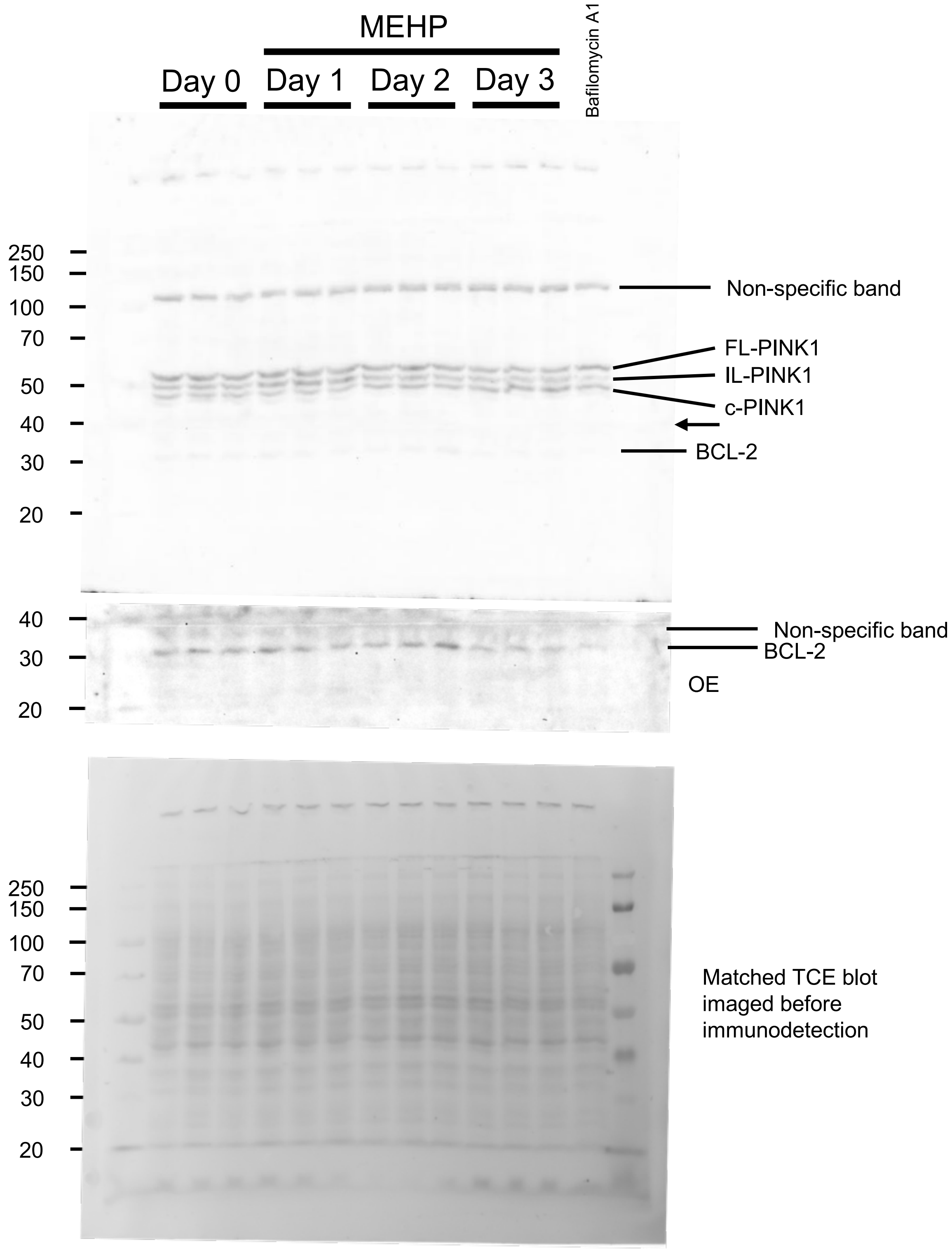

Supplementary Figure 12

p62_HepaRG_unDiff_Day_0123_n1

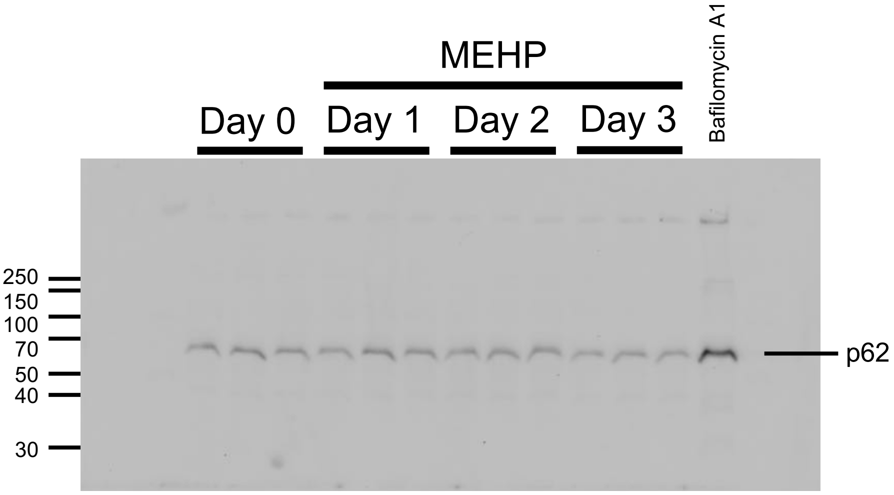

The TCE-stained blot was cut into two strip and only the top strip was used for immunodetection

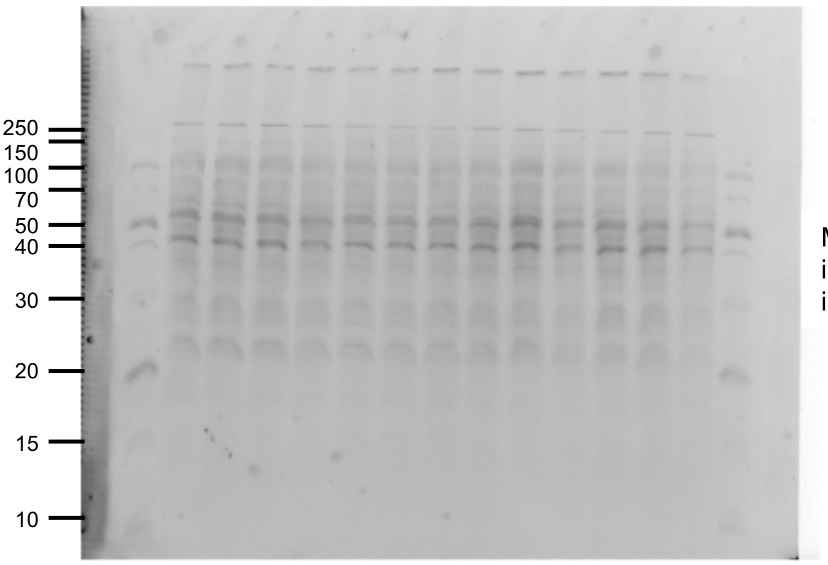

Matched TCE blot imaged before immunodetection

Supplementary Figure 13

p62_HepaRG_Diff_Day_0123_n1

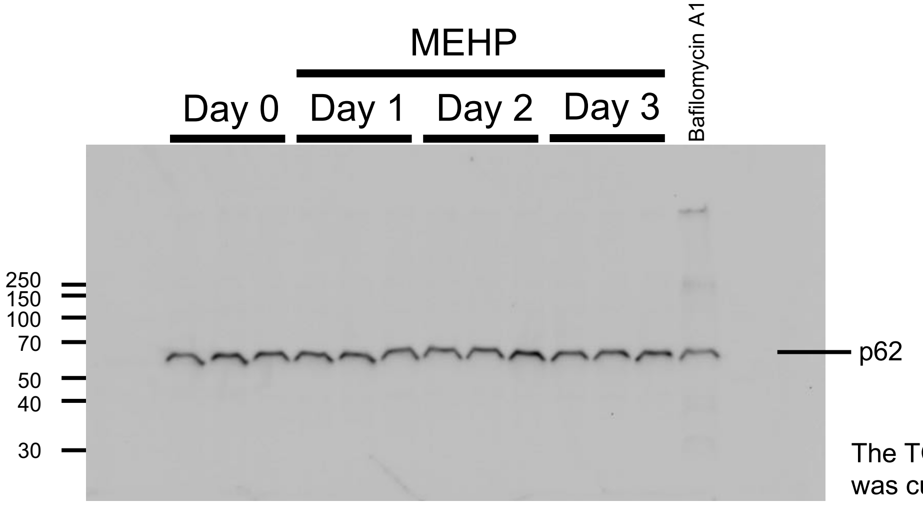

The TCE-stained blot was cut into two strip and only the top strip was used for immunodetection

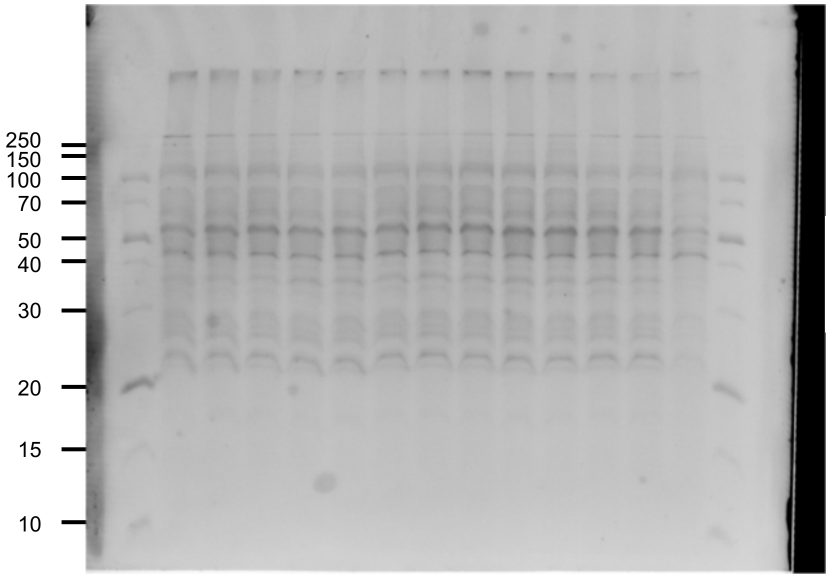

Matched TCE blot imaged before immunodetection

Supplementary Figure 14

p62_HepaRG_unDiff_Day_0123_n2

BAX_HepaRG_unDiff_Day_0123_n2

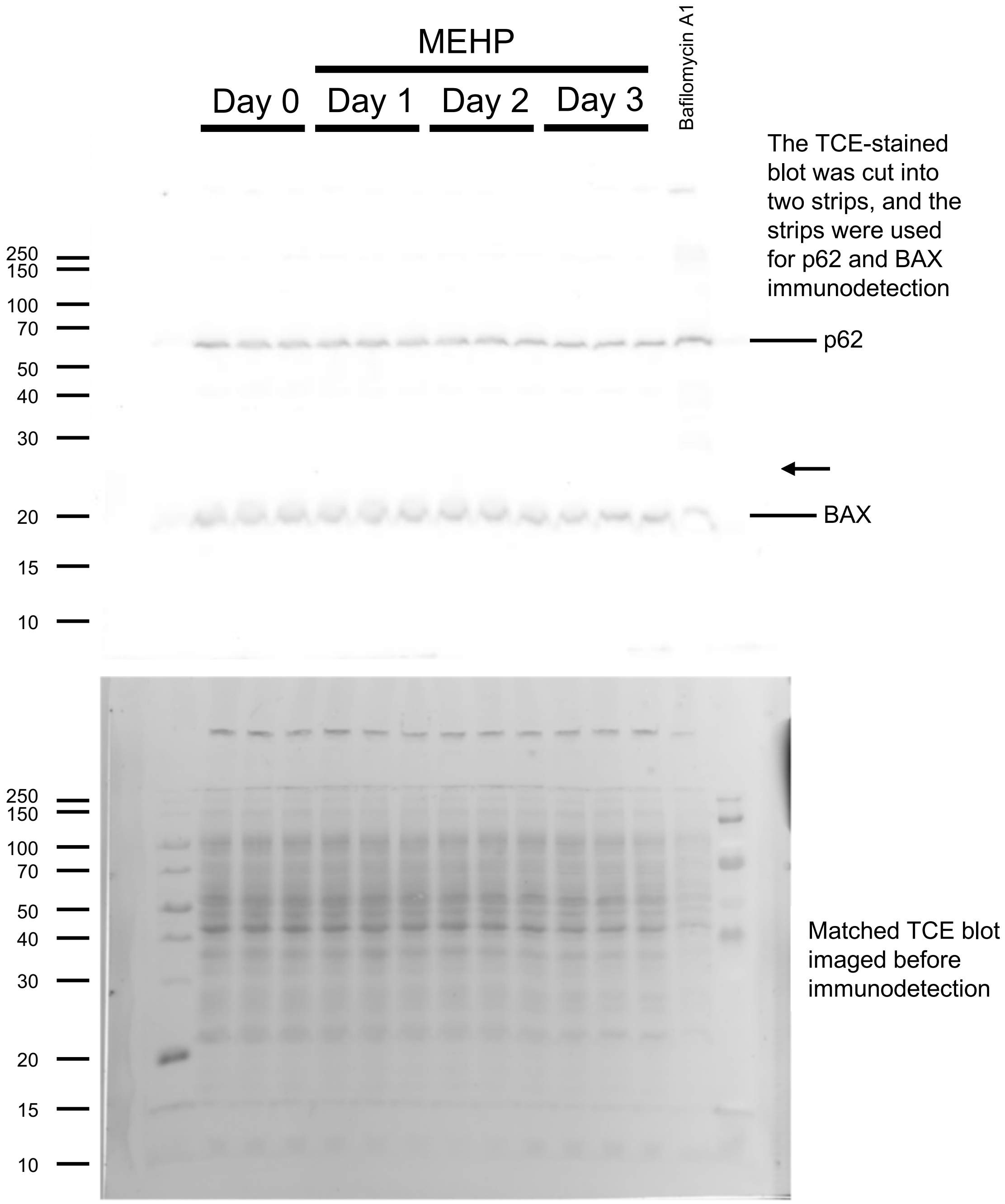

Supplementary Figure 15

p62_HepaRG_Diff_Day_0123_n2
BAX_HepaRG_Diff_Day_0123_n2

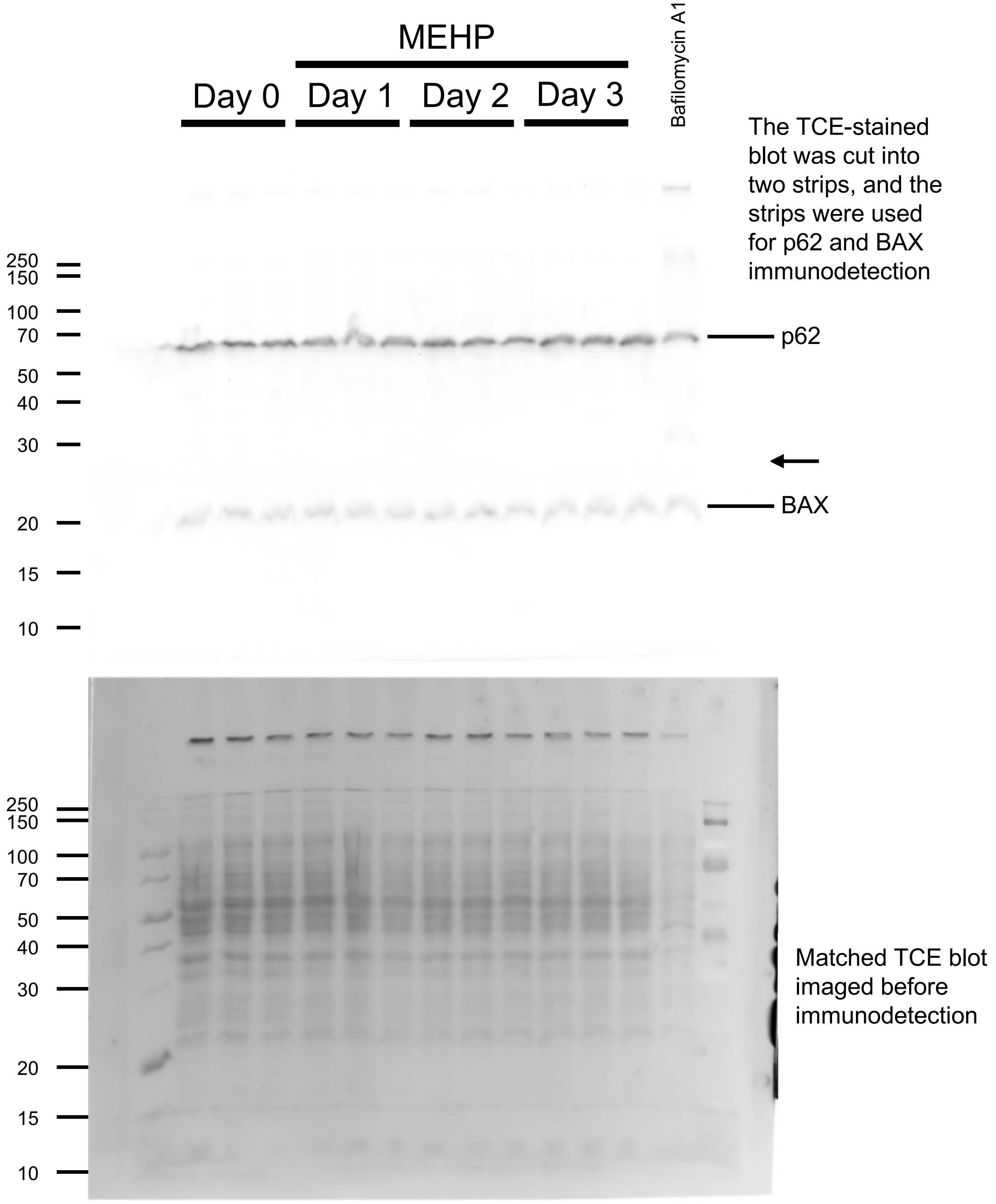

Supplementary Figure 16

p62_HepaRG_unDiff_Day_0123_n3

BAX_HepaRG_unDiff_Day_0123_n3

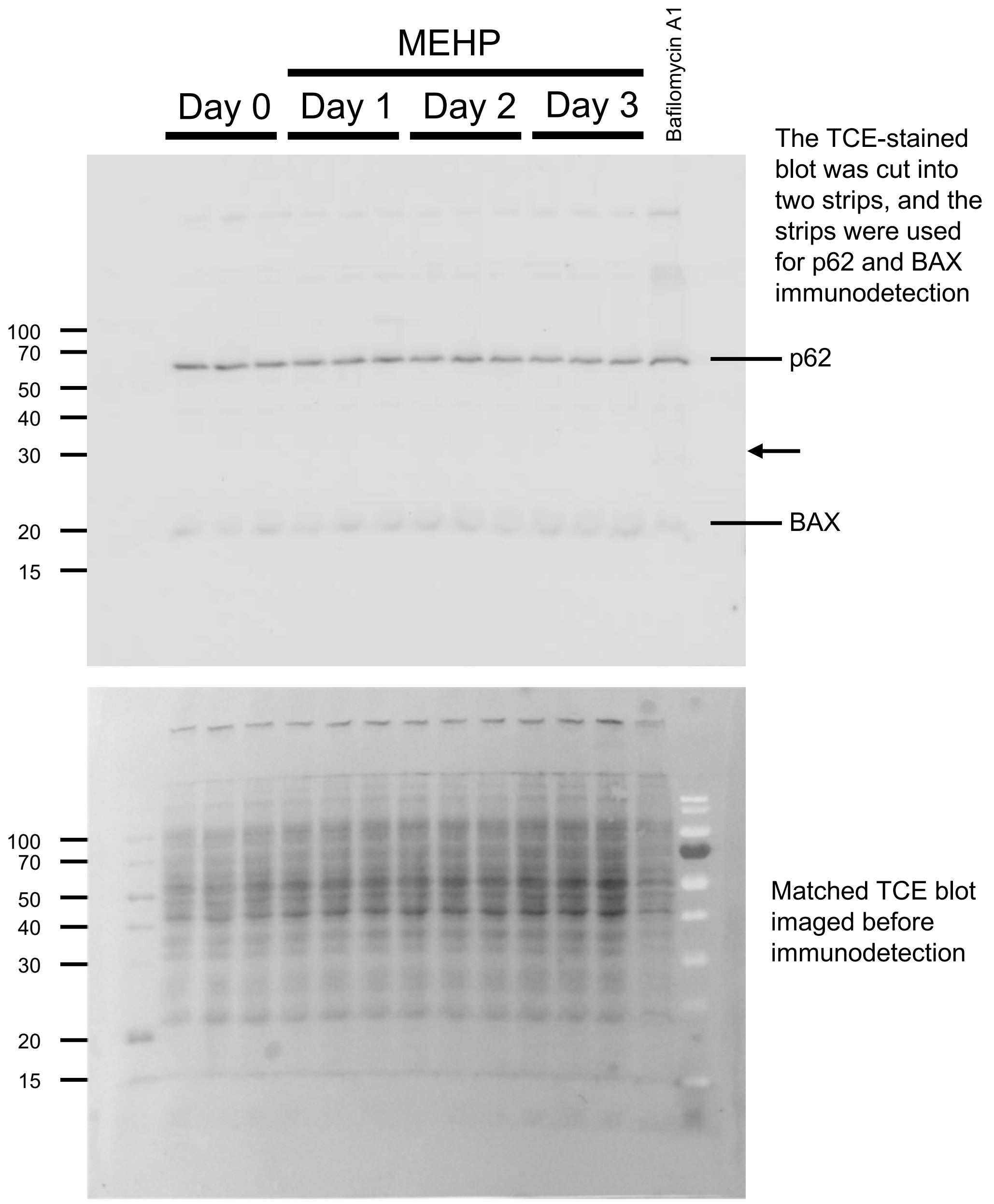

Supplementary Figure 17

p62_HepaRG_Diff_Day_0123_n3

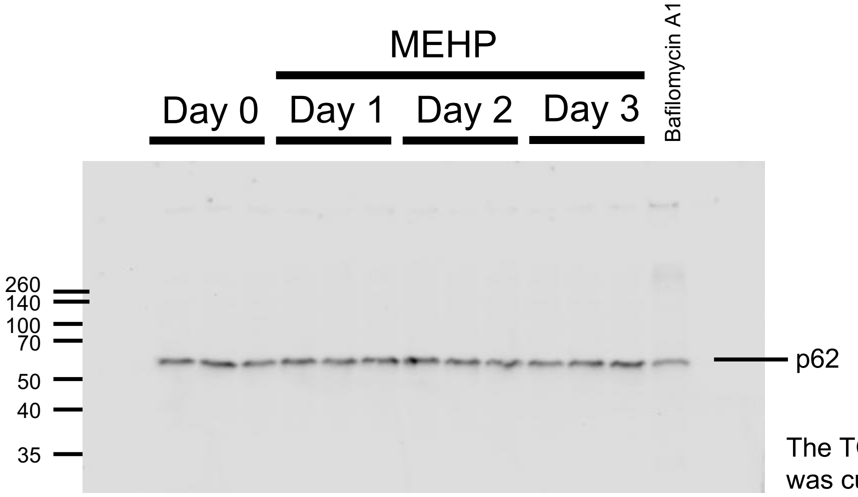

The TCE-stained blot was cut into two strip and only the top strip was used for immunodetection

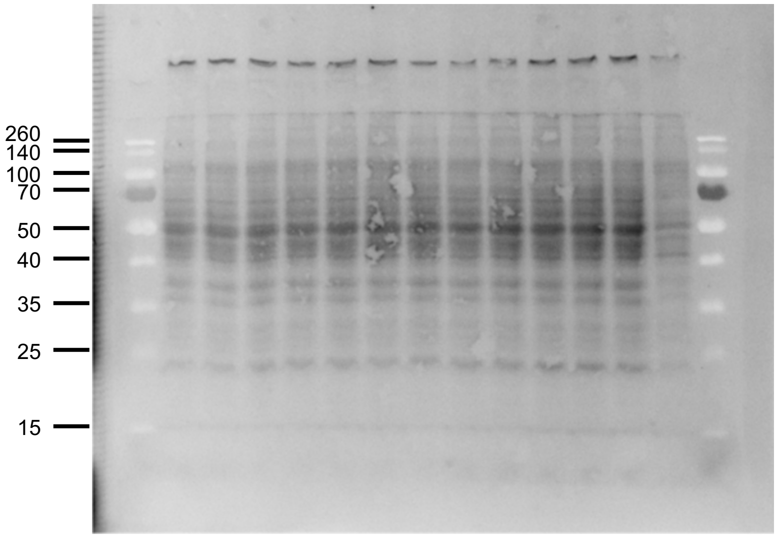

Matched TCE blot imaged before immunodetection

Supplementary Figure 18

Casp-9_HepaRG_unDiff_Day_0123_n1

BAX_HepaRG_unDiff_Day_0123_n1

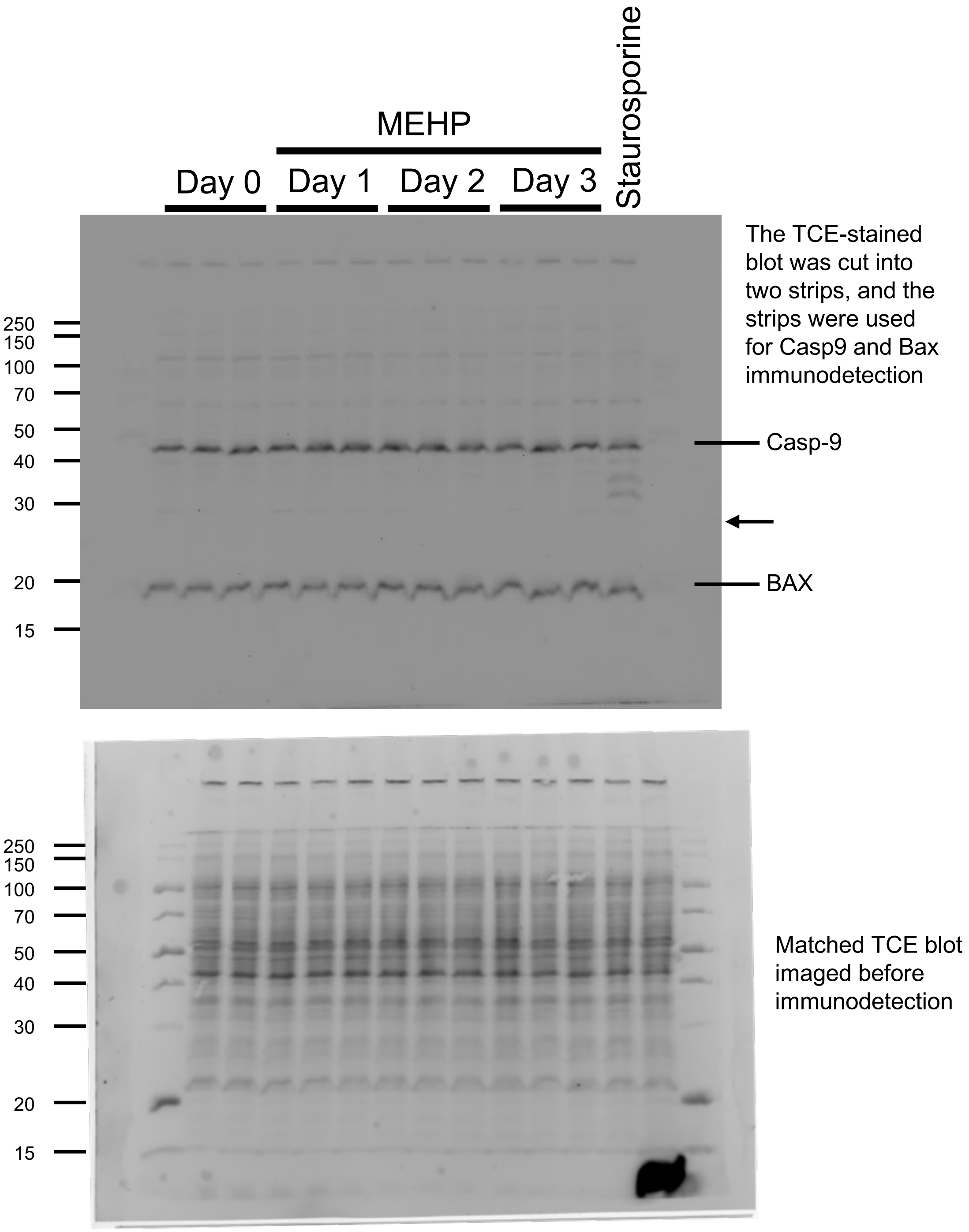

Staurosporine: Cells were treated with 8μM staurosporine for 4 hours, and whole cell protein extract was run on the corresponding lane. Casp9, Caspase 9.

Supplementary Figure 19

Casp-9_HepaRG_Diff_Day_0123_n1

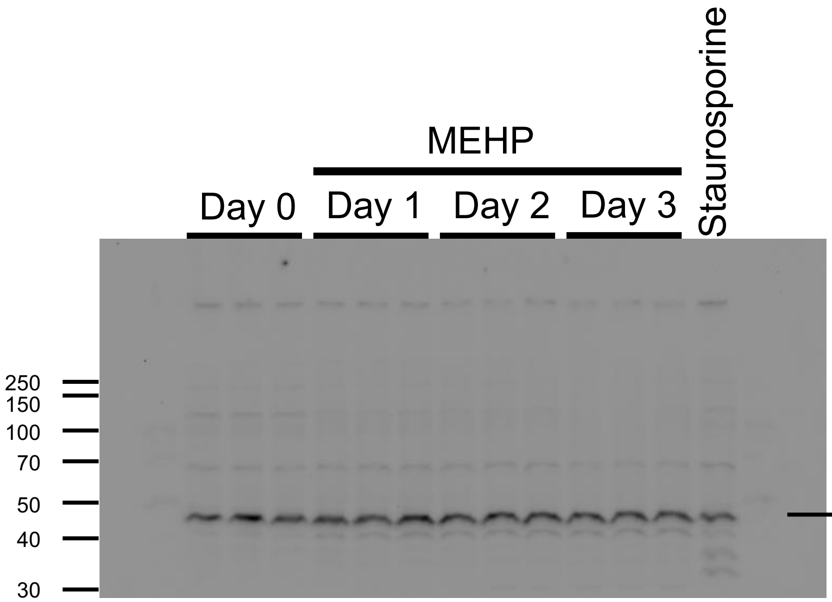

The TCE-stained blot was cut into two strip and only the top strip was used for immunodetection

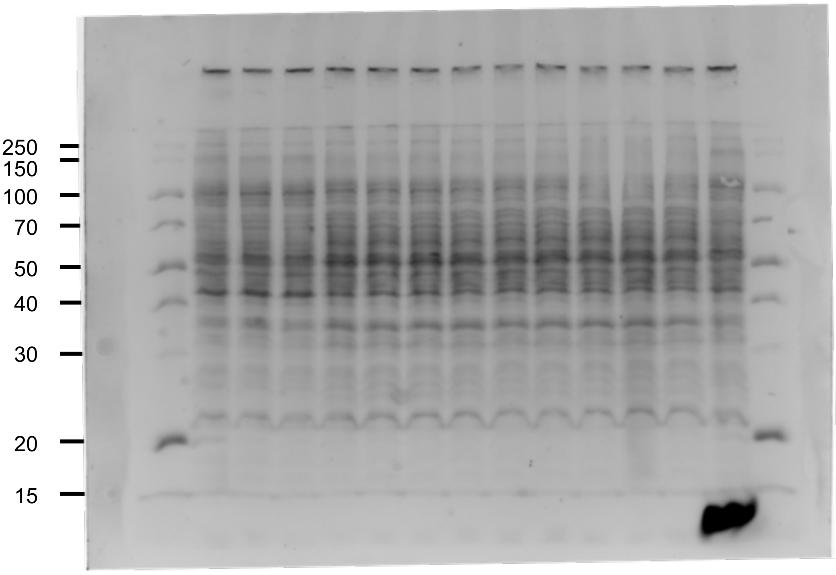

Matched TCE blot imaged before immunodetection

Supplementary Figure 20

CCasp-9_HepaRG_unDiff_Day_0123_n1

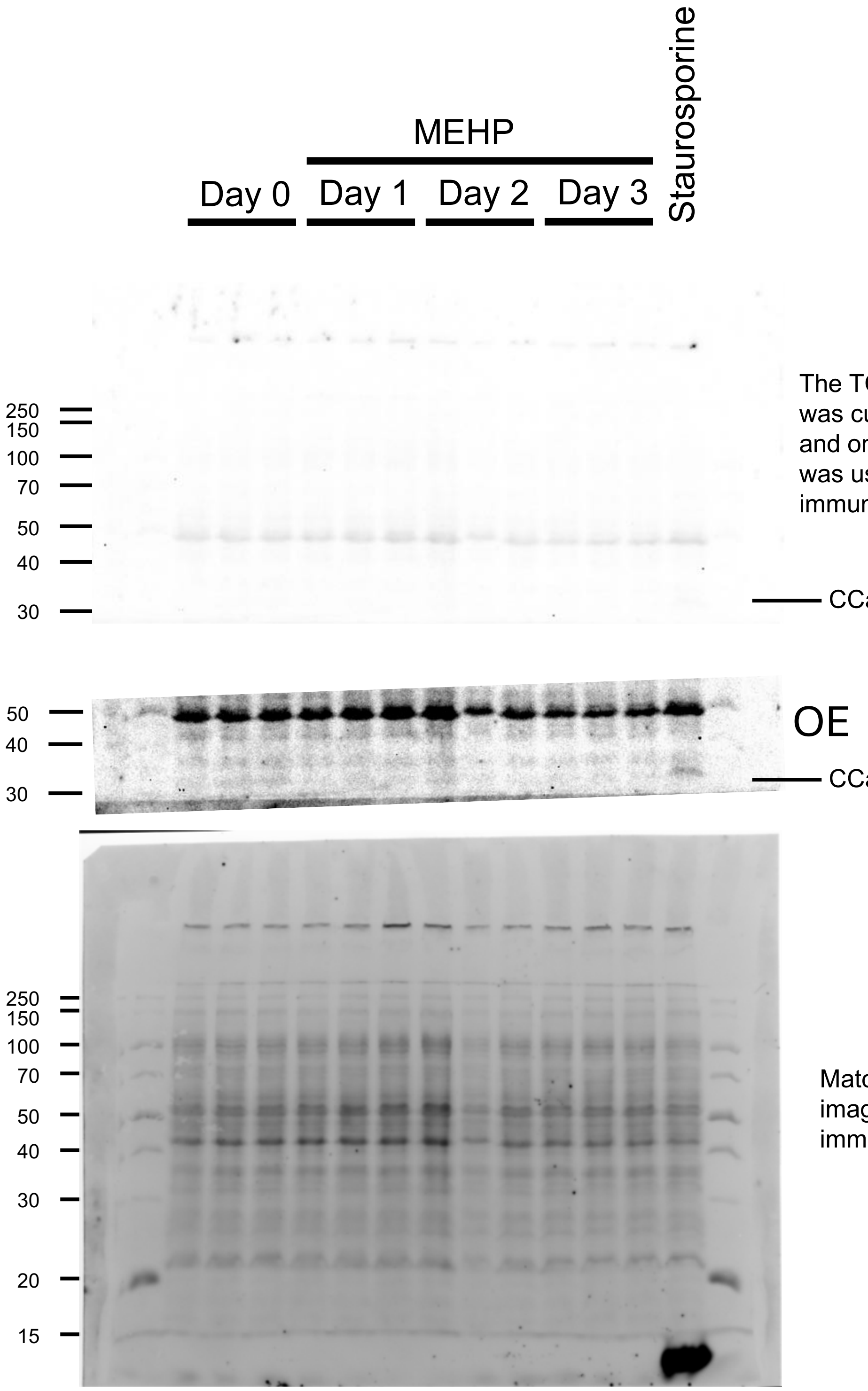

The TCE-stained blot was cut into two strip and only the top strip was used for immunodetection

Matched TCE blot imaged before immunodetection

CCasp-9: Cleaved Caspase 9

Supplementary Figure 21

CCasp-9_HepaRG_Diff_Day_0123_n1

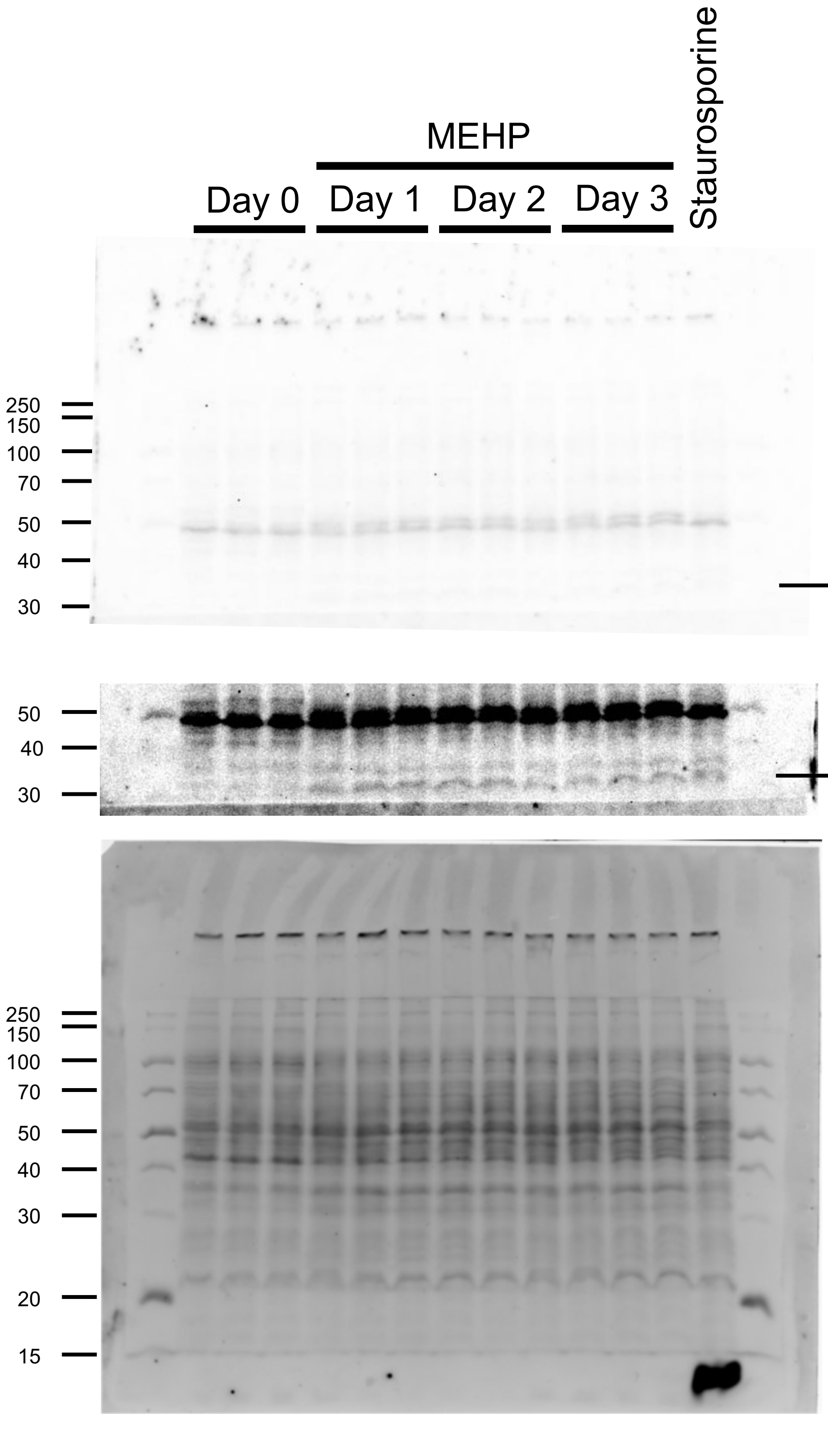

The TCE-stained blot was cut into two strip and only the top strip was used for immunodetection

Matched TCE blot imaged before immunodetection

Supplementary Figure 22

Casp-9_HepaRG_unDiff_Day_0123_n2

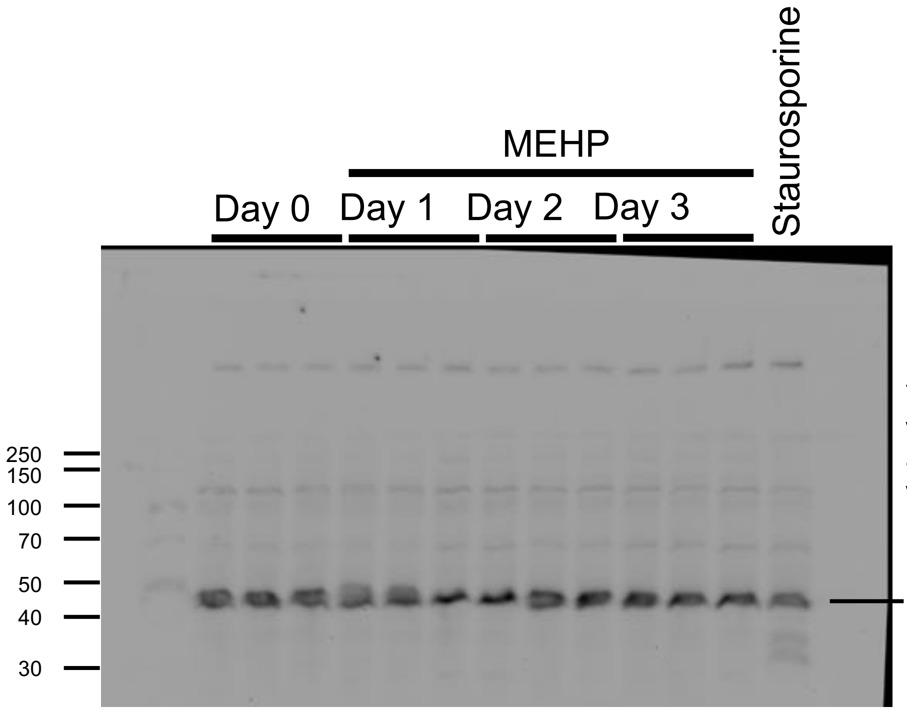

The TCE-stained blot was cut into two strip and only the top strip was used for immunodetection

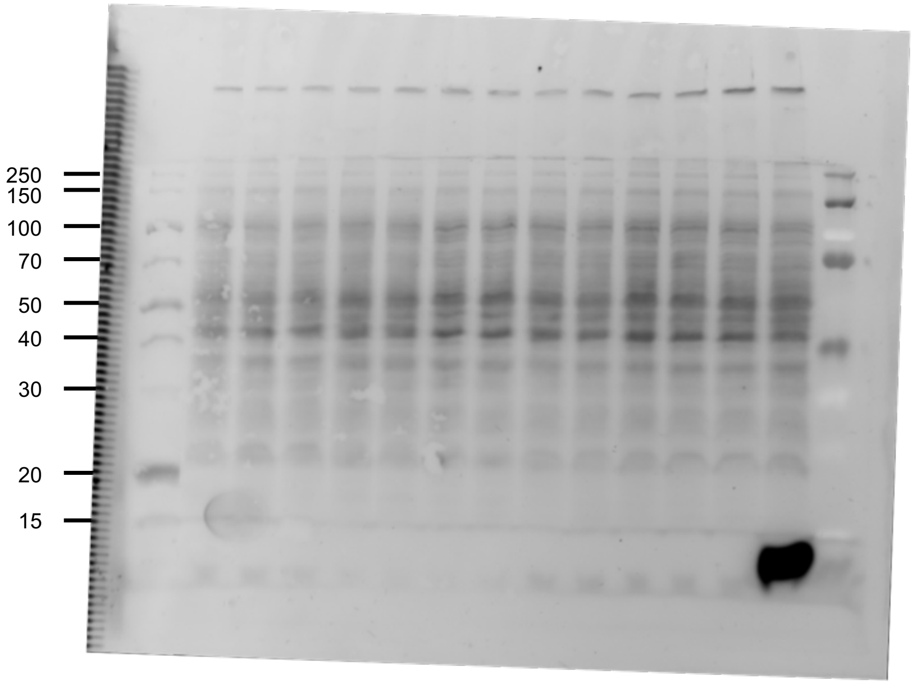

Matched TCE blot imaged before immunodetection

Supplementary Figure 23

Casp-9_HepaRG_Diff_Day_0123_n2

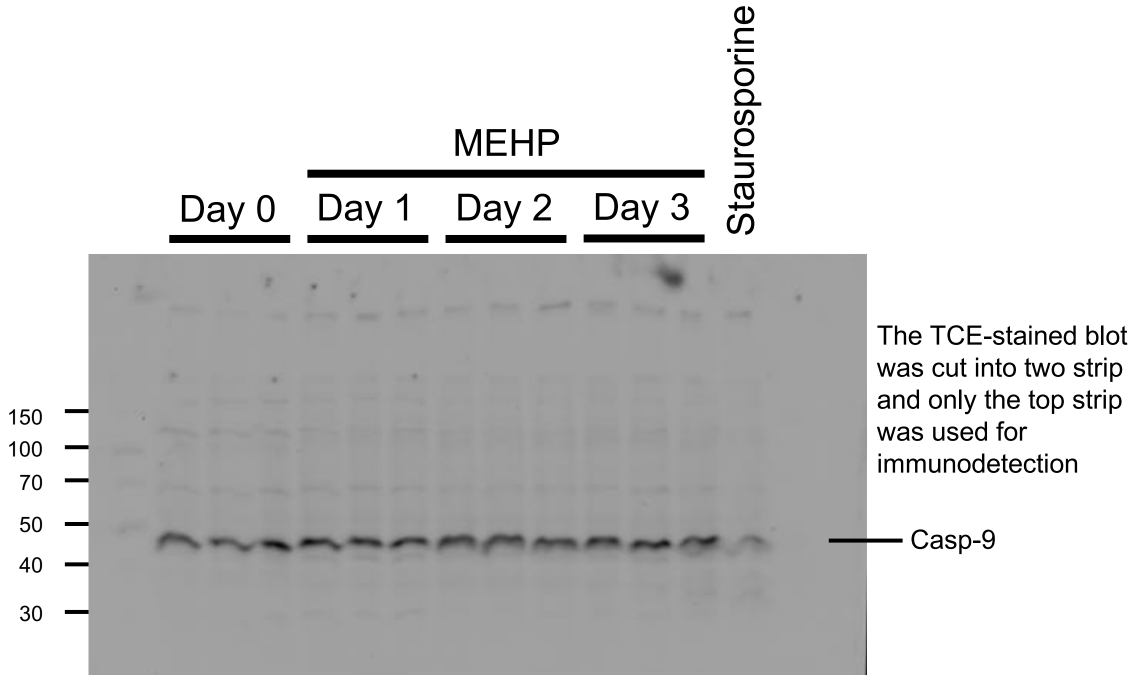

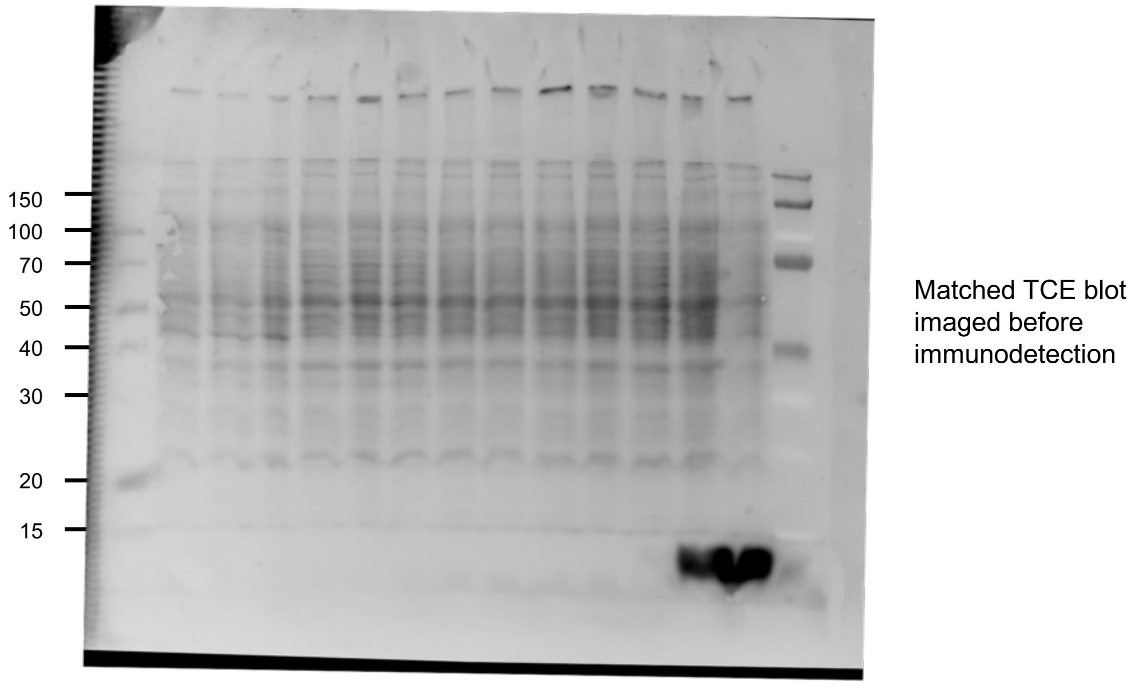

Supplementary Figure 24

CCasp-9_HepaRG_unDiff_Day_0123_n2

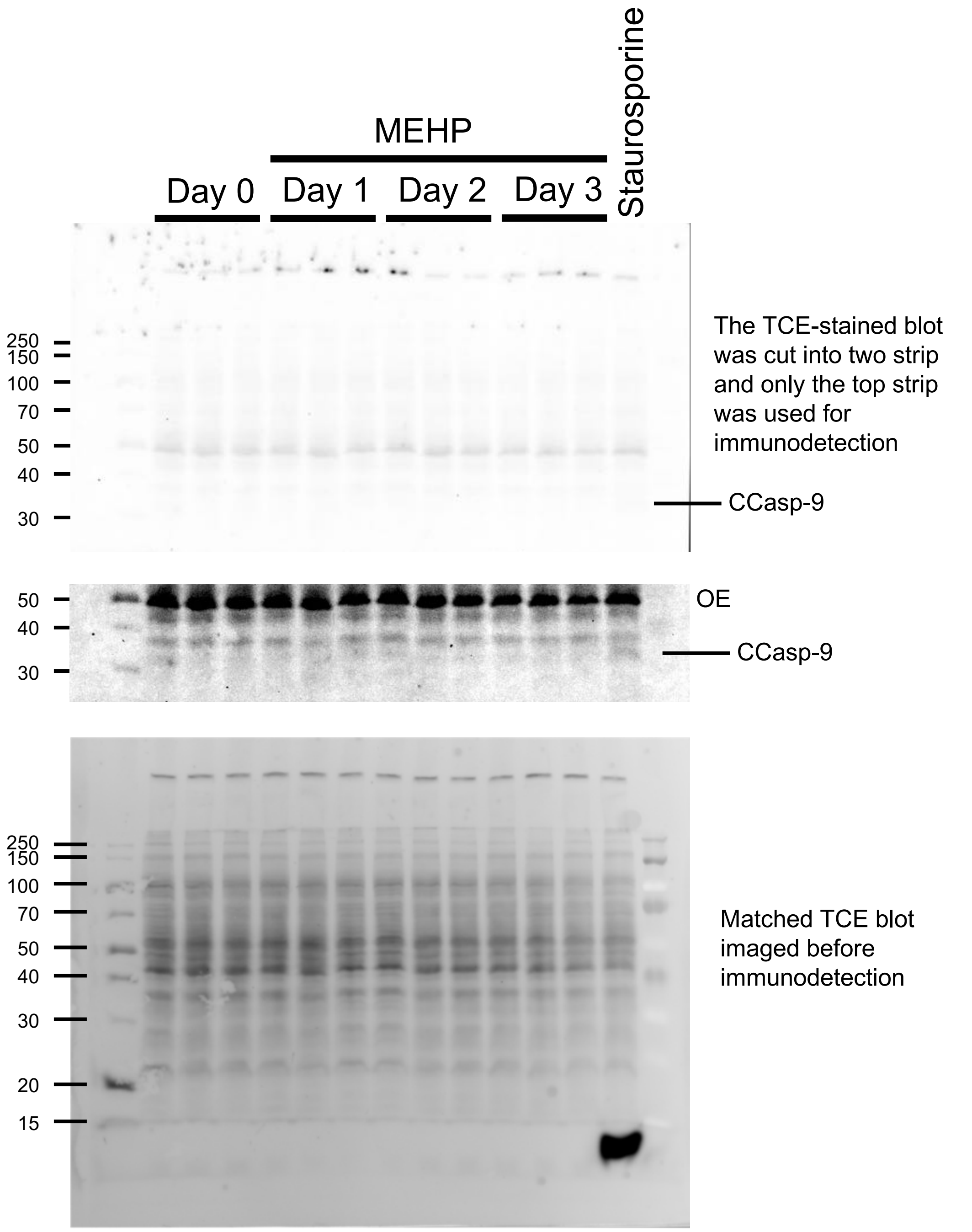

Supplementary Figure 25

CCasp-9_HepaRG_Diff_Day_0123_n2

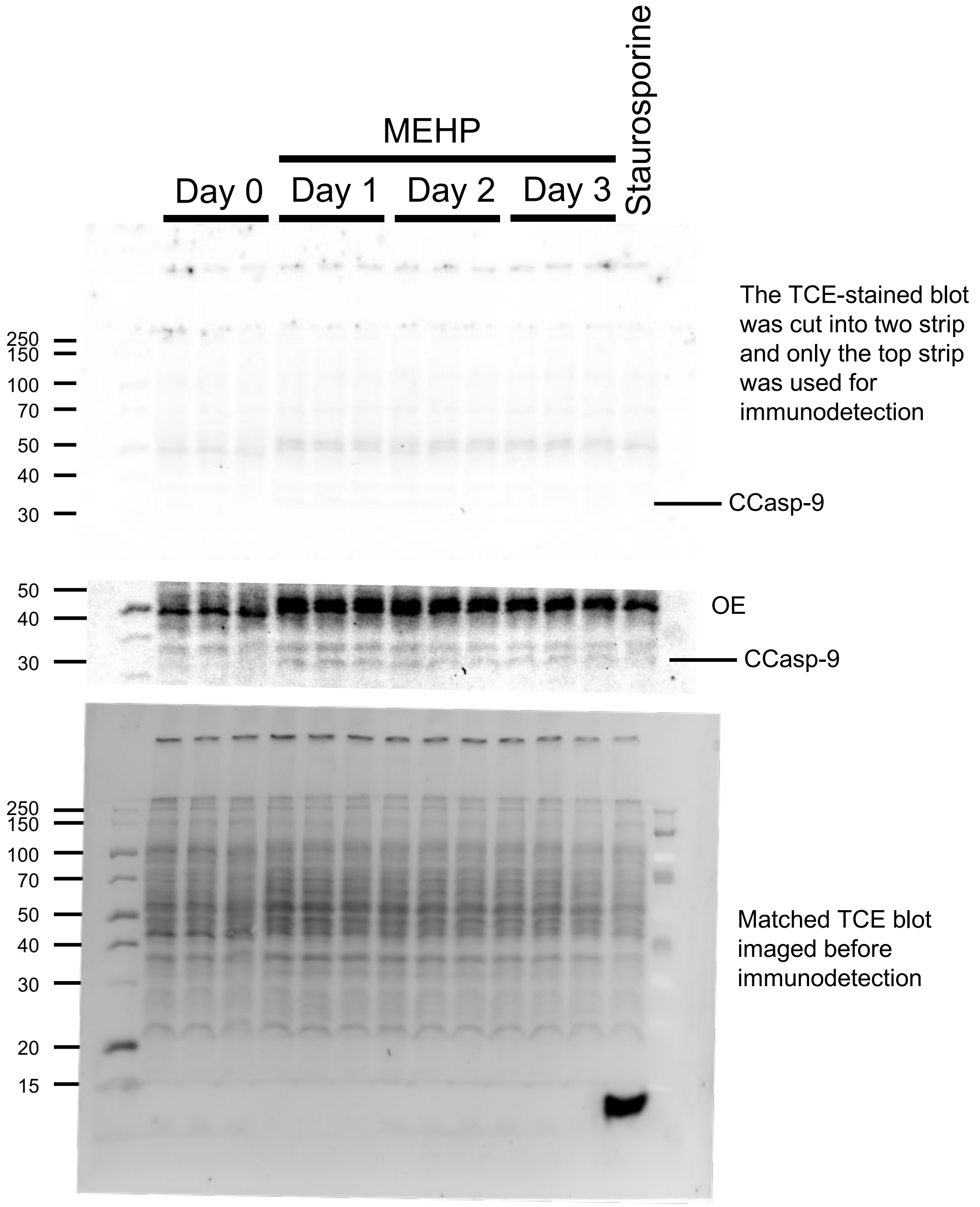

Supplementary Figure 26

Casp-9_HepaRG_unDiff_Day_0123_n3

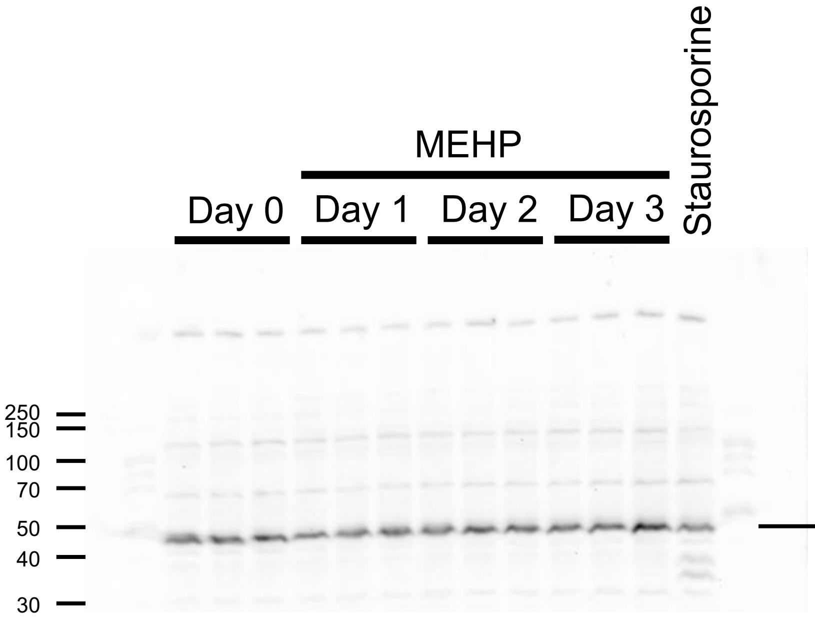

The TCE-stained blot was cut into two strip and only the top strip was used for immunodetection

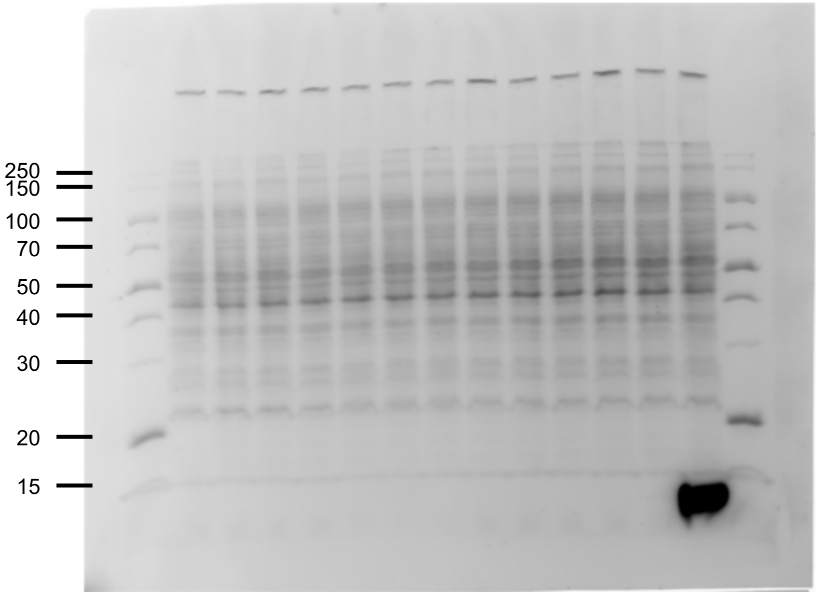

Matched TCE blot imaged before immunodetection

Supplementary Figure 27

Casp-9_HepaRG_Diff_Day_0123_n3

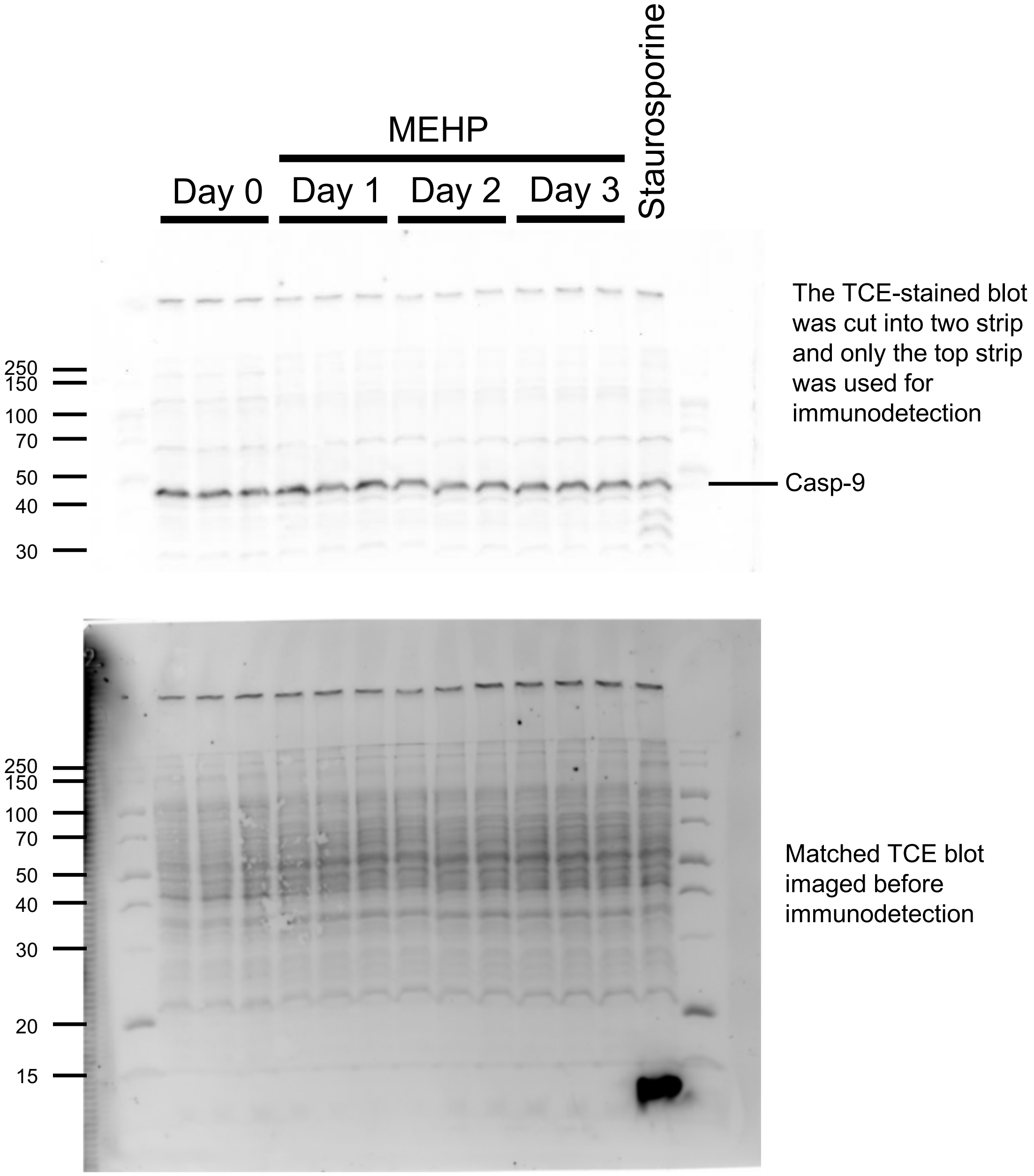

Supplementary Figure 28

CCasp-9_HepaRG_unDiff_Day_0123_n3

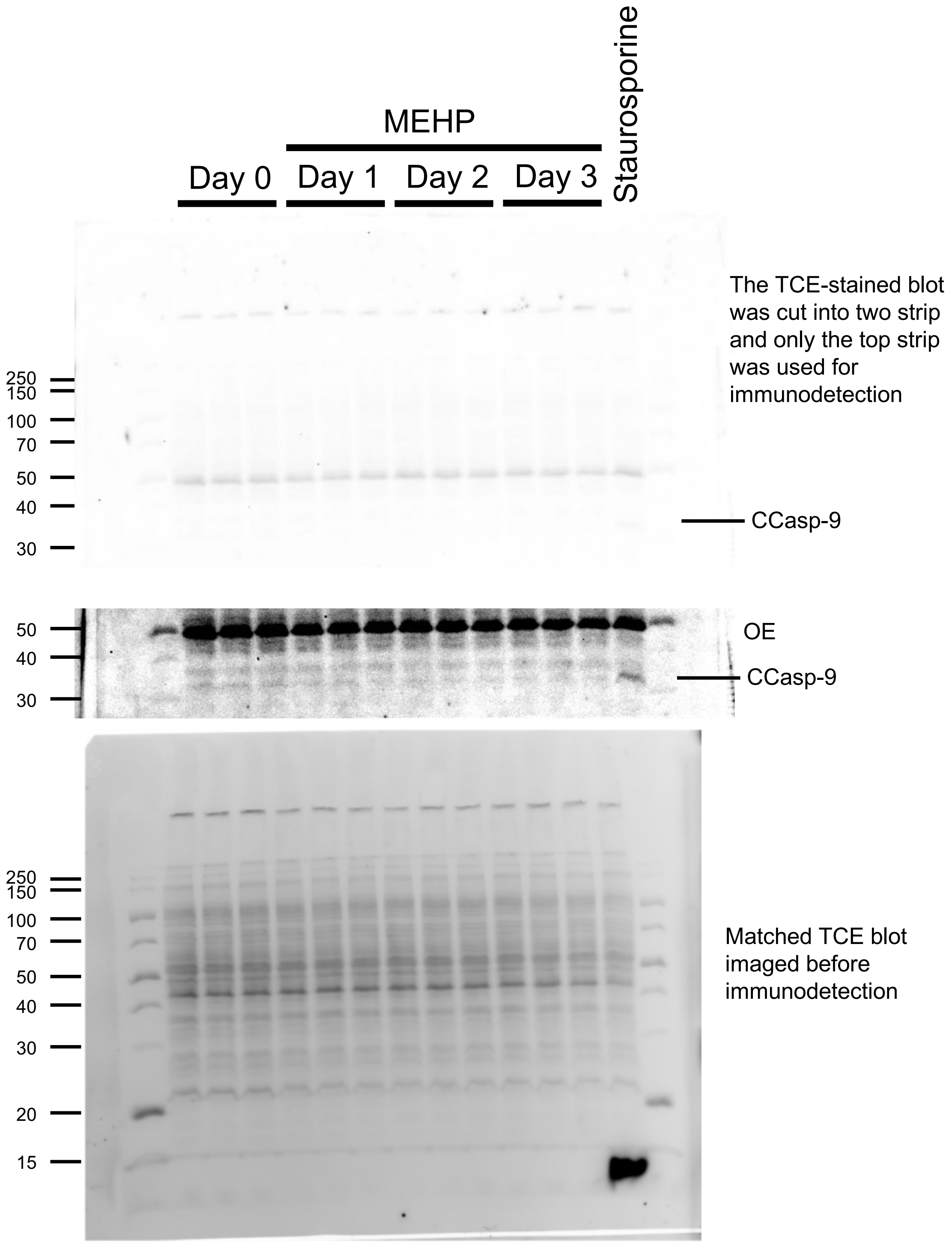

Supplementary Figure 29

CCasp-9_HepaRG_Diff_Day_0123_n3

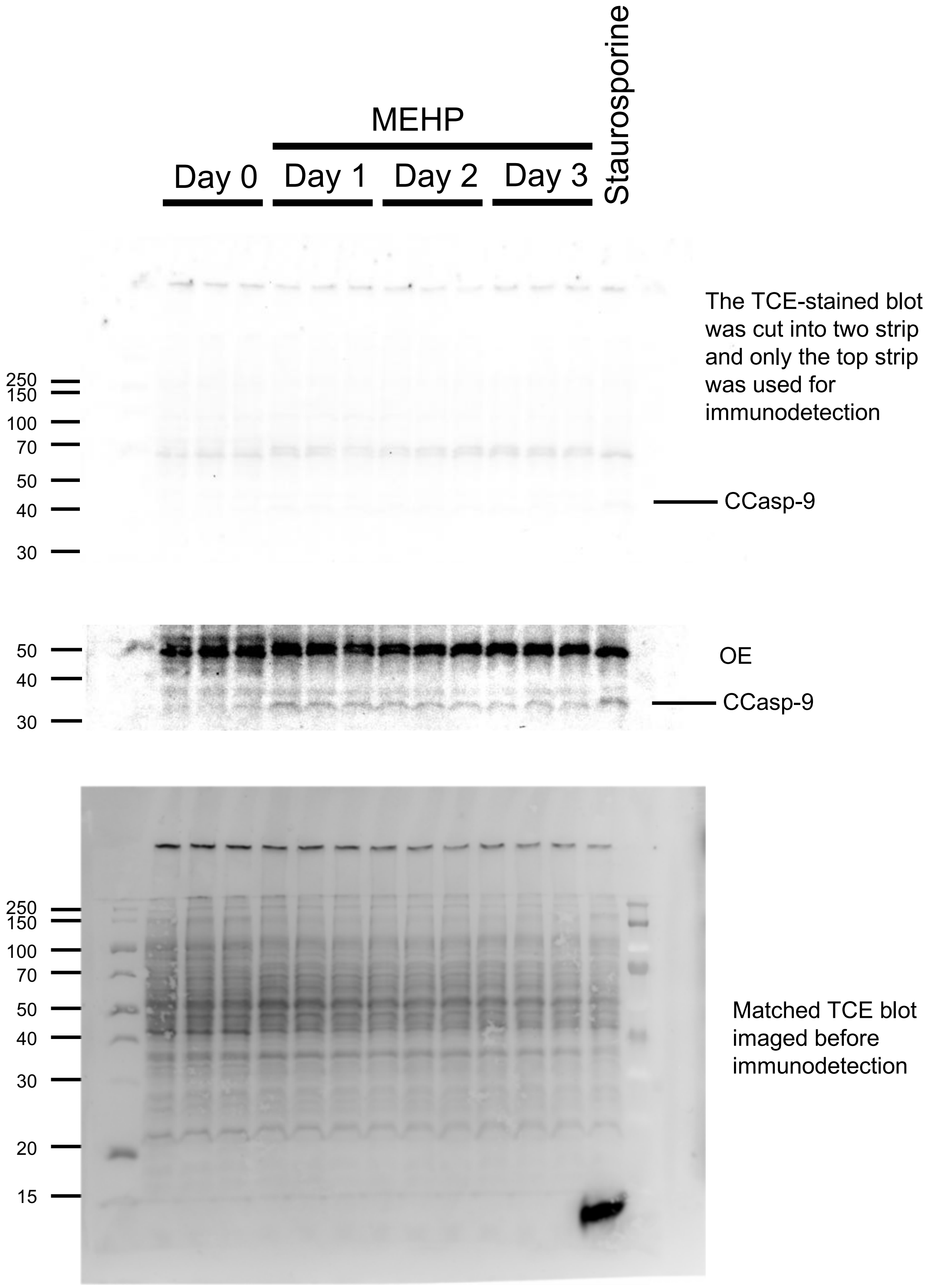

Supplementary Figure 30

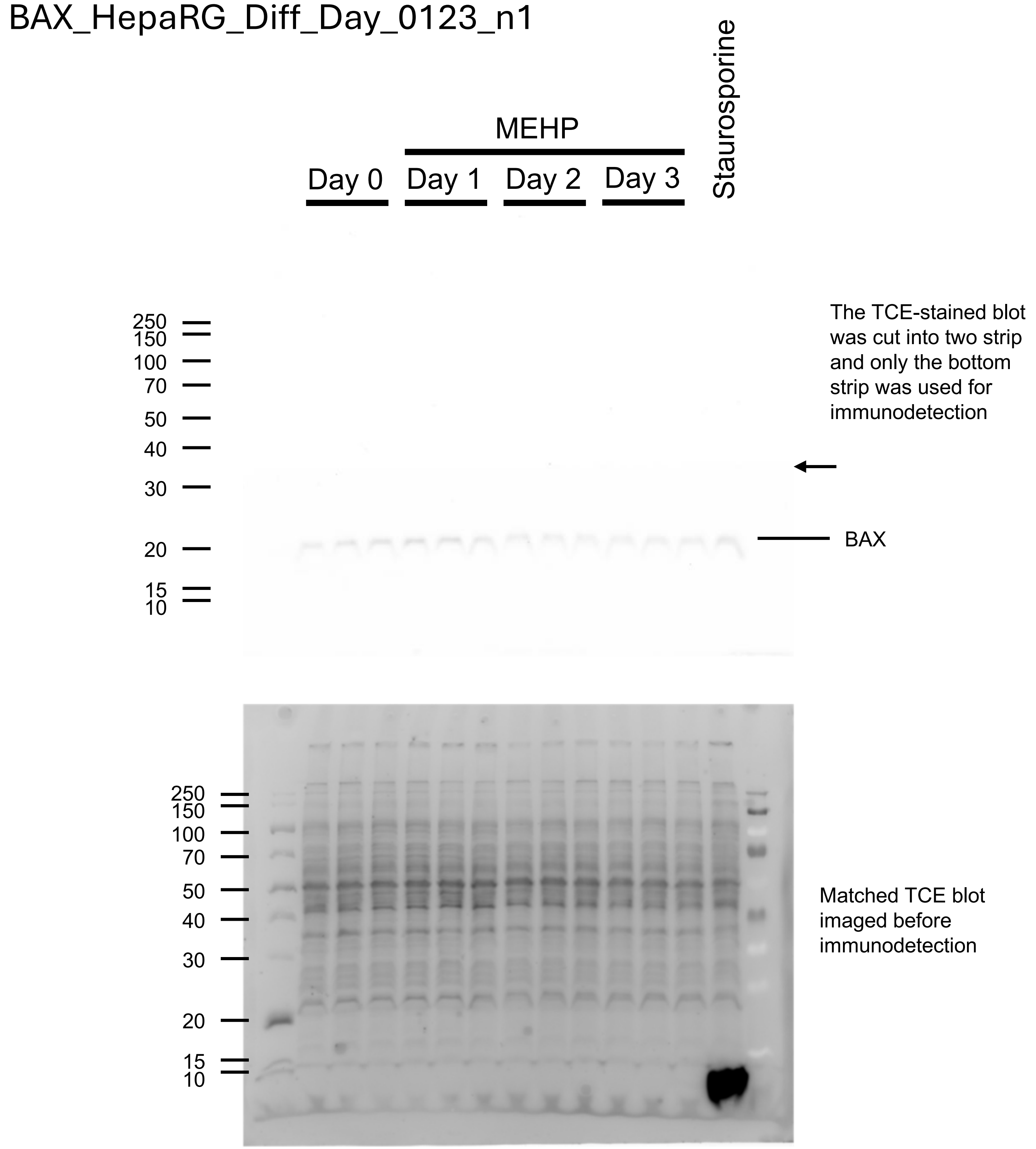

Supplementary Figure 31

BAX_HepaRG_Diff_Day_0123_n3

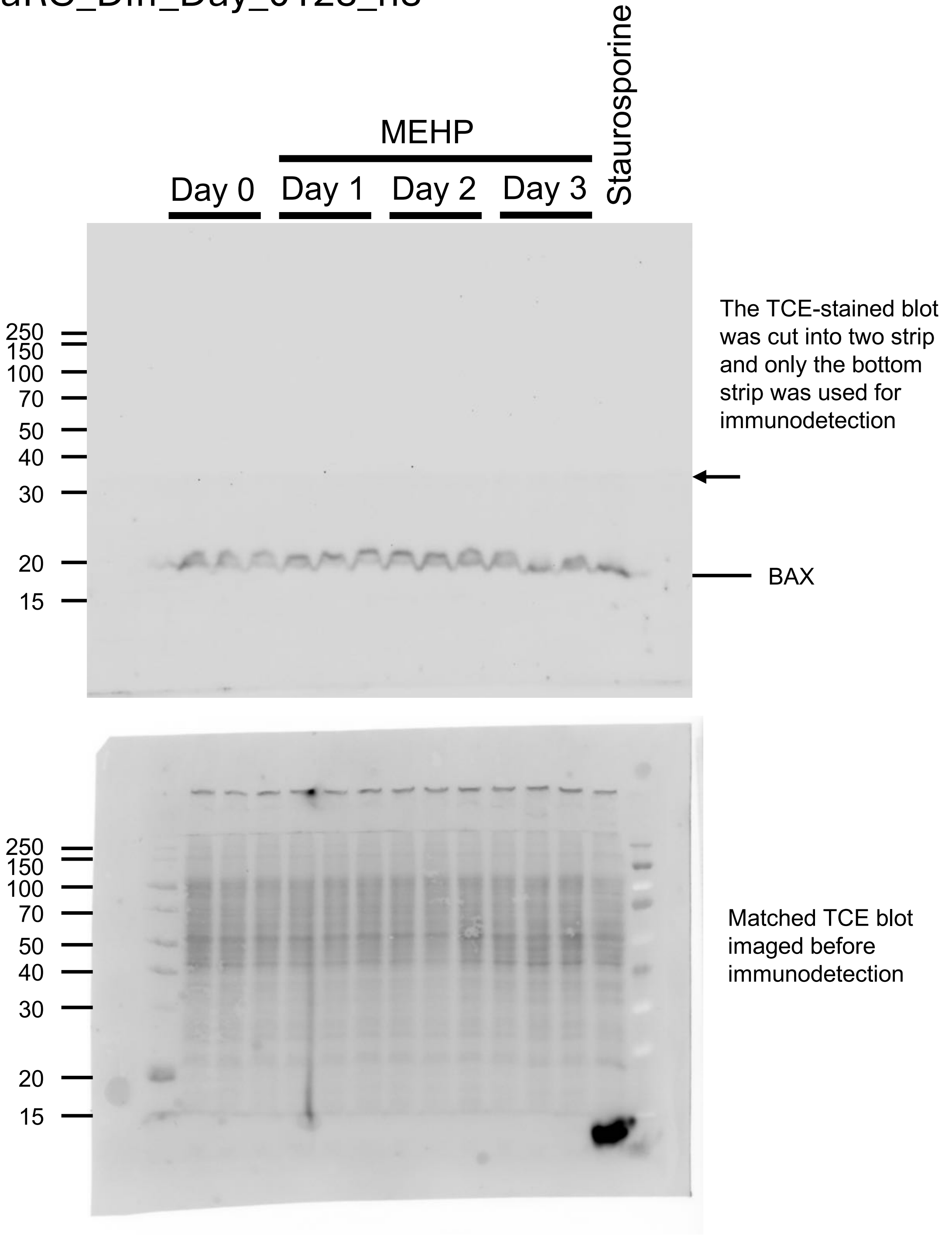

Supplementary Figure 32

BCL-2_HepaRG_unDiff_Day_0123_n3

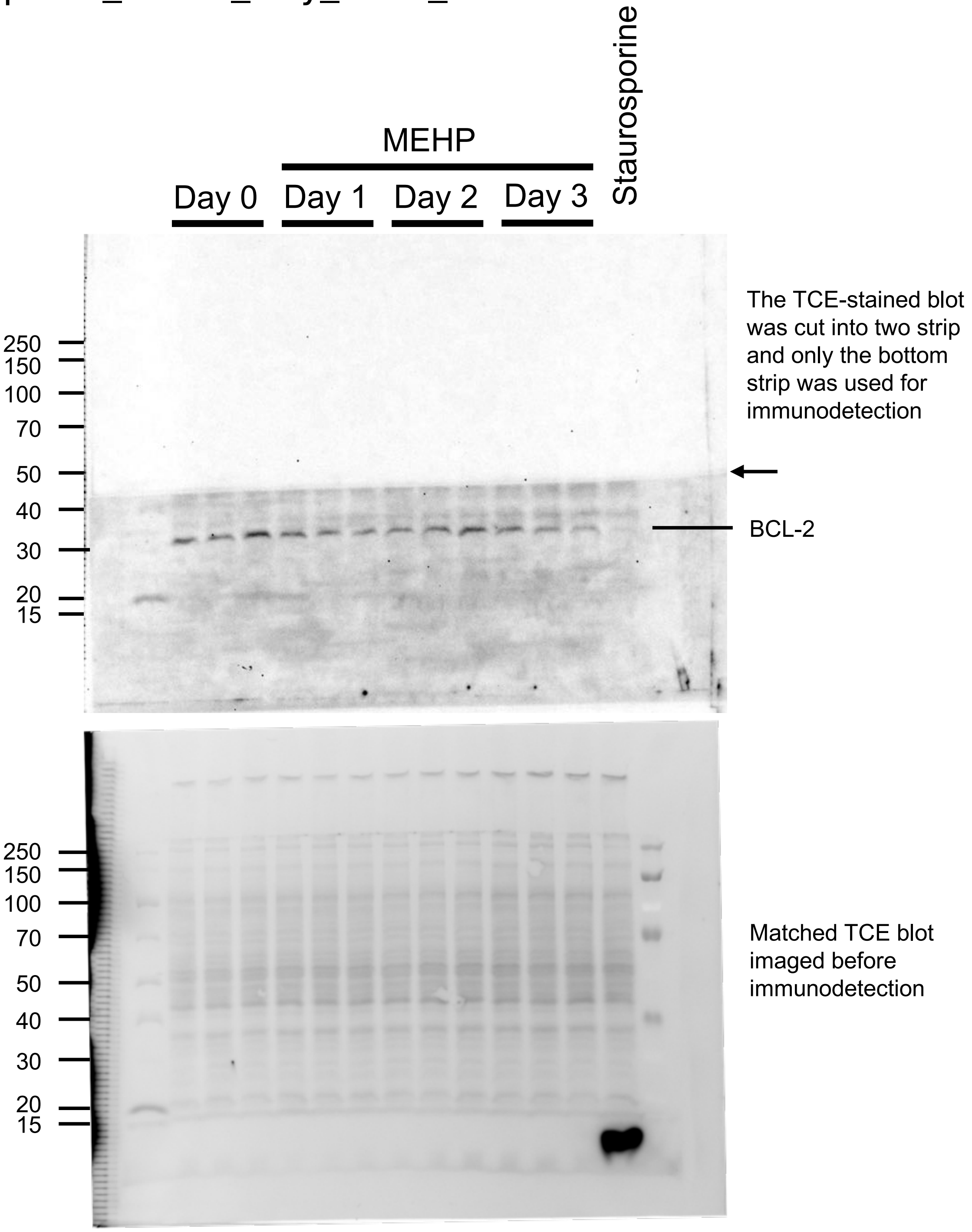

Supplementary Figure 33

BCL-2_HepaRG_unDiff_Day_0123_n1

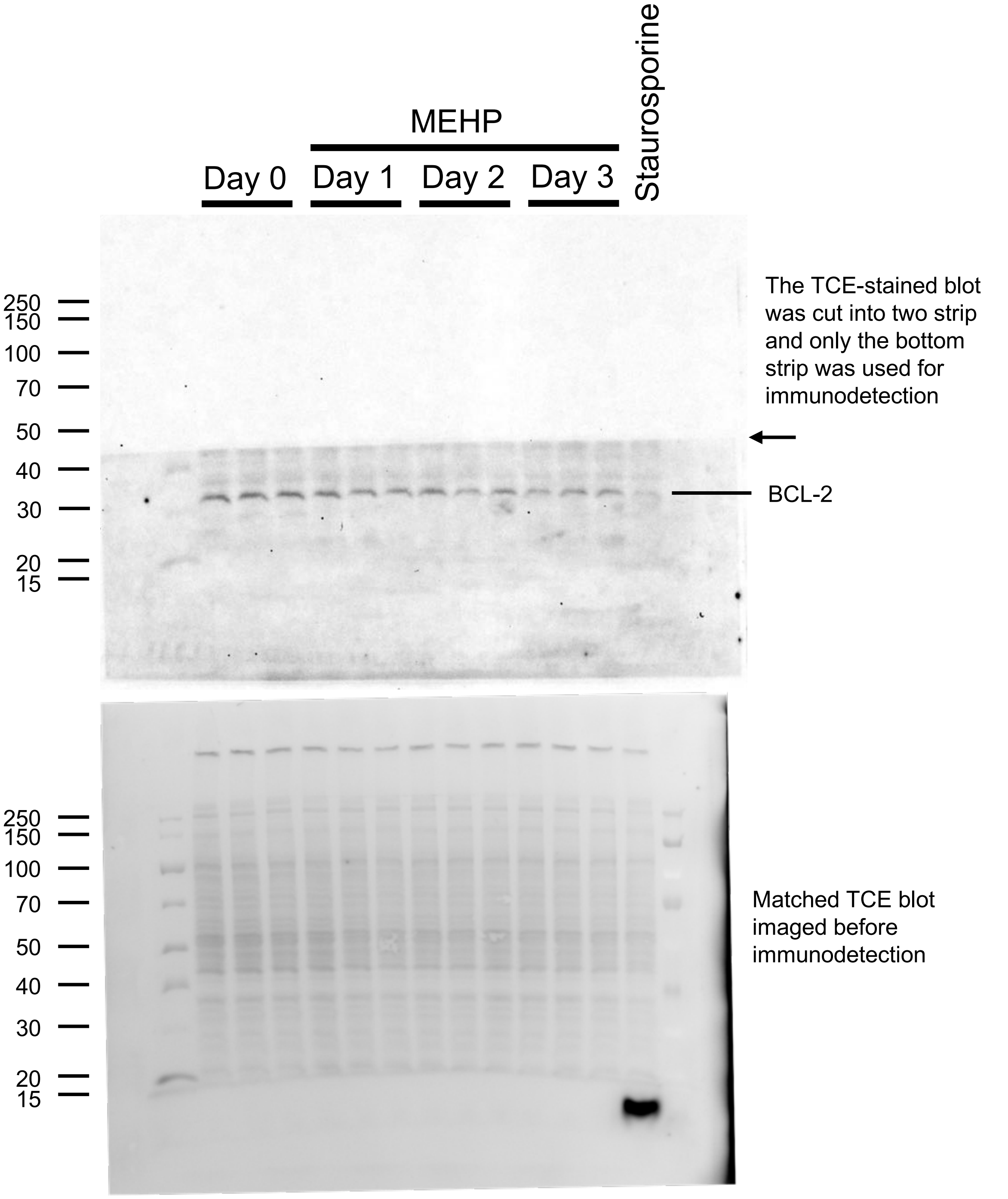

Supplementary Figure 34

BCL-2_HepaRG_Diff_Day_0123_n3

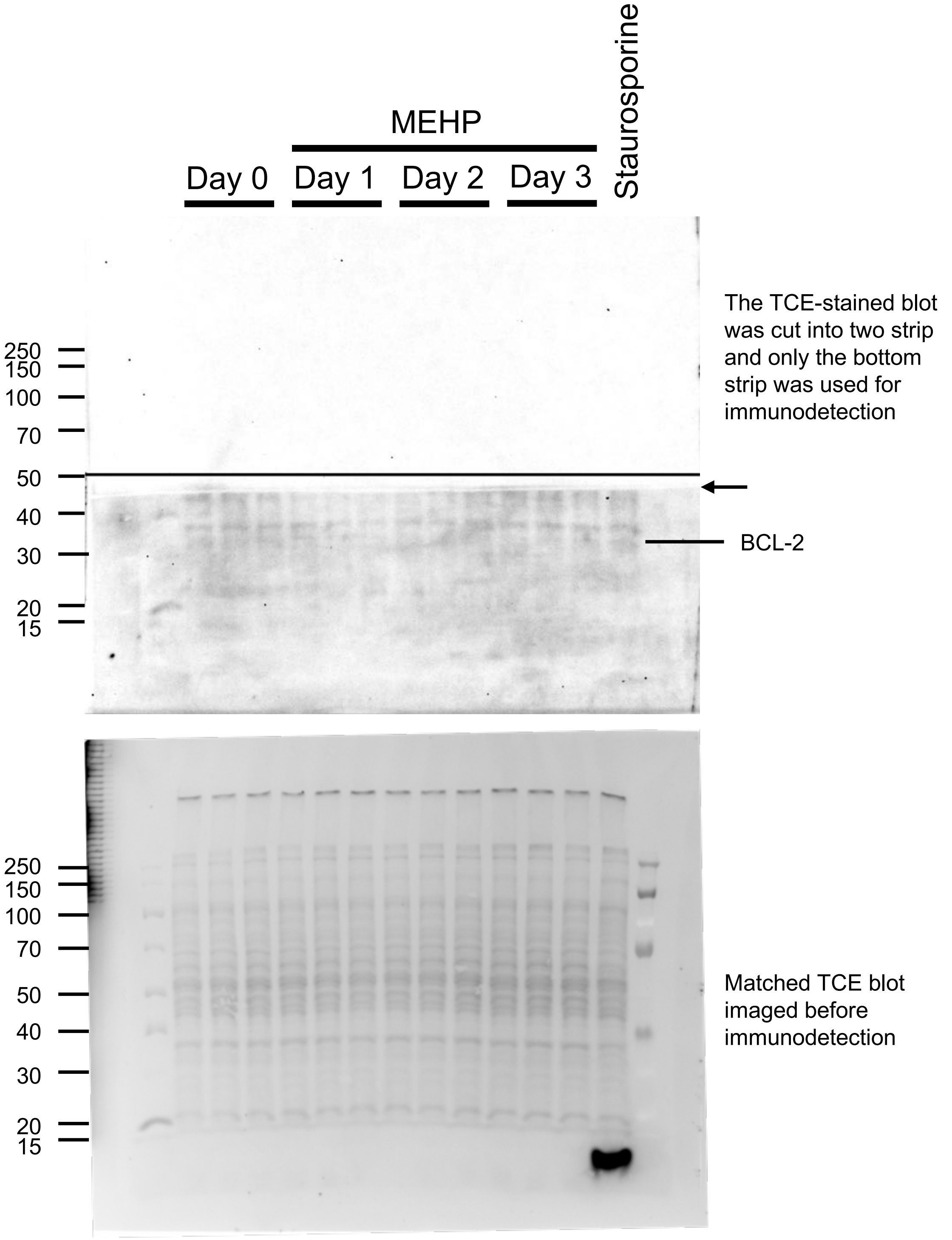

Supplementary Figure 35

BCL-2_HepaRG_Diff_Day_0123_n1

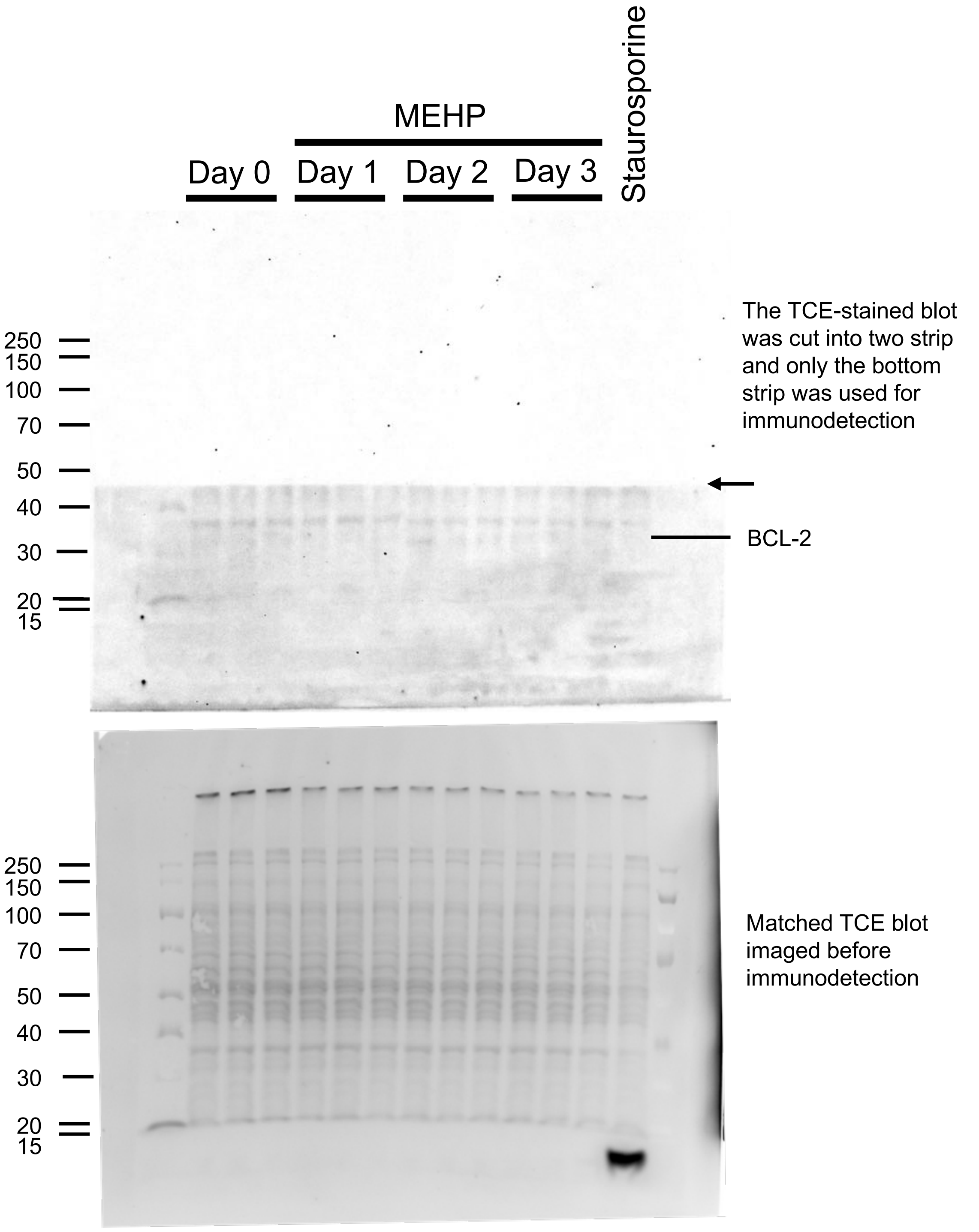

Supplementary Figure 36

p140_TWNK_MGME1_HepaRG diff_Day 6, 12_n1

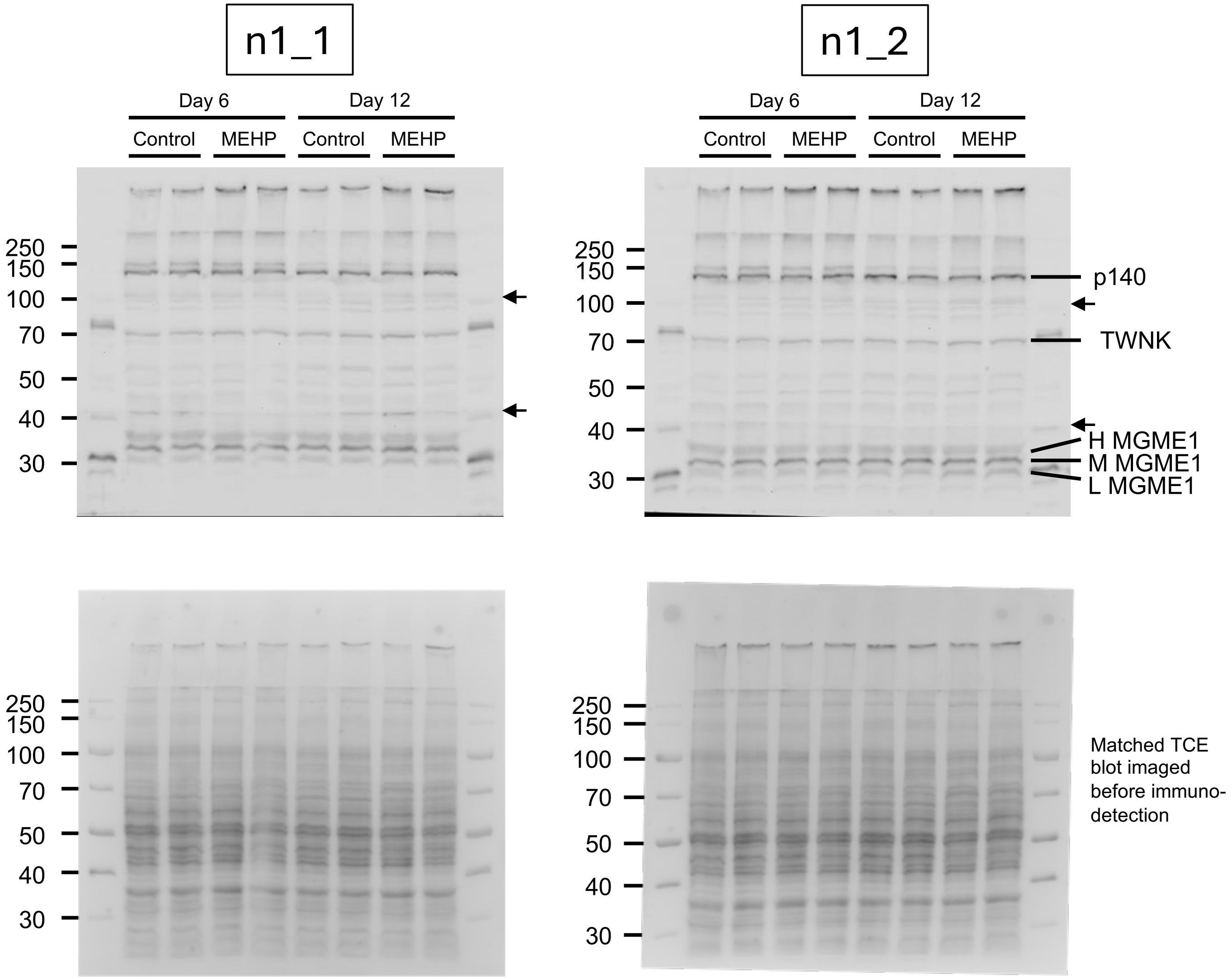

Blots containing day 6 and 12 exposure samples: Chemiluminescent and TCE-stained blots are shown on the top and bottom, respectively. Molecular weight standards are indicated on the left-hand side of the blots in kDa. The black arrows indicate the site of incision after TCE staining. WCE, Whole Cell Extract.

Supplementary Figure 37

p140_TWNK_MGME1_HepaRG diff_Day 6, 12_n2

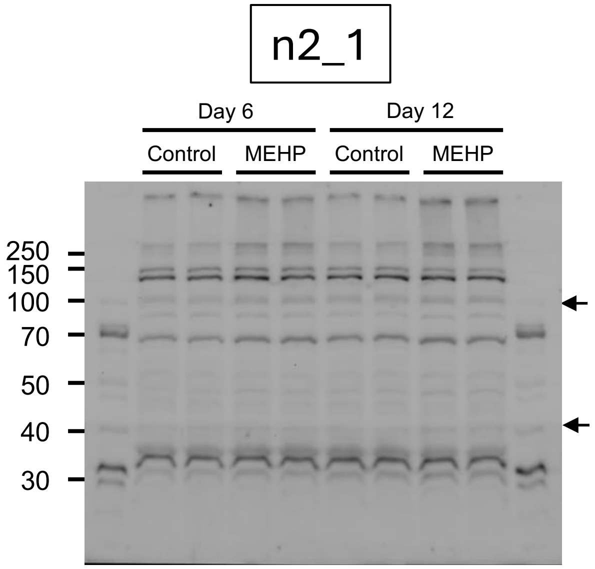

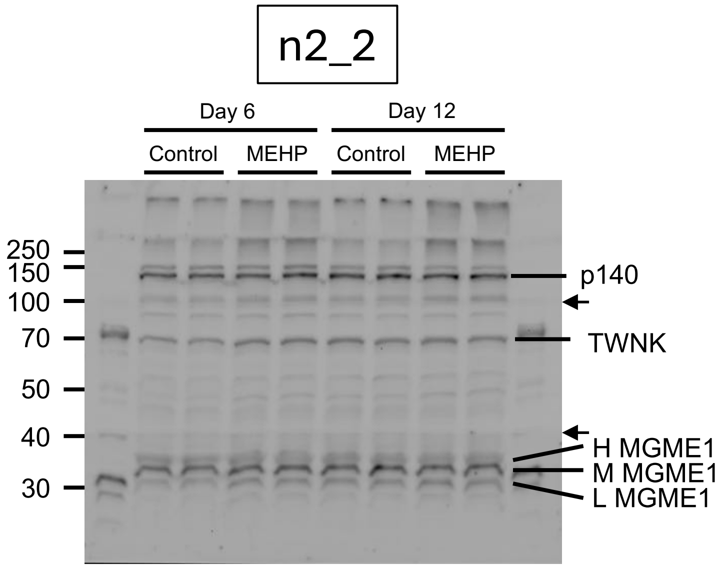

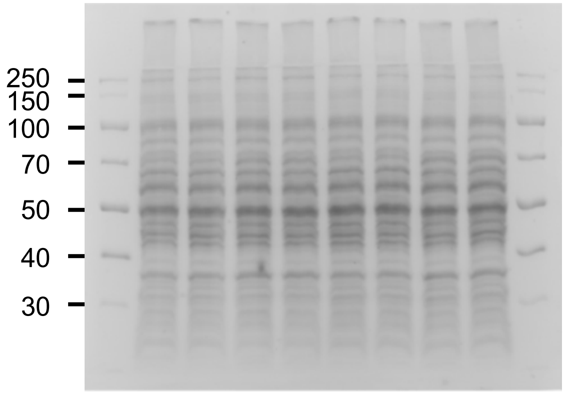

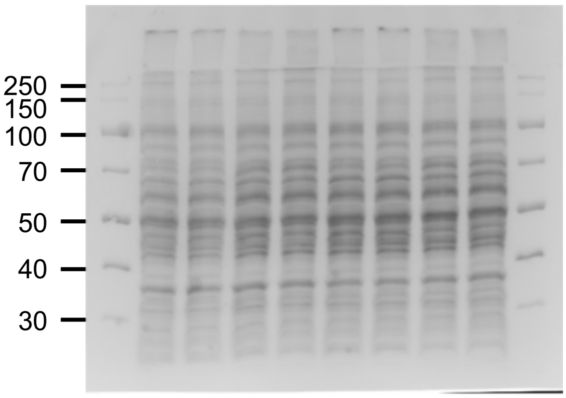

Matched TCE blot imaged before immuno-detection

Supplementary Figure 38

p140_TWNK_MGME1_HepaRG diff_Day 6, 12_n3

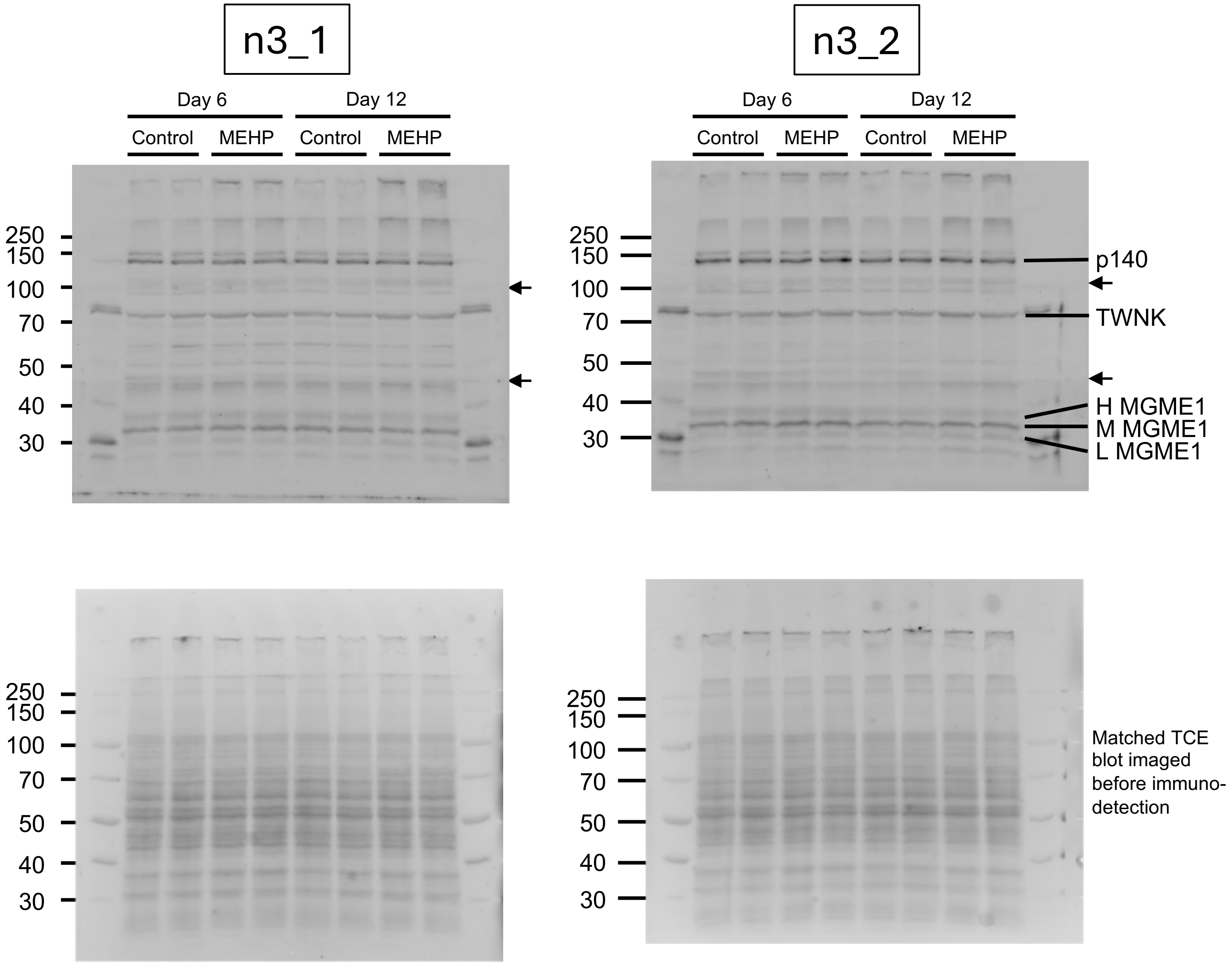

Supplementary Figure 39

p140_TWNK_MGME1_HepaRG prolif_Day 6, 12_n1

n1_1

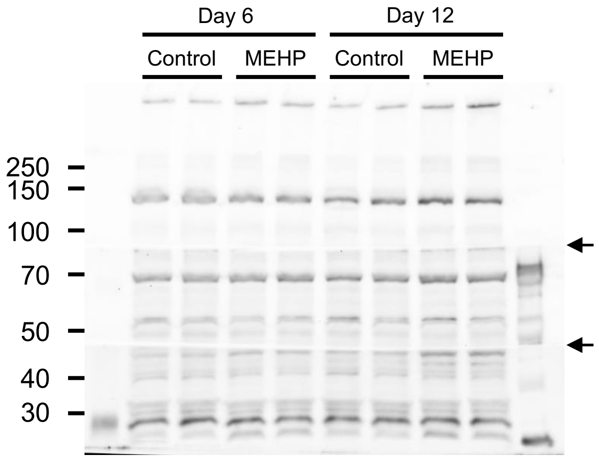

n1_2

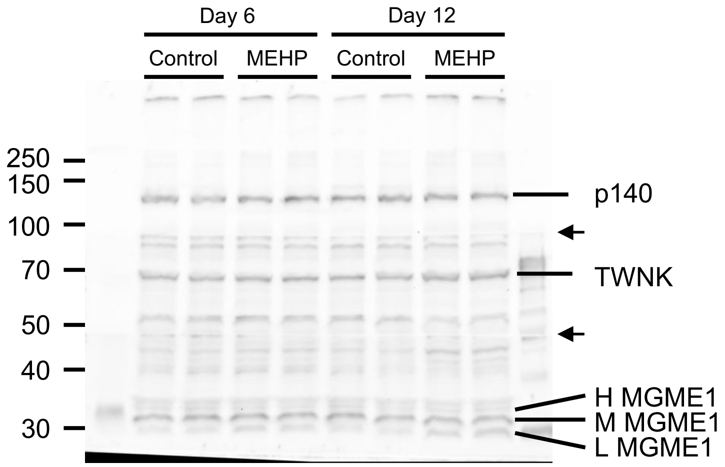

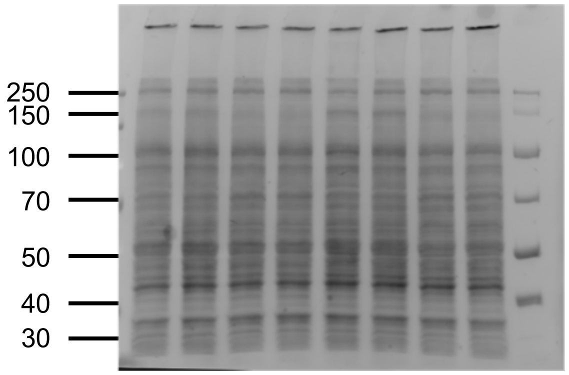

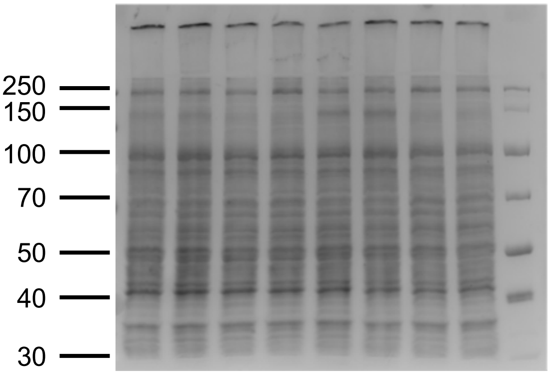

Matched TCE blot imaged before immuno-detection

Supplementary Figure 40

p140_TWNK_MGME1_HepaRG prolif_Day 6, 12_n2

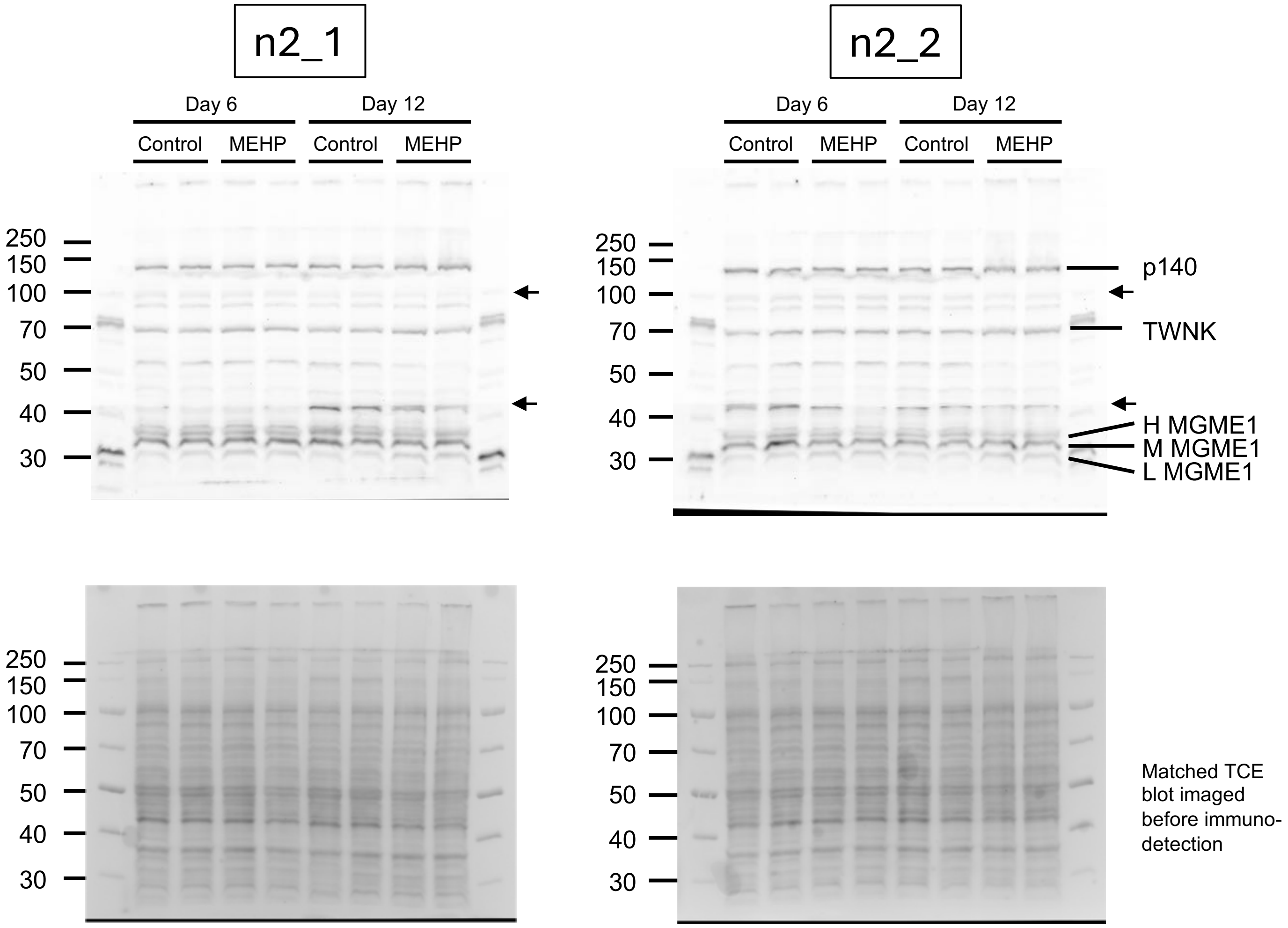

Supplementary Figure 41

p140_TWNK_MGME1_HepaRG prolif_Day 6, 12_n3

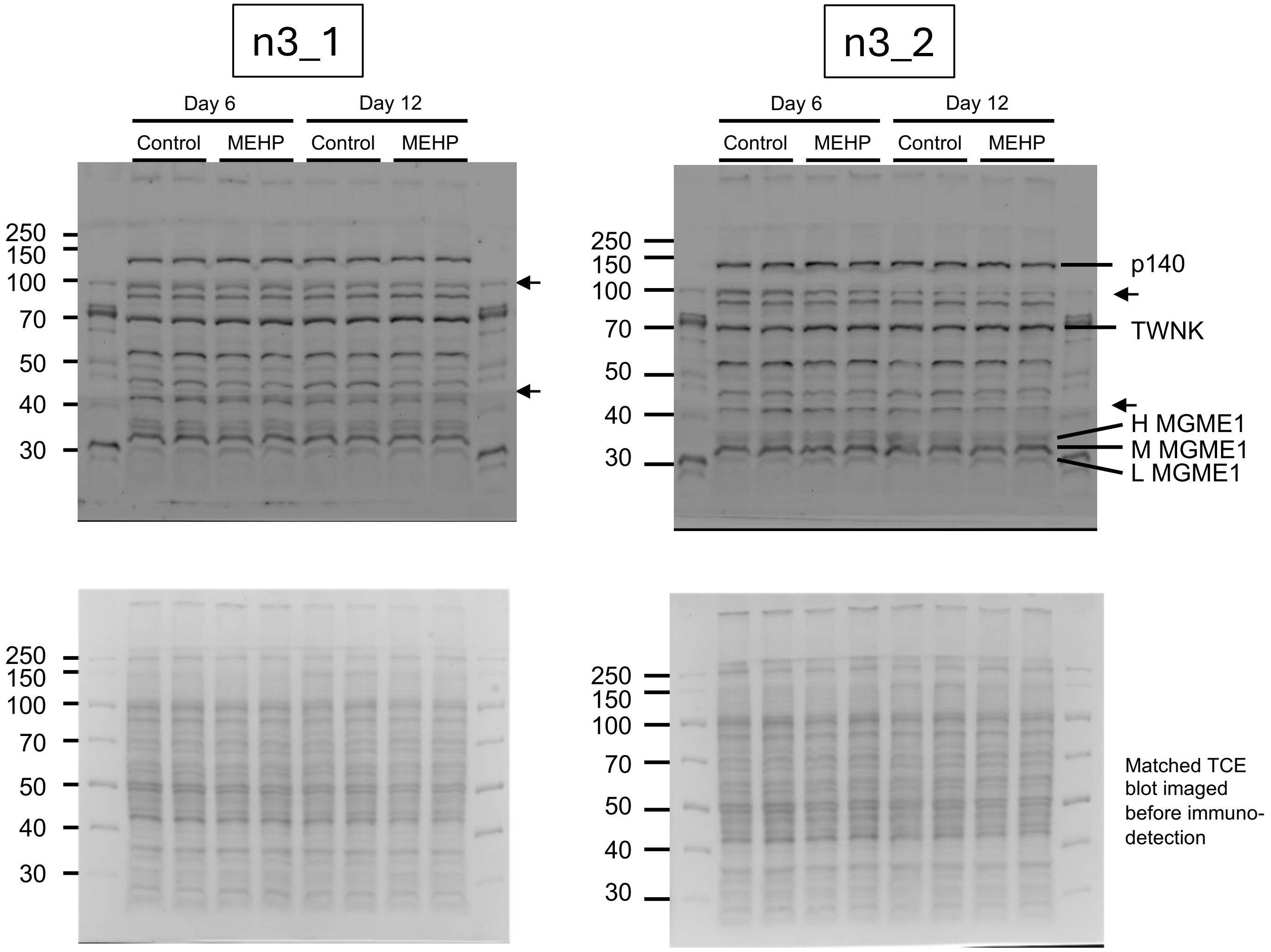

Supplementary Figure 42

p62_HepaRG diff_Day 6, 12_n1

n1_1

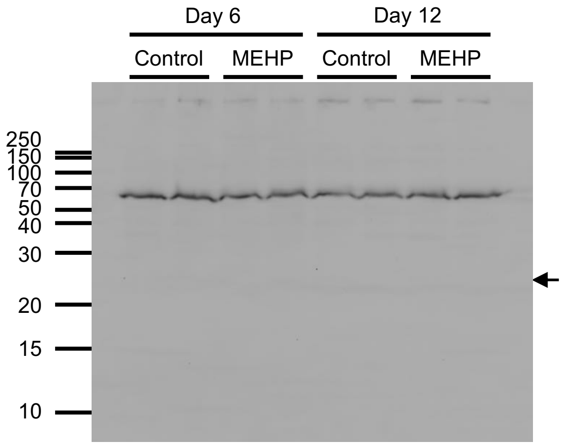

n1_2

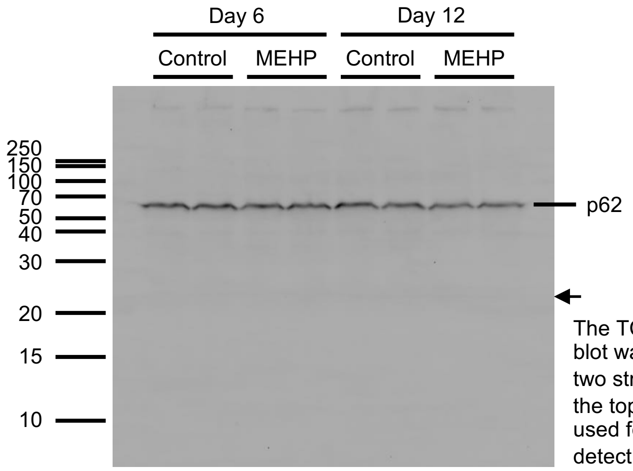

The TCE-stained blot was cut into two strip and only the top strip was used for immuno-detection

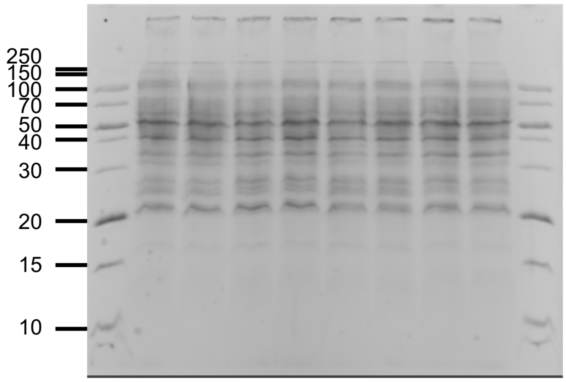

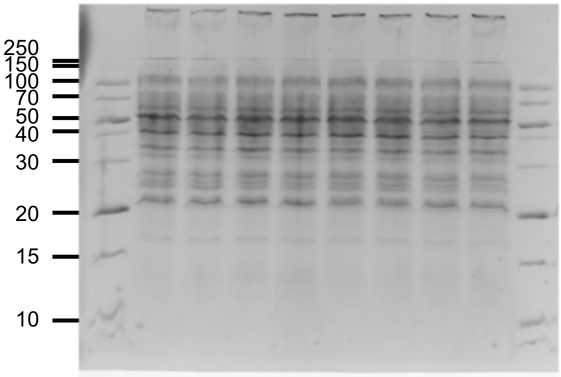

Matched TCE blot imaged before immuno-detection

Supplementary Figure 43

p62_HepaRG diff_Day 6, 12_n2

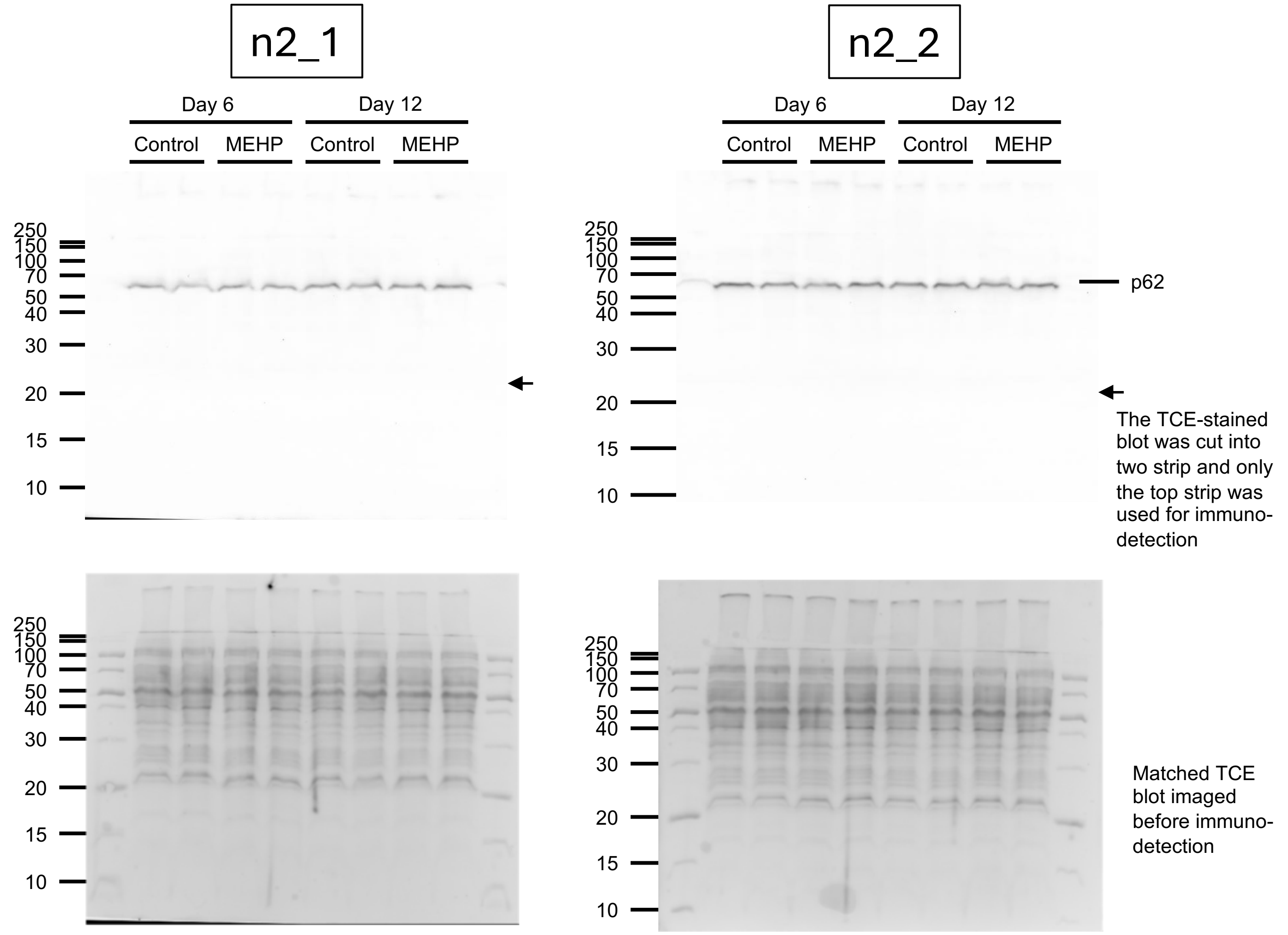

Supplementary Figure 44

p62_HepaRG diff_Day 6, 12_n3

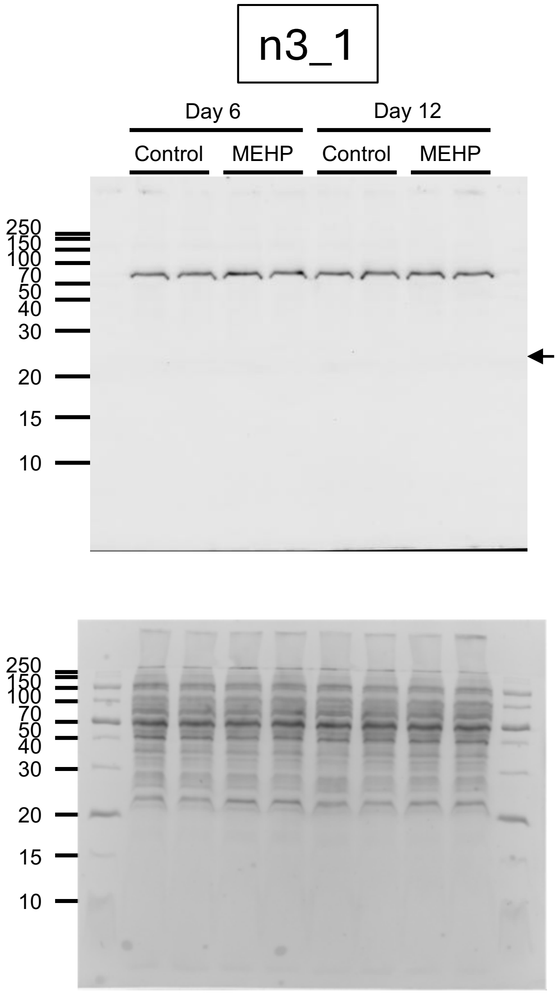

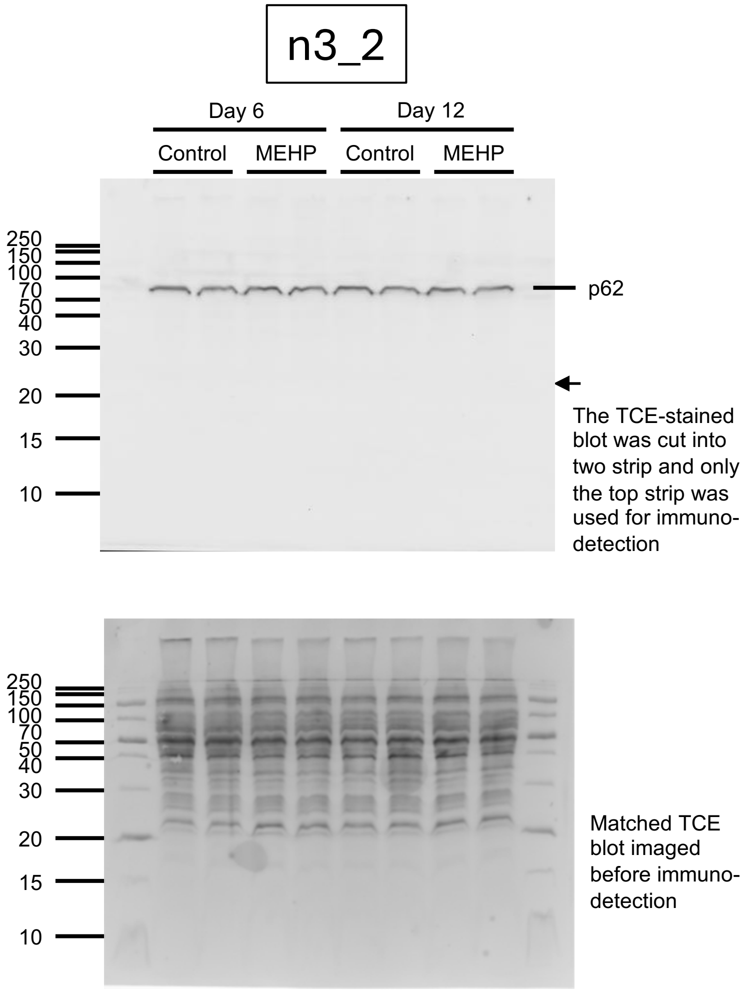

Supplementary Figure 45

p62_HepaRG prolif_Day 6, 12_n1

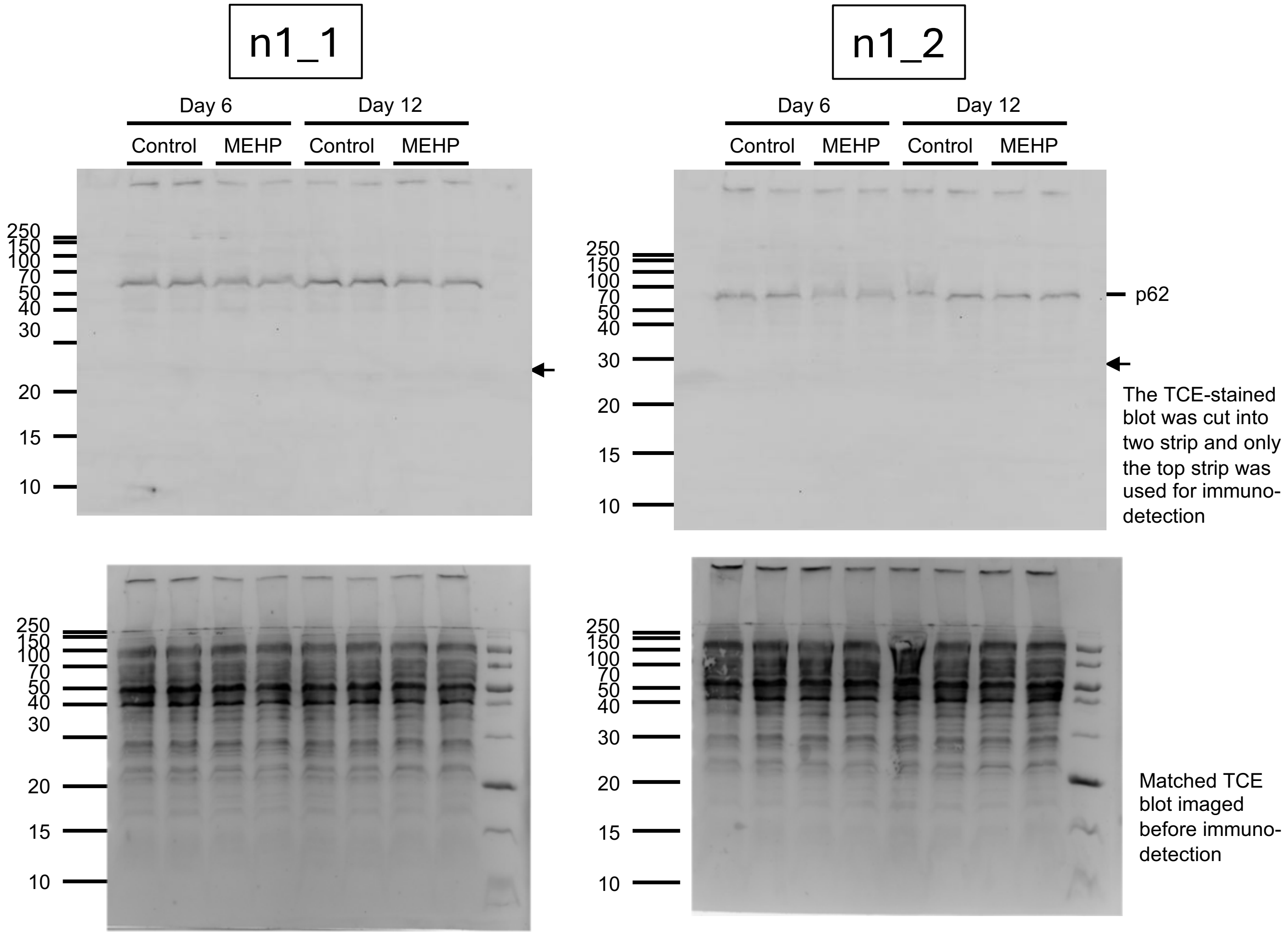

Supplementary Figure 46

p62_HepaRG prolif_Day 6, 12_n2

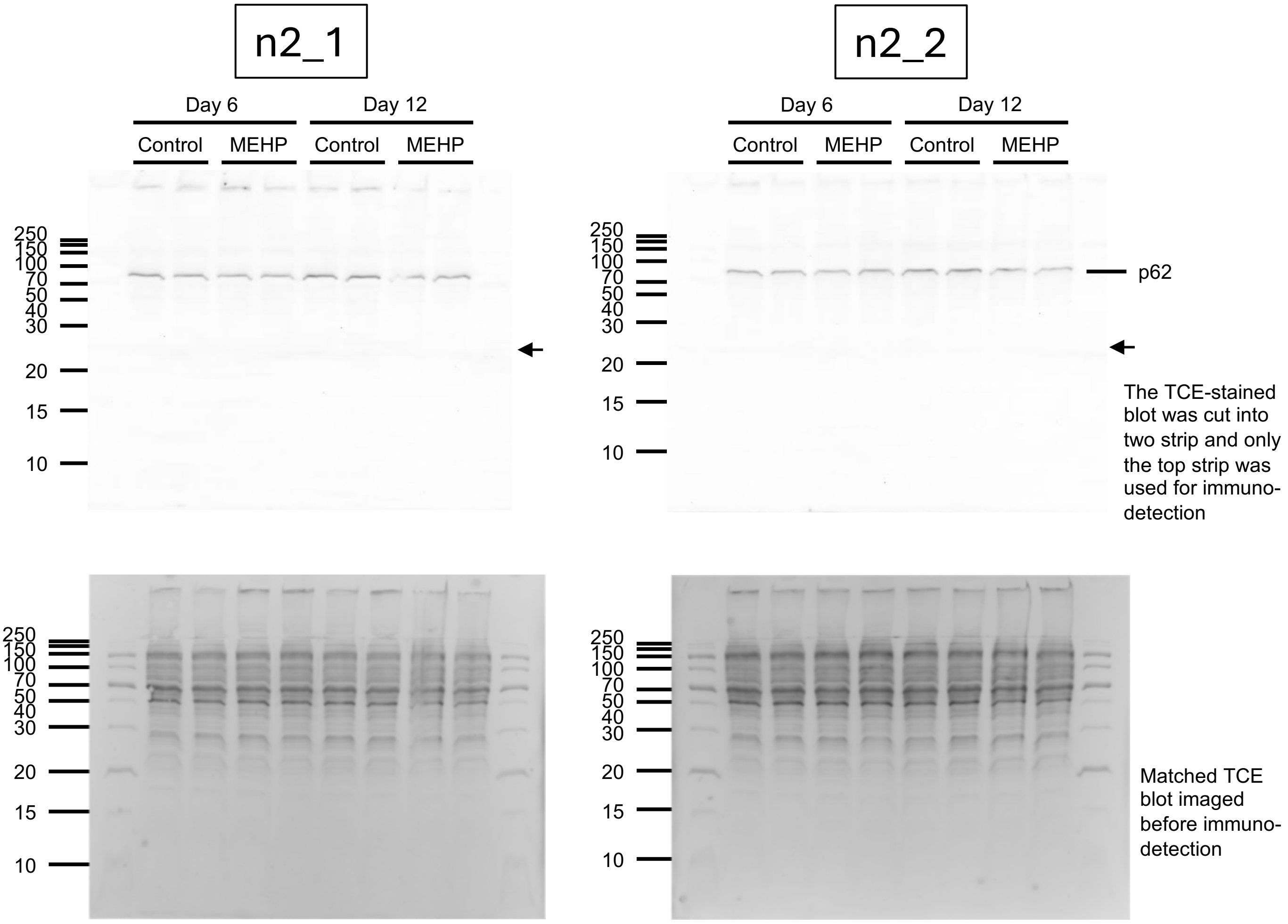

Supplementary Figure 47

p62_HepaRG prolif_Day 6, 12_n3

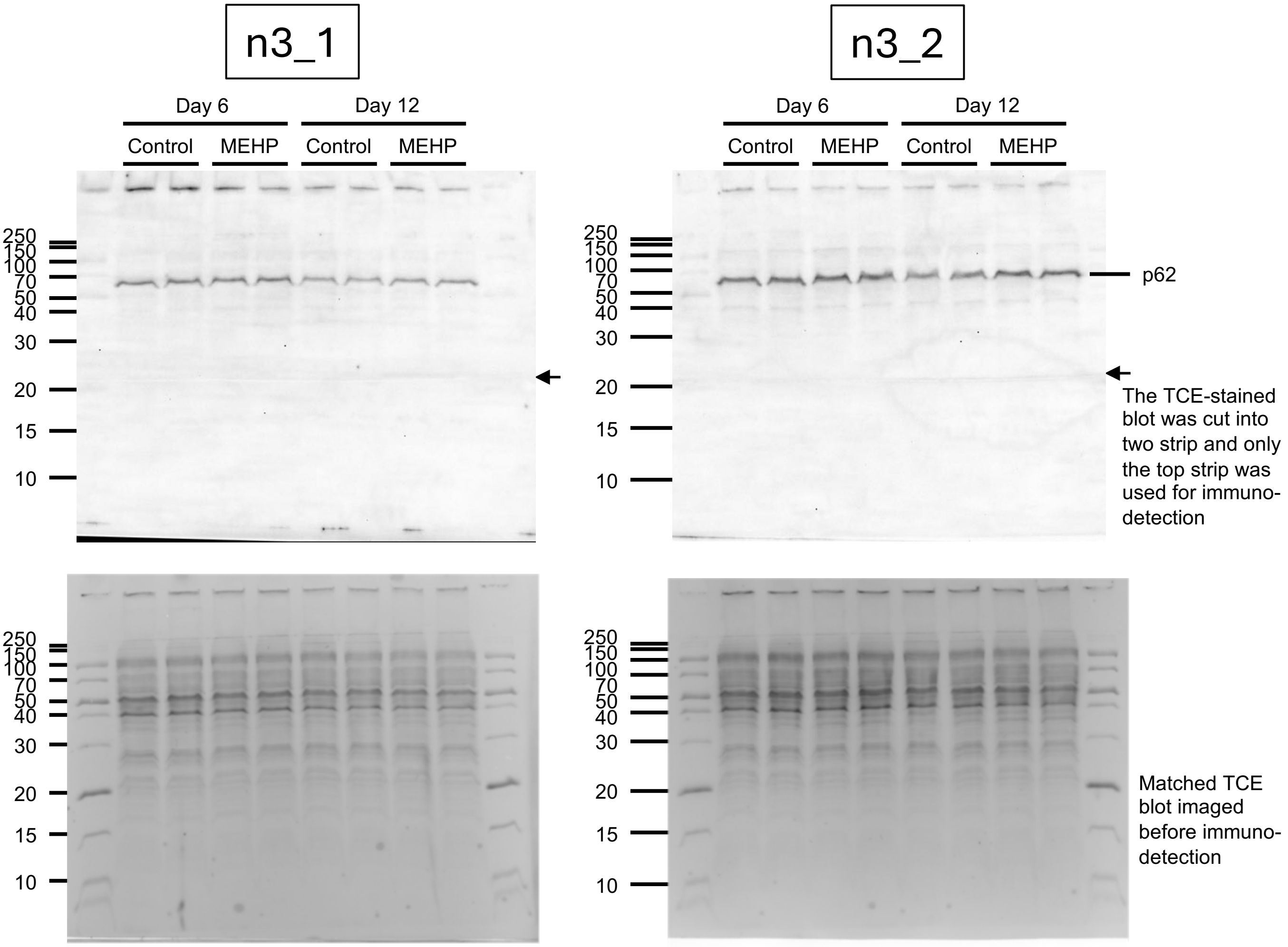

Supplementary Figure 48

PINK1_HepaRG prolif_Day 6, 12_n1 and n2

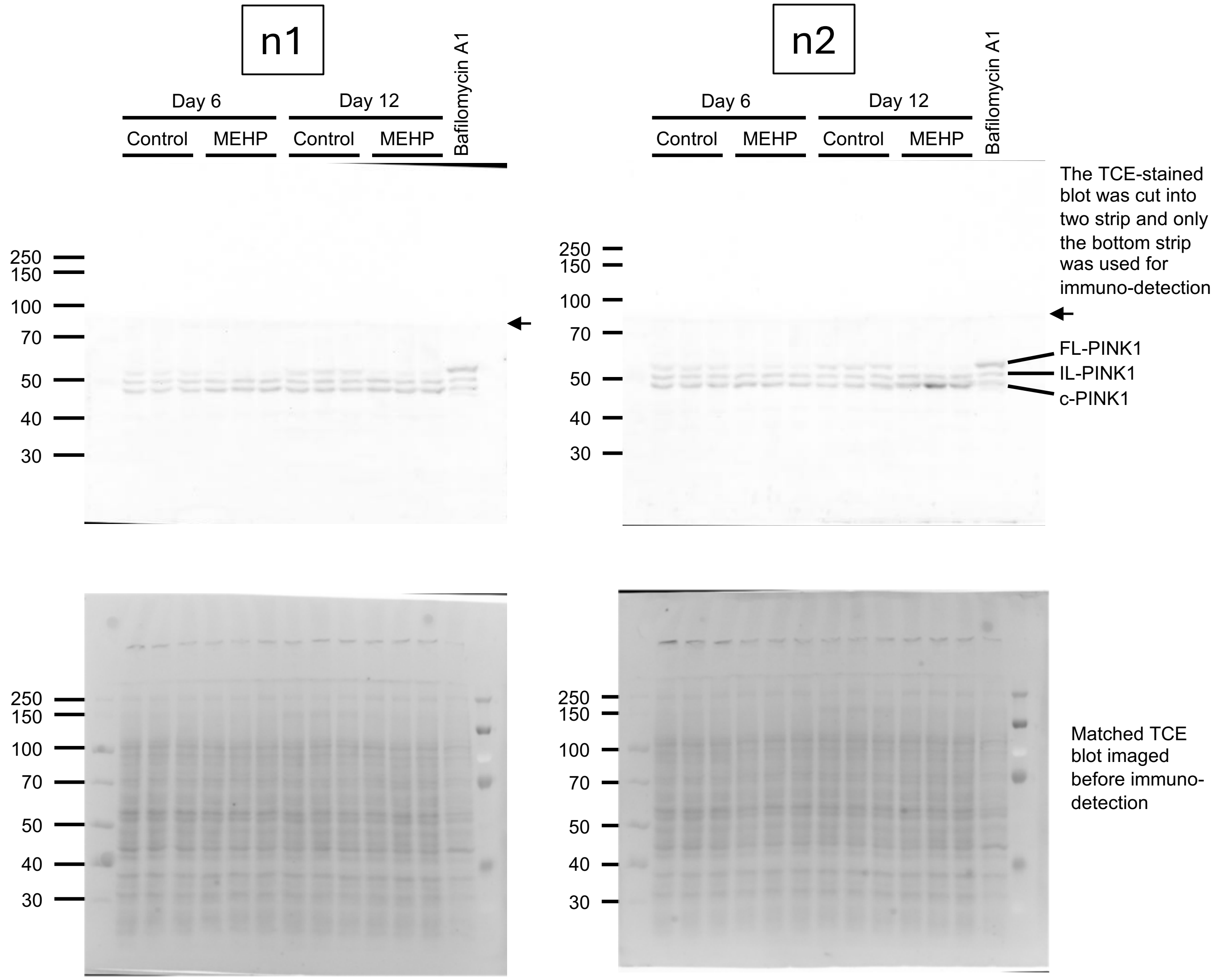

Bafilomycin A1: Cells were treated with 100μM Bafilomycin A1 for 24 hours and whole cell protein extract was run on the corresponding lane.

Supplementary Figure 49

PINK1_HepaRG prolif_Day 6, 12_n3

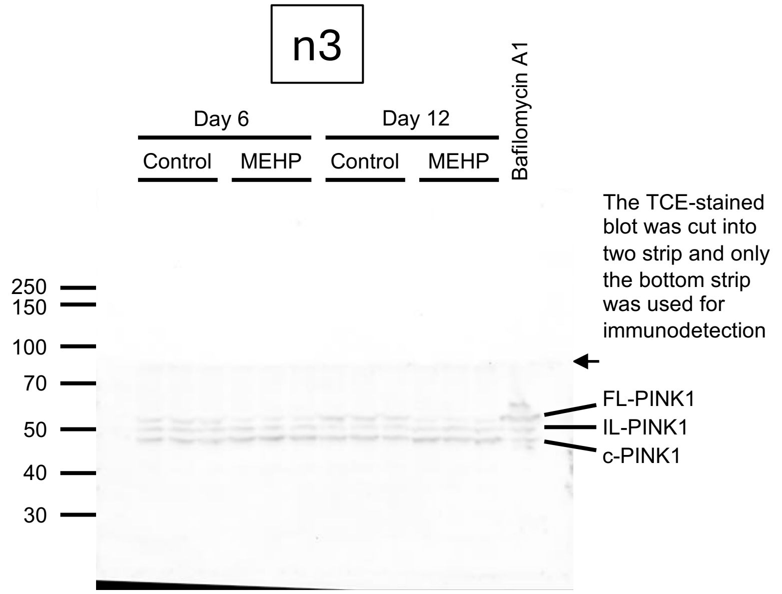

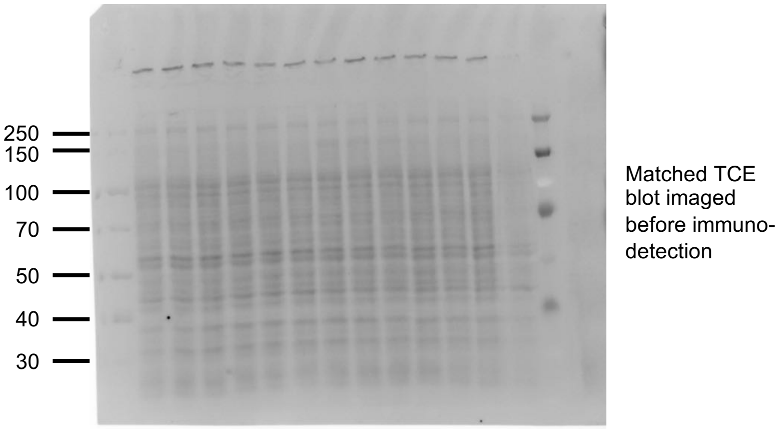

Supplementary Figure 50

PINK1_HepaRG diff_Day 6, 12_n1 and n2

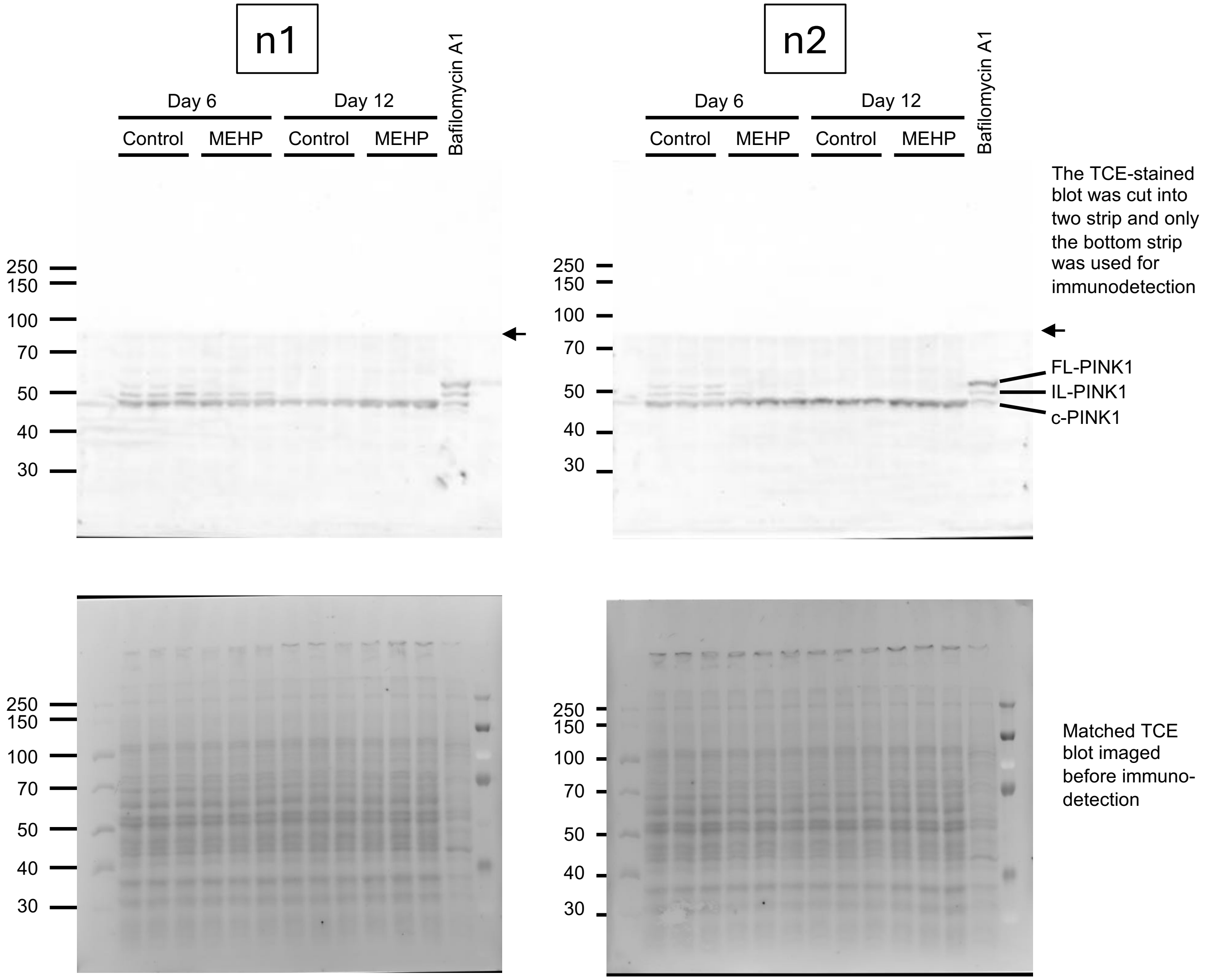

Supplementary Figure 51

PINK1_HepaRG diff_Day 6, 12_n3

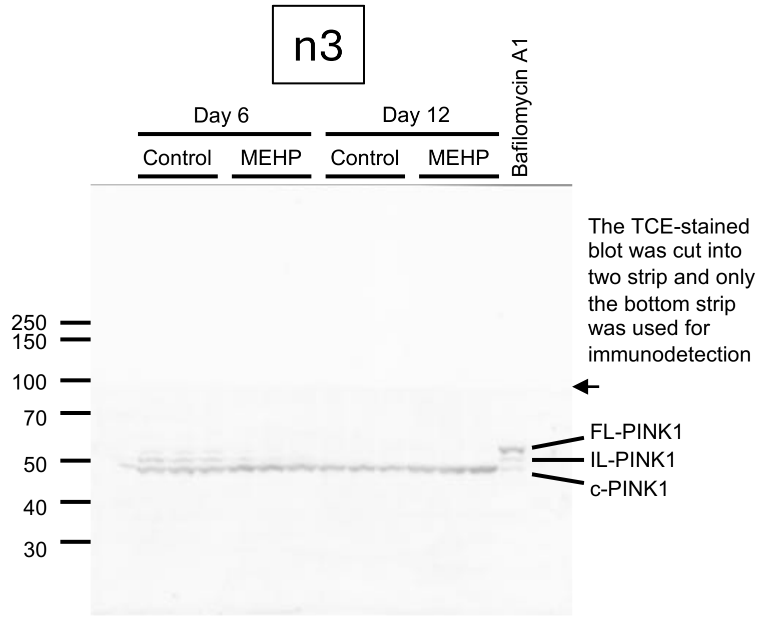

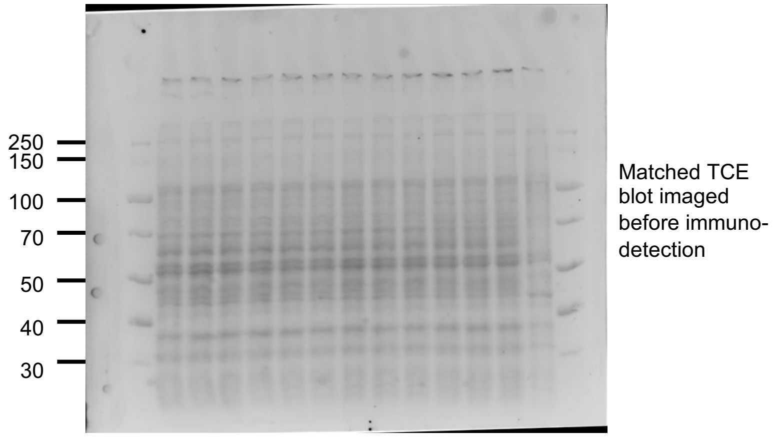

Supplementary Figure 52

Optimization of MGME1 Ab

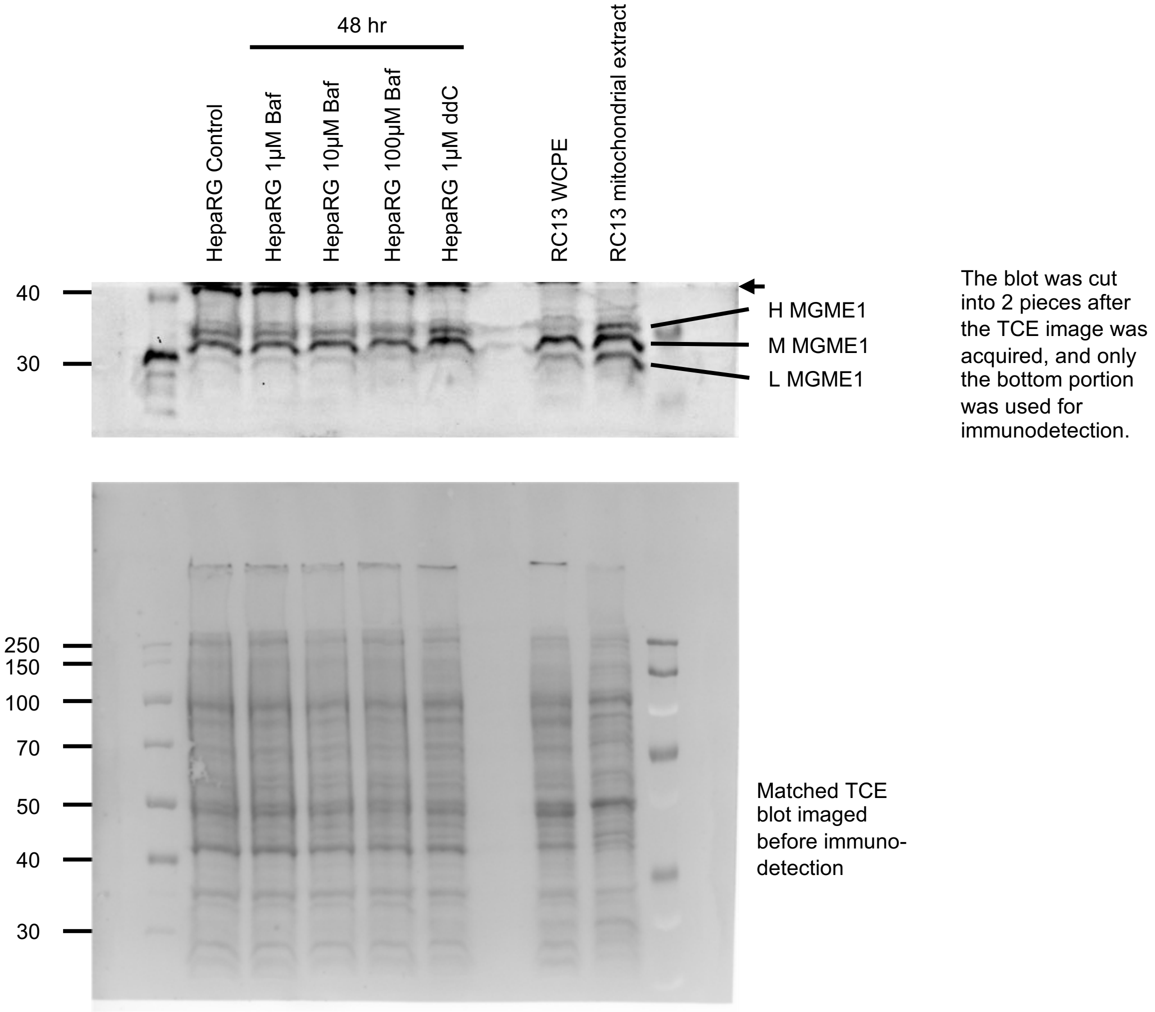

Optimization of MGME1 Antibody (Ab). Whole cell protein extracts (WCPEs) were prepared and analyzed from mock-treated (control), 1µM ddC treated, and 1, 10, 100 µM Bafilomycin A1 (Baf) treated HepaRG cells. Additionally, RC13 whole-cell protein extract (WCPE) and mitochondrial extracts were used to assess enrichment of mitochondria-targeted proteins. Enrichment of the three MGME1 isoforms was observed in the mitochondrial extract. The black arrow denotes the place where the blot was sliced after taking a TCE-stained image.

Supplementary Figure 53

Optimization of TWNK Ab

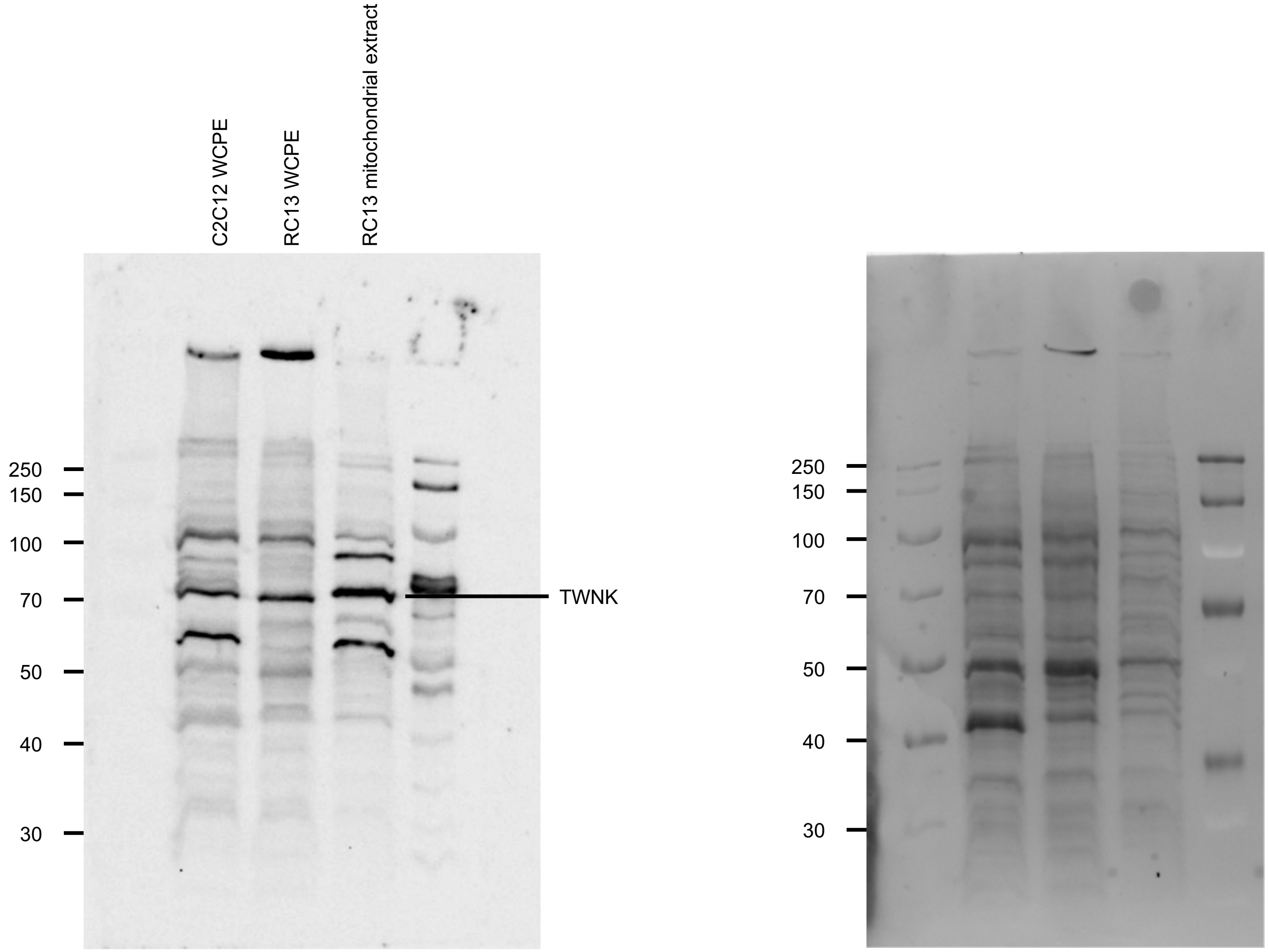

Matched TCE blot imaged before immunodetection

Optimization of TWNK Antibody (Ab). For optimization, C2C12 and RC13 whole-cell protein extracts (WCPE) were used. Additionally, an RC13 mitochondrial extract was used to determine enrichment of mitochondria-targeted proteins. Enrichment of the TWNK band was observed in the mitochondrial extract.

Supplementary Figure 54

Optimization of p140 Ab

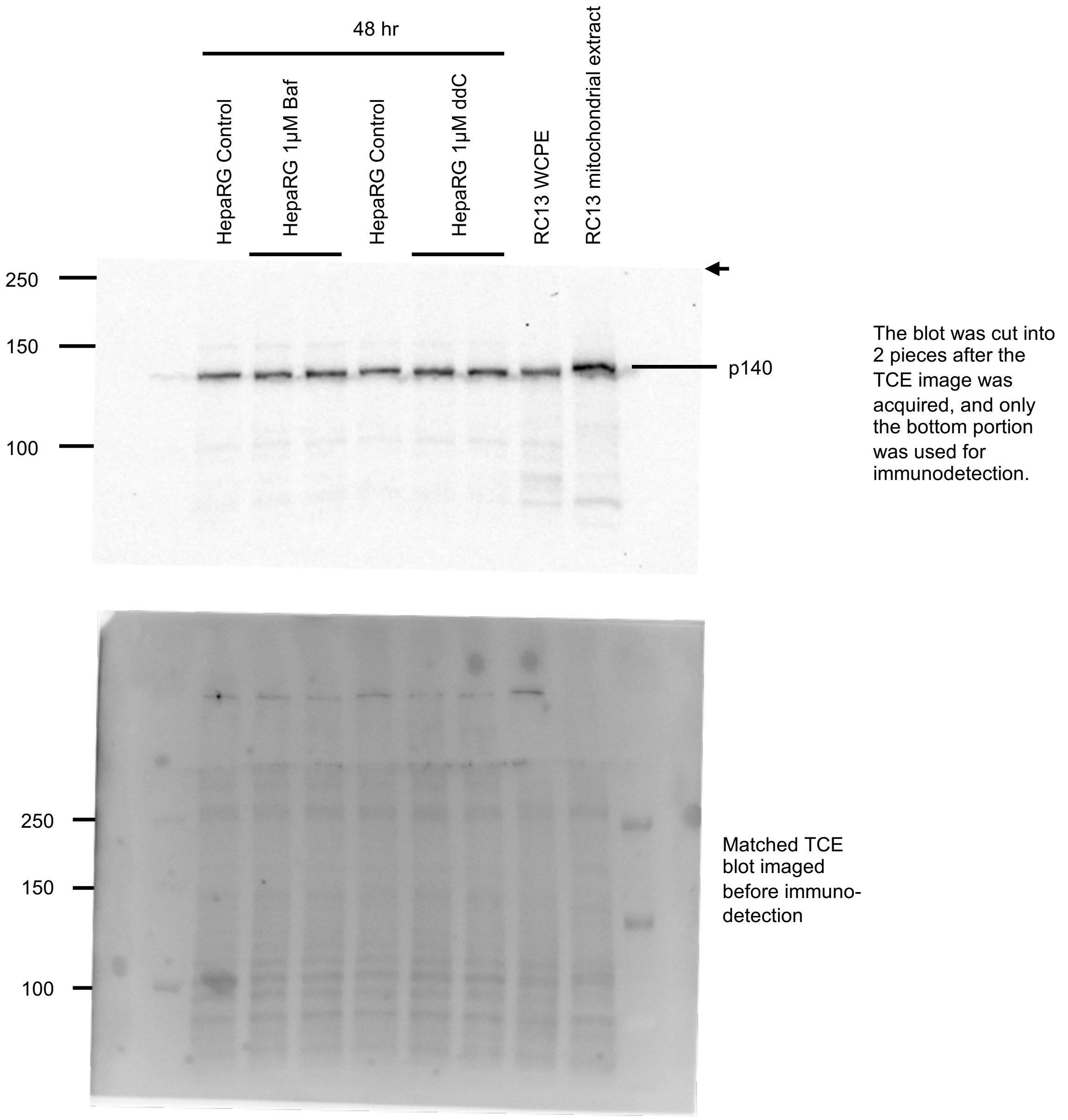

Optimization of p140 Antibody (Ab). Whole-cell protein extracts (WCPEs) were prepared and analyzed from mock-treated (control), 1 µM ddC, and 1 µM Bafilomycin A1 (Baf)- treated HepaRG cells. Additionally, an RC13 WCPE and a mitochondrial extract were used to see enrichment of mitochondria-targeted proteins. Enrichment of p140 was observed in the mitochondrial extract. The black arrow denotes the places where blots were sliced after taking a TCE-stained image.

Supplementary Figure 55

A. Undifferentiated Day 13

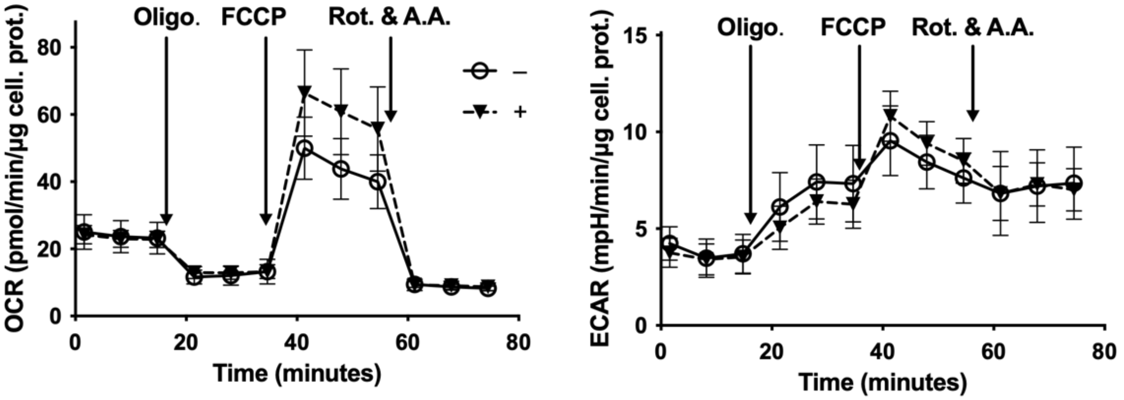

B. Differentiated Day 7

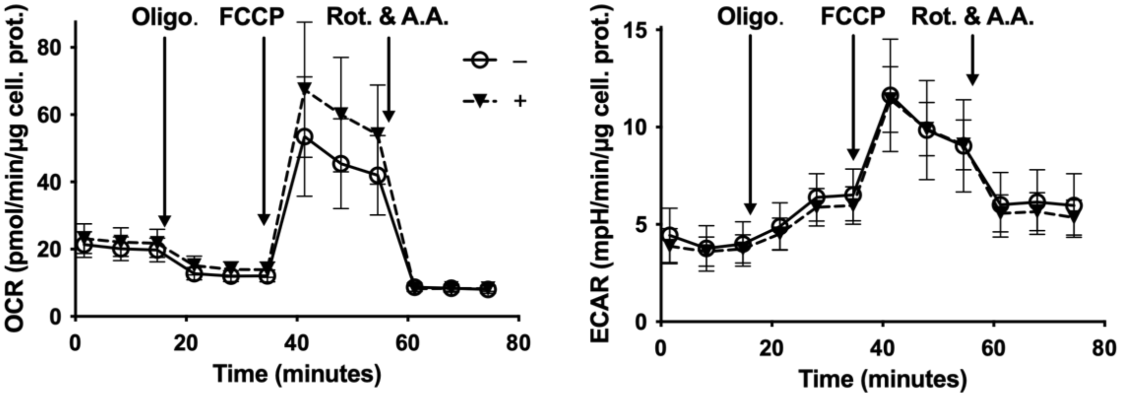

C. Differentiated Day 13

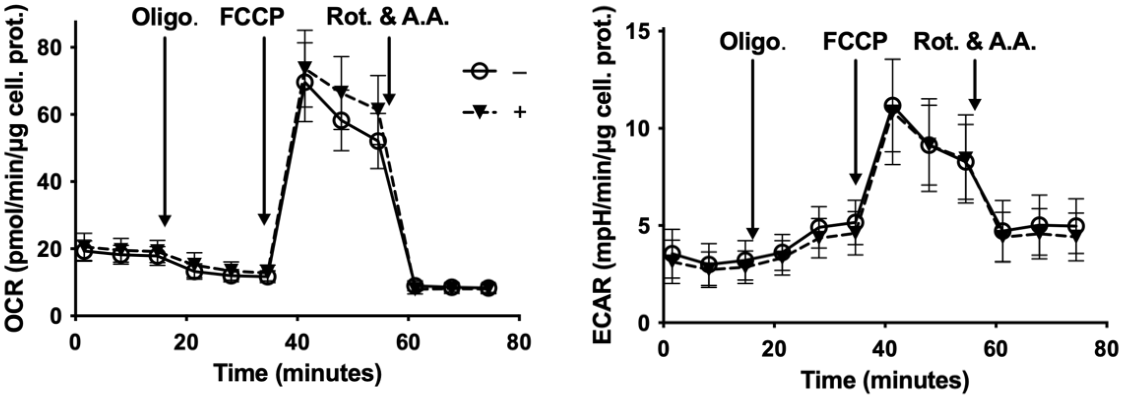

Supplementary Figure 56

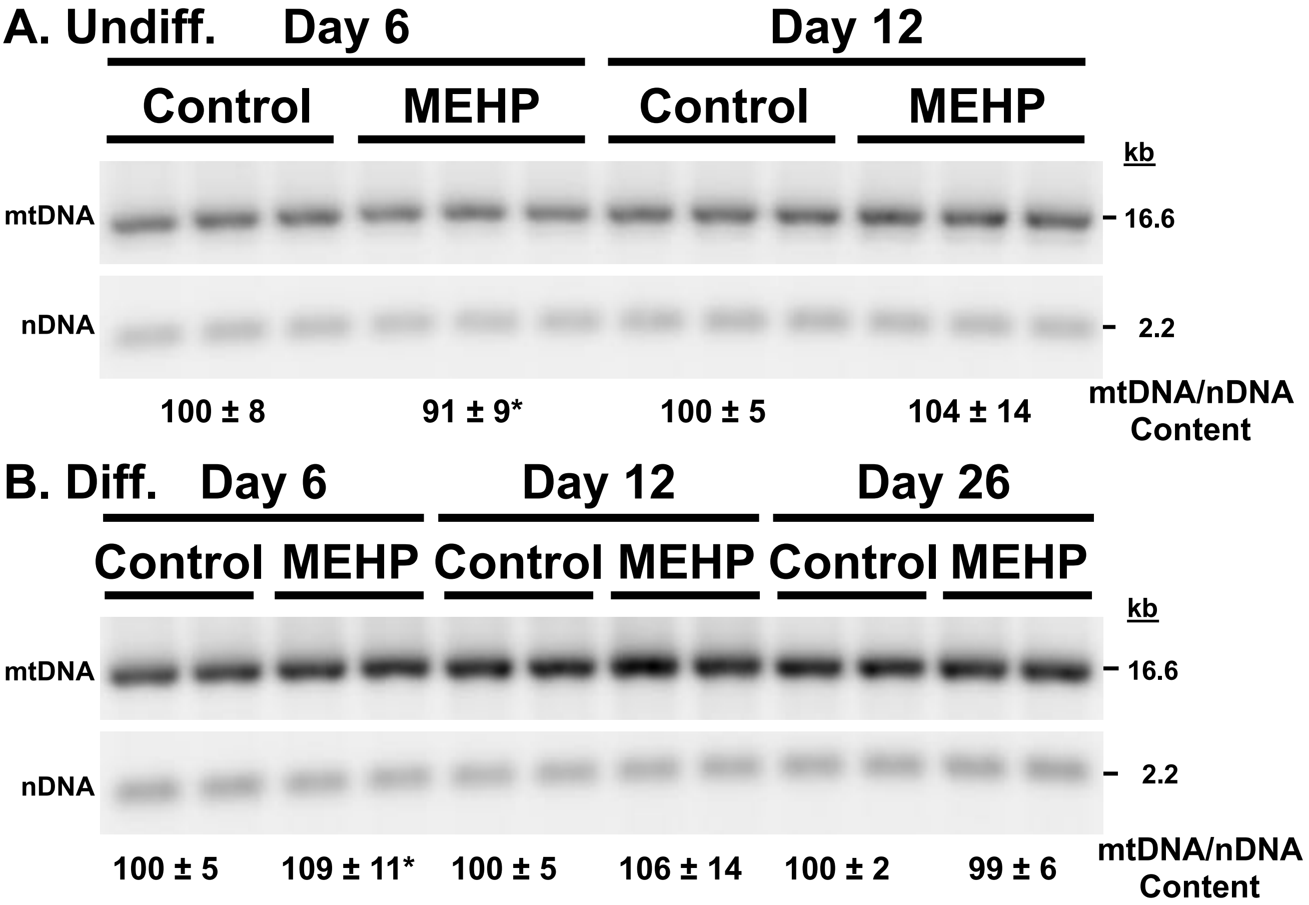

Supplementary Figure 57

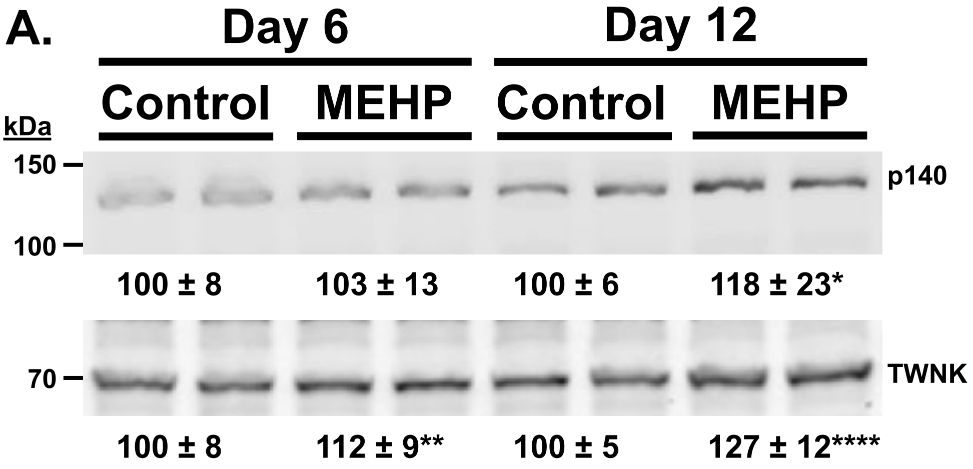

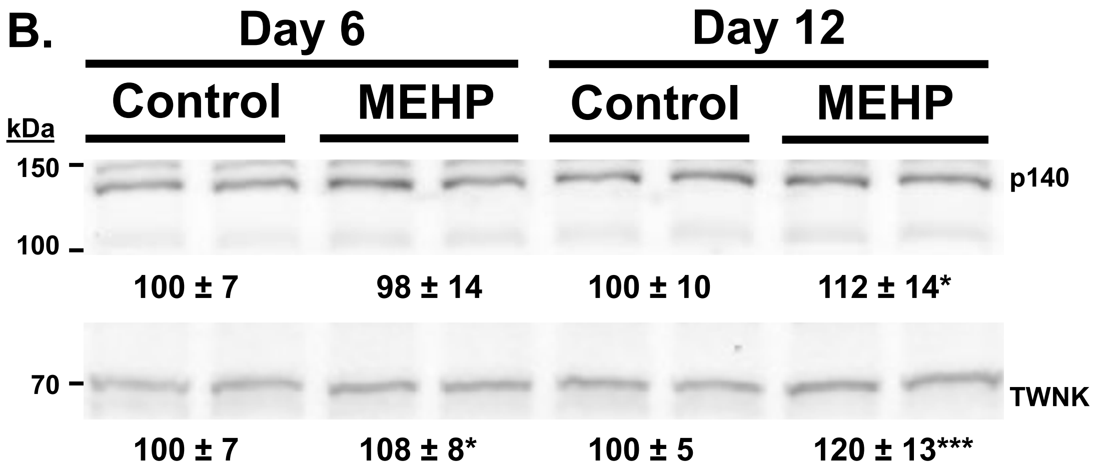

Supplementary Figure 58
